# Psychological assessments predict insider threat risk-mitigation measures, but only marginally

**DOI:** 10.3389/fpsyg.2026.1926403

**Published:** 2026-09-08

**Authors:** Kristoffer Lilja-Lolax, Charlotte Alm, Stefan Annell

**Affiliations:** 1 Department of Psychology, Stockholm University, Stockholm, Sweden; 2 Department of Leadership and Command & Control, Swedish Defence University, Stockholm, Sweden

**Keywords:** insider threat, military personnel, military selection, personnel security, psychological assessments, risk prediction

## Abstract

Insider threat actions are often linked to individual psychological characteristics, yet the predictive utility of assessing such characteristics within operational personnel security frameworks remains unclear. The present study examined whether demographic and psychological assessment variables from the Swedish military selection process were associated with subsequent personnel security investigations resulting in adjudicative decisions within the Swedish Armed Forces. Data linked selection assessments conducted between 2010 and 2017 to 650 adjudicated investigation cases recorded between 2010 and 2023. Psychological measures included cognitive ability scores and interview-based ratings of psychological functioning and suitability for command. Separate logistic regression analyses compared two control samples with cases involving pre-existing antecedents, which were present at the time of assessment, and emergent antecedents, which arose later during service. Age at assessment and sex showed the strongest association with adjudicated investigations, whereas the psychological assessments showed weaker and less stable associations. Multivariate models demonstrated modest explanatory power, with pseudo-*R*^2^ values ranging from approximately 0.06 to 0.16. Broad psychological assessments thus demonstrated limited utility for distinguishing adjudicated investigations from controls, and their potential value as supplementary tools in highly sensitive contexts requires prospective evaluation and validation against clearly defined outcomes.

## 1 Introduction

Insider threat refers to the risk of intentional, negligent, or inadvertent misuse of privileged access in ways that harm the organization (Cybersecurity and Infrastructure Security Agency, 2020; Gelles, 2016; National Protective Security Authority, 2023). It is a complex and multifaceted phenomenon affecting a broad range of sectors, including private corporations, government agencies, and critical infrastructure, with consequences ranging from financial loss and theft of proprietary information to reputational harm and, in extreme cases, threats to national security. Insider threat is especially relevant in the current European security environment, particularly in the Baltic Sea region, where sabotage of critical infrastructure, intelligence activities, and hybrid warfare are growing concerns (Swedish Armed Forces, 2026; Swedish Security Service, 2026). Addressing these threats requires evidence-based personnel security practices, supported by valid and reliable methods of risk assessment. Such practices include preventive measures, for instance pre-employment vetting, and reactive measures, such as personnel security investigations, initiated when emerging concerns are detected.

Personnel security frameworks aim to counter insider threats, including insider fraud, sabotage, and espionage, by identifying factors believed to predict harmful outcomes. These factors, hereafter referred to as *antecedents*, may include both distal demographic and psychological characteristics, as well as more proximal behavioral and contextual conditions. If left unaddressed, antecedents may contribute to the progression toward insider threat outcomes. Previous research has identified psychological factors, including mental health issues, maladaptive personality traits, and behavioral patterns, such as security violations, absenteeism, and interpersonal abuse, as potential antecedents of insider threat actions (e.g., Baweja et al., 2019; Liang et al., 2016; Nurse et al., 2014; Shechter and Lang, 2011). Among the maladaptive personality traits discussed in this literature are the Dark Triad traits of narcissism, psychopathy, and Machiavellianism, which are associated with low empathy, manipulativeness, egocentrism, and hostility (Jakobwitz and Egan, 2006; Paulhus and Williams, 2002).

Demographic characteristics, including age and sex, have also been examined as potential distal antecedents, although reported patterns vary across types of insider threat actions and organizational settings. Studies of espionage, for example, have reported that middle-aged men are overrepresented among identified perpetrators (e.g., Herbig, 2017; Jonsson and Gustafsson, 2022; Lindskog et al., 2026), while different patterns have been observed in the financial sector. One study of insider cyber incidents found that 58% of offenders were male, with ages ranging from 18 to 58 years (Randazzo et al., 2005), whereas a study of insider fraud found that 69% of identified offenders were female (Cummings et al., 2012). These descriptive distributions should be interpreted cautiously however, as they may reflect workforce composition and differences in exposure or detection rather than differences in underlying risk.

Despite these reported associations, the predictive value of psychological and demographic factors in operational personnel security contexts remains uncertain. Existing research is largely based on descriptive case analyses of confirmed insider threat incidents and although these studies identify recurring characteristics among known perpetrators, they generally do not quantify their causal effects on harmful outcomes (e.g., Cummings et al., 2012; Herbig, 2017; Randazzo et al., 2005). Consequently, it remains unclear whether information routinely collected during personnel selection, including demographic characteristics and psychological assessment results, has predictive value when applied in practice. In particular, little is known about whether such information predicts subsequent organizational responses to identified security concerns, including personnel security investigations. These investigations are intended to assess and mitigate insider threat risk before harmful outcomes occur. As such, they have substantial implications not only for organizational security and staffing, but also for the employment and career trajectories of the individuals subjected to them. The limited empirical evidence concerning risk-mitigative measures therefore represents an important research gap.

The current study addresses this gap using declassified registry data from personnel security investigations within the Swedish Armed Forces, linked with psychological and demographic data from Swedish pre-enlistment selection assessments. It focuses on individuals who volunteered for basic military training between 2010 and 2017, when national military conscription was temporarily suspended. Unlike previous research examining confirmed insider threat actions, the present study concerns operational risk-mitigative measures and the pre-existing or emergent antecedents documented in connection with them. Pre-existing antecedents were retrospectively determined to have been present at the time of selection and security screening, but had either been undetected or had not prompted sufficient intervention. Emergent antecedents developed later during service or employment and could not have been observed at the initial assessment.

The study aimed to examine whether demographic characteristics and psychological assessment variables were associated with the odds of undergoing a personnel security investigation that resulted in an adjudicative decision concerning continued access. In this study, *antecedent* refers exclusively to a security-relevant behavior, event, or circumstance documented in connection with an adjudicated personnel security investigation. Demographic characteristics and psychological assessments are instead referred to as predictor variables. Although these predictors may theoretically represent distal antecedents, they were not labeled as such in the present study, thereby maintaining a clear distinction between predictors and the antecedents documented during the investigations.

Accordingly, the study addresses two research questions:

1. Which demographic and psychological predictors are associated with adjudicated personnel security investigation involving pre-existing or emergent insider threat antecedents, respectively?
2. If used in combination, to what extent can demographic and psychological predictors predict adjudicated personnel security investigation involving pre-existing or emergent insider threat antecedents, respectively?

## 2 Method

### 2.1 Setting

The Swedish Armed Forces comprise a wide range of security-classified and non-classified positions across several categories of service. These include continuously and periodically serving officers, soldiers, and sailors, civilian employees, and Home Guard volunteers (Försvarsmakten, 2021). Between 2010 and 2017, basic military training was voluntary. Applicants completed physical, medical, cognitive, and psychological evaluations administered by the Swedish Defense Conscription and Assessment Agency (Ludvigsson et al., 2022). Eligible recruits were then subject to basic security screening assessing loyalty, reliability, and vulnerability from a personnel security perspective, followed by one of two records checks procedures (Försvarsmakten, 2021).

Applicants for security-classified positions received a full records check by the Swedish Security Service covering criminal and non-criminal registers. Importantly, immaterial findings were excluded. Applicants for non-classified positions were instead subject to a limited criminal record extract covering convictions and suspected offences during the preceding 5 years. These extracts could reveal minor or suspected criminality typically excluded from full records checks, but contained no information from non-criminal registers. This created an asymmetry in antecedent detection between the two groups.

Individuals who accepted a security-classified position were thereafter subject to continuous monitoring through daily records checks and periodical follow-up security evaluations (Försvarsmakten, 2021). Subsequent security concerns could trigger a personnel security investigation. If pertinent insider threat antecedents were identified, the investigations resulted in an adjudicative decision regarding the individual’s continued access.

### 2.2 Design

This study analyzed declassified operational personnel security data from the Swedish Armed Forces, linked to data from military selection assessments. The datasets comprise data from the personnel security investigations conducted between 2010 and the end of 2023 among individuals who volunteered for basic military training between 2010 and 2017. Data were obtained from the Swedish Armed Forces in May 2025. The study employs a longitudinal design to examine whether psychological assessment variables from the military selection process, together with demographic characteristics (age and sex), are associated with the likelihood of subsequent risk-mitigative measures, operationalized as adjudicated personnel security investigations. The analysis further distinguishes between pre-existing and emergent antecedents identified in these investigations. To enable comparisons between individuals subjected to personnel security investigations and those who were not, three samples were included: an investigation sample and two control samples.

### 2.3 Ethical issues

The study was conducted in collaboration with the Swedish Armed Forces. To ensure anonymity and make the original, classified data accessible for research purposes, the Swedish Armed Forces aggregated and declassified the data. The study has been approved by the Swedish Ethical Review Authority (File number 2023-04464-01) and informed consent was not required in accordance with the Swedish ethical review authority.

### 2.4 Measures

The declassified data included information on the presence of identified pre-existing and emerging antecedents (outcome variable), as well as demographic characteristics and psychological assessment results from the military selection process (predictors). The predictors and their descriptive statistics are presented in Table 1.

**Table 1:** Descriptive statistics for demographics characteristics and psychological assessment variables per sample.

	Investigation sample		Non-matched control sample		Matched control sample	
	*M*	*SD*	*M*	*SD*	*M*	*SD*
Age at first assessment	22.11	4.46	21.00	3.41	21.07	3.89
Age category	*n*	%	*n*	%	*n*	%
18–21	403	62.00	482	74.20	493	75.80
22–25	157	24.20	118	18.20	105	16.20
26–30	56	8.60	28	4.30	28	4.30
31 and above	34	5.20	22	3.40	24	3.70
Sex	*n*	%	*n*	%	*n*	%
Male	615	95	554	85.20	550	84.60
Female	35	5	96	14.70	100	15.40
Category of service	*n*	%	*n*	%	*n*	%
Recruits	279	42.9	55	8.50	280	43.10
Consultants	2	0.30	7	1.10	2	0.30
Volunteers	21	3.20	33	5.10	21	3.20
Home guard	92	14.20	90	13.80	92	14.20
Civilian employees	10	1.50	10	1.50	9	1.40
Squad leaders, soldiers and sailors	214	32.90	355	54.60	214	32.90
Officers and specialist officers	32	4.90	100	15.40	32	4.90
Psychological assessments	*M*	*SD*	*M*	*SD*	*M*	*SD*
Cognitive ability	2.28	0.47	2.36	0.50	2.28	0.49
Psychological level of function	2.21	0.52	2.26	0.55	2.27	0.50
Suitability for military command	2.09	0.50	2.17	0.52	2.18	0.48

Cognitive ability, psychological level of function, and suitability for military command were aggregated from nine-point scales to three-point scales (1–3) and treated as continuous in the analyses. Higher scores indicate more favorable selection assessments. Because a minimum rating of four on the original assessment scales was required for eligibility for basic military training, and because the samples primarily comprised individuals in military positions requiring such training, relatively few individuals received scores corresponding to the lowest category on the aggregated three-point scale.

#### 2.4.1 Pre-existing and emergent antecedents

A pre-existing antecedent was an antecedent that was retrospectively determined to have been present on or before the initial selection and basic security screening. Although it already existed, it had either not been detected, disclosed, or considered sufficient to prompt intervention at that time. An emergent antecedent first developed after the initial selection and basic security screening, during subsequent military service or civilian employment. It was not considered present at the initial assessment and therefore could not have been identified through the selection or screening procedures.

The Swedish Armed Forces assigned each individual to one group based on the recorded timing of the principal event, circumstance, or behavior that prompted the personnel security investigation. If this principal factor was determined to have been present at or before the initial assessment and basic security screening, the case was classified as pre-existing. If it first occurred after assessment, the case was classified as emergent. Because of anonymization requirements, the declassified dataset did not indicate whether individuals in the pre-existing group also had emergent antecedents, and vice versa. Consequently, individuals could not be assigned to both groups or to a separate mixed category.

Antecedents were categorized as either criminal behaviors, ranging from minor infractions to serious criminal conduct, or non-criminal risk domains, encompassing a broad range of person-related and situational circumstances considered relevant to insider threat. A substantial majority of the investigated individuals exhibited one or more criminal antecedents (72%). Non-criminal antecedents or mixed patterns were less frequent, observed in 13 and 15% of cases, respectively. The most prevalent forms of criminal behavior involved narcotics-related and violent offenses, followed by alcohol- and theft-related crimes, although less frequent categories such as sexual and fraud-related offenses were also represented.

Non-criminal person-related antecedents were comparatively rare and included, for example, psychological concerns such as mental health problems and maladaptive personality traits, as well as security negligence and risk-prone behaviors, including excessive alcohol consumption or financial irresponsibility. Situational antecedents, such as extremist affiliations, criminal associations, financial instability, and foreign connections, were identified in a smaller subset of cases.

#### 2.4.2 Demographic predictors

Demographic variables included sex and age at assessment. Age at assessment was treated as a categorical variable, grouping individuals according to their age at the time of their first completed military selection assessment, into four intervals: 18–21, 22–25, 26–30, and 31 years or older. This grouping was done by the Swedish Armed Forces for anonymization purposes. The reference category was 18–21 years, representing the primary target group for military service.

Sex was coded as a binary variable, derived from the individual’s personal identity number, which indicates sex assigned at birth. The reference category was set to male, as men constituted the majority of the samples.

#### 2.4.3 Psychological assessment predictors

Cognitive ability was measured using the SEB 2000 (Swedish Enlistment Battery), providing an overall measure of general intelligence (g) on a stanine scale, ranging from one to nine with a mean of five (Ludvigsson et al., 2022). The SEB included 10 subtests, covering verbal ability, spatial ability, inductive ability, and technical comprehension.

Psychological level of function is an evaluation of general suitability for military service on a nine-point scale, assessing traits such as motivation, social skills, extroversion, emotional stability, independence and initiative, as well as other factors related to service-related stress resilience (Ludvigsson et al., 2022).

Suitability for military command assessed aptitude for leadership positions on a nine-point scale, evaluating traits such as adaptability, dependability, and responsibility across different contexts. Only applicants with a stanine score of at least five on the cognitive ability test were assessed on their suitability for command positions.

To reduce the risk of re-identification, the Swedish Armed Forces aggregated the original nine-point scales into three-point scales; low (1–3), medium (4–6), and high (7–9). This transformation preserved the ordinal nature of the variables while reducing data granularity to ensure anonymity. The original measures are assumed to reflect underlying continuous constructs. This assumption was extended to the aggregated three-point scales, which were therefore treated as continuous in the analyses. The robustness of this assumption was examined through supplementary analyses treating the variables as categorical, which yielded similar results (see Supplementary Tables).

Many subjects have undergone multiple assessments, some applying as many as four times. For all three samples, the first complete assessment was used in the analyses, regardless of any subsequent psychological assessment results.

### 2.5 Sample

All individuals applied for voluntary basic military training between July 1, 2010, and July 31, 2017, and underwent, either partially or fully, the military selection assessment at the Swedish Defense Conscription and Assessment Agency. Importantly, not all individuals in the sample were deemed eligible for basic military training, yet still passed a basic security screening.

Applicants eligible for basic military training were screened as recruits, either for security-classified or non-classified positions. Recruits in non-classified positions were subject only to criminal record extracts, resulting in systematic differences in the types of detectable antecedents across cohorts. Because this subgroup could not be isolated in the dataset, it was not possible to examine outcome differences between the two approaches.

Ineligible applicants, as well as eligible applicants who deferred enlistment, underwent basic security screening at a later stage in connection with civilian employment or voluntary, non-professional military engagement. As a result, multiple pathways existed from assessment and screening into service or employment, explaining how individuals with relatively low assessment scores could still obtain security-classified positions.

#### 2.5.1 Investigation sample

The investigation sample (*n* = 650) comprised all members of the voluntary cohort who were included in the Swedish Armed Forces personnel security registry. These individuals (a) were assessed by the Swedish Defence Conscription and Assessment Agency between July 1, 2010, and July 31, 2017, (b) passed basic security screening as eligible recruits or later as volunteers, consultants and employees placed in security-classified positions, (c) were subject to full records checks or criminal records extracts, and (d) subsequently underwent a personnel security investigation that (e) identified insider threat antecedents considered sufficiently relevant to warrant adjudication. Distribution of sex, age at assessment, and categories of service within the investigation sample is detailed in Table 1.

The investigation sample comprises two subgroups: a pre-existing antecedent group (*n* = 289) and an emergent antecedent group (*n* = 361). According to supplementary aggregate statistics provided by the Swedish Armed Forces, the recorded onset for pre-existing antecedents were approximately 3 years prior to assessment (*M* = 3.08, S*D* = 2.34), with an age of onset ranging from 15 to 38 years (*M* = 19.79, *SD* = 4.00). In contrast, antecedents in the emergent group emerged, on average, approximately 4 years after assessment (*M* = 4.27, *SD* = 2.69), with age at onset ranging from 19 to 54 years (*M* = 25.78, *SD* = 5.12). These summary statistics were supplied separately by the Swedish Armed Forces.

#### 2.5.2 Control samples

Two control samples, each comprising 650 individuals, were drawn from the 15,846 voluntary applicants placed in security-classified positions within the Swedish Armed Forces and were not included in the personnel security registry. The non-matched control sample was selected at random without regard to category of service, whereas the matched control sample was randomly sampled within strata to reproduce the category of service distribution of the investigation sample. Individuals in the control samples may have undergone personnel security investigations. However, because they were not included in the personnel security registry, any identified antecedents were, by definition, not deemed sufficiently relevant to warrant adjudication.

The non-matched control sample was drawn from the overall population of individuals placed in security-classified positions and was comparable to that population in terms of category of service. However, because it excluded recruits who passed basic security screening but were never assigned to a security-classified position, it was not representative of the broader population screened for both security-classified and non-security-classified positions.

A Pearson’s chi-squared test was used to compare the distribution of category of service in the investigation sample with that of the non-matched control sample. The test showed that recruits were significantly overrepresented in the investigation sample (standardized residual = 14.22, *p* < 0.001), whereas squad leaders, soldiers and sailors (standardized residual = −7.88*, p* < 0.001), as well as officers and specialist officers (standardized residual = −6.24, *p* < 0.001), were significantly underrepresented. The overrepresentation of recruits is consistent with the exclusion of non-classified recruits from the non-matched control sample. At the same time, assuming that the non-matched sample was representative of individuals placed in security-classified positions, the observed differences indicate that the investigation sample was not fully representative of the broader security-classified population with respect to category of service. See Supplementary Tables for full results.

To remedy this imbalance, a matched control sample was included. This sample was randomly selected and stratified to match the pre-existing and emergent subgroups on category of service (e.g., officers, civilians, and recruits). This procedure helped control for potential confounding associated with category of service, including systematic differences in cognitive ability requirements and suitability for military command ratings across categories. The matched control sample replicated the structure of the investigation sample, comprising a pre-existing subgroup (*n* = 289) and an emergent subgroup (*n* = 361).

For individuals in the control samples, category of service was determined based on the highest position attained as of 2023, when the data were extracted. As many individuals served in multiple roles over time, a hierarchical classification was applied, assigning each individual to their highest-ranking position according to the following hierarchy: (1) recruits, (2) consultants, (3) volunteers in the Swedish Armed Forces voluntary organizations, (4) Home Guard volunteers, (5) civilian employees, (6) squad leaders, soldiers and sailors, and (7) officers. Distribution of sex, age at assessment, and categories of service for both control samples is described in Table 1.

### 2.6 Analysis

Because all unique combinations of variables were removed in the combined datasets to prevent identification (e.g., profiles that could potentially identify individuals), some data loss occurred in datasets containing multiple predictors. To minimize this loss, the predictive modelling process was conducted in two phases.

In the first phase, exploratory analyses were conducted using six separate single-predictor datasets, each containing either a demographic or a psychological assessment variable. The purpose of these analyses was to identify candidate predictors that were sufficiently relevant to justify inclusion in subsequent combined datasets, despite the trade-off of reduced sample size.

The binary outcome represented adjudicated personnel security investigation status (investigated and adjudicated = 1, control = 0). Accordingly, bivariate logistic regression models were used to estimate the odds of undergoing a personnel security investigation that resulted in an adjudicative decision, compared with controls who had not undergone an adjudicated investigation. Separate analyses were conducted for adjudicated cases that identified pre-existing and emergent antecedents, respectively. Odds ratios (OR) with 95% confidence intervals (CI) were calculated to estimate the strength and direction of these associations. Missing data patterns are summarized in Table 2.

**Table 2:** Sample sizes and missing data across variables and datasets.

Dataset	Investigation sample		Non-matched control sample		Matched control sample	
	*n*	Missing	*n*	Missing	*n*	Missing
Age at assessment	650	0	650	0	650	0
Sex	650	0	650	0	650	0
Cognitive ability	646	4	643	7	636	14
Psychological level of function	633	17	630	20	626	24
Suitability for military command	520	130	537	113	534	116
Combined datasets						
Dataset 1	611	39	618	32	598	52
Dataset 2	505	145	521	129	500	150

Bivariate datasets for age at assessment and sex included all 1,950 individuals. Suitability for military command exhibited the highest level of missing data, as it was only assessed for individuals with a cognitive ability score of five or above. Compared to the bivariate datasets, combined datasets showed a modest additional reduction in sample size. For the first dataset, including psychological level of function, this reduction ranged from approximately 2 to 5% across samples. For the second dataset including suitability for military command, it ranged from approximately 3 to 6%. These reductions reflect the combined effects of missing data and anonymization procedures.

In the second phase of data collection and analysis, the authors requested two datasets that combined multiple predictors selected on the basis of the single-predictor analyses:

1. Combined dataset 1: Sex, age at first assessment, cognitive ability, and psychological level of function.
2. Combined dataset 2: Sex, age at first assessment, cognitive ability (with a stanine score of at least five), and suitability of military command.

The Swedish Armed Forces subsequently merged the requested variables into combined datasets. To ensure anonymity, cases with unique combinations of variables were removed, resulting in reduced sample sizes (see Table 2).

Separate logistic regression models examined the associations of demographic and psychological assessment variables with adjudicated personnel security investigations involving pre-existing and emergent antecedents. All predictor variables were entered simultaneously into the model. Results are reported as OR with 95% CI. Overall predictive power was assessed using Nagelkerke’s pseudo-*R*^2^ and the Chi-square test.

All statistical analyses were conducted using R version 4.5.0 (R Core Team, 2025) in combination with packages *tidyverse* (Wickham et al., 2019) for data manipulation and visualization and *broom* (Robinson et al., 2025) for tidying model outputs. Reporting was supported using the packages *quarto* (Allaire and Dervieux, 2025), *knitr* (Xie, 2025), *kableExtra* (Zhu, 2024), and *glue* (Hester and Bryan, 2024). The *polycor* package (Fox, 2022) was used to compute polychoric and polyserial correlations. All datasets, the codebook, and code required to reproduce the reported results are available in the Supplementary material.

#### 2.6.1 Data requirements for performing logistic regression analyses

To assess whether the data were suitable for multivariate analyses, correlations among predictors were examined across all three samples for each of the two combined datasets. Maximum-likelihood (ML) heterogeneous correlations were computed, combining Pearson, polychoric, and polyserial estimates as appropriate for variable scale. Intercorrelations among predictors were generally weak, with most below 0.12 and the largest, between sex and cognitive ability, at −0.23. Cognitive ability was weakly but consistently correlated with psychological level of function and suitability for military command, with correlations ranging from 0.10 to 0.15. Overall, the correlation and diagnostic patterns supported the inclusion of all predictors in subsequent regression models. Full correlation matrices are available in the Supplementary Tables.

## 3 Results

### 3.1 Bivariate associations with adjudicated personnel security investigations

Bivariate results are presented in Table 3. All models performed significantly better than the corresponding null models. Full bivariate results from the bivariate datasets are provided in the Supplementary Tables.

**Table 3:** Bivariate logistic regression analyses predicting preexisting and emergent antecedents.

Bivariate analyses	Non-matched control sample		Matched control sample	
	Existing OR [95% CI]	Emergent OR [95% CI]	Existing OR [95% CI]	Emergent OR [95% CI]
Demographics				
Age at assessment (Reference = 18–21)	*n* = 939	*n* = 1,011	*n* = 578	*n* = 722
22–25 vs. 18–21	2.42*** [1.73, 3.37]	1.12 [0.8, 1.55]	3.11*** [2.05, 4.77]	1.18 [0.8, 1.73]
26–30 vs. 18–21	4.22*** [2.5, 7.19]	1.34 [0.73, 2.42]	5.53*** [2.76, 12.09]	1.16 [0.6, 2.26]
31 + vs. 18–21	2.83** [1.48, 5.38]	1.28 [0.64, 2.5]	2.92** [1.35, 6.71]	1.12 [0.53, 2.39]
Nagelkerke *R*^2^	0.075***	0.002	0.112***	0.002
Sex (Reference = Male)	*n* = 939	*n* = 1,011	*n* = 578	*n* = 722
Woman vs. Male	0.25*** [0.13, 0.45]	0.39*** [0.24, 0.62]	0.30*** [0.14, 0.56]	0.32*** [0.19, 0.52]
Nagelkerke *R*^2^	0.039***	0.023***	0.033***	0.040***
Military selection assessment				
Cognitive ability	*n* = 929	*n* = 1,003	*n* = 567	*n* = 715
Per one-unit increase (1–3)	0.70* [0.52, 0.93]	0.72* [0.55, 0.94]	0.98 [0.69, 1.39]	1.01 [0.75, 1.36]
Nagelkerke *R*^2^	0.009*	0.008*	0	0
Psychological level of function	*n* = 913	*n* = 980	*n* = 561	*n* = 698
Per one-unit increase (1–3)	0.81 [0.62, 1.06]	0.92 [0.72, 1.18]	0.78 [0.56, 1.1]	0.82 [0.62, 1.09]
Nagelkerke *R*^2^	0.004	0.001	0.005	0.003
Suitability for military command	*n* = 764	*n* = 830	*n* = 458	*n* = 596
Per one-unit increase (1–3)	0.73* [0.54, 0.98]	0.75* [0.56, 0.99]	0.66* [0.45, 0.97]	0.70* [0.5, 0.97]
Nagelkerke *R*^2^	0.008*	0.007*	0.013*	0.010*

Due to missing values, the number of subjects in each bivariate dataset varies. The suitability for military command data includes fewer subjects than the other predictors, as only individuals with a cognitive ability score of five or above on the original stanine scale received a suitability for military command rating. *p < 0.05, **p < 0.01, ***p < 0.001.

#### 3.1.1 Demographic variables

Age at assessment was strongly associated with adjudicated investigations involving pre-existing antecedents in both control comparisons. Compared with the reference group (18–21), all older age groups had significantly higher odds of such investigations. The strongest association was observed among those aged 26–30 years, who had 322% higher odds (OR = 4.22) in the non-matched comparison and 453% higher odds (OR = 5.53) in the matched comparison.

Sex was strongly associated with adjudicated investigations involving both pre-existing and emergent antecedents. In the non-matched comparisons, women had 75% lower odds (OR = 0.25) of an investigation involving pre-existing antecedents and 61% (OR = 0.39) lower odds of one involving emergent antecedents than men. In the matched comparisons, the corresponding odds were 70% (OR = 0.30) and 68% (OR = 0.32) lower.

#### 3.1.2 Psychological assessment variables

Suitability for military command was significantly associated with adjudicated investigations status in all comparisons. In the non-matched comparisons, each one-point increase on the three-point scale was associated with 27% lower odds (OR = 0.73) of an investigation involving pre-existing antecedents and 25% lower odds (OR = 0.75) of one involving emergent antecedents. In the matched comparisons, the corresponding odds were 34% (OR = 0.66) and 30% (OR = 0.70) lower.

Cognitive ability was significantly associated with adjudicated investigation status only in the non-matched comparisons. Each one-point increase was associated with 30% lower odds (OR = 0.70) of an investigation involving pre-existing antecedents and 28% lower odds (OR = 0.72) of one involving emergent antecedents. Cognitive ability was not significantly associated with either investigation subgroup in the matched comparisons. Psychological level of function was not significantly associated with either investigation subgroup in any comparison.

### 3.2 Multivariate associations with adjudicated personnel security investigations

Multivariate results are presented in Table 4. All models performed significantly better than the corresponding null models. Nagelkerke’s *R*^2^ values ranged from 0.043 to 0.149 in Dataset 1 and from 0.042 to 0.166 in Dataset 2, with greater explanatory power for pre-existing than for emergent antecedents. Full bivariate results from the combined datasets are provided in the Supplementary Table.

**Table 4:** Multiple logistic regression models using two combined datasets: Dataset 1 (including psychological level of function) and Dataset 2 (including suitability for military command).

Dataset 1	Non-matched control sample		Matched control sample	
	Existing (*n* = 888)	Emergent (*n* = 959)	Existing (*n* = 534)	Emergent (*n* = 675)
Predictor	OR [95% CI]	OR [95% CI]	OR [95% CI]	OR [95% CI]
Age: 18–21 (Ref)	–	–	–	–
Age: 22–25	2.49*** [1.76, 3.54]	1.15 [0.81, 1.63]	2.90*** [1.89, 4.51]	1.27 [0.85, 1.92]
Age: 26–30	4.3*** [2.42, 7.75]	1.27 [0.65, 2.44]	5.64*** [2.61, 13.64]	1.38 [0.64, 3]
Age: 31+	1.39 [0.63, 2.93]	1.05 [0.49, 2.16]	3.85* [1.28, 13.39]	1.67 [0.67, 4.34]
Sex (Woman)	0.16*** [0.07, 0.33]	0.28*** [0.16, 0.49]	0.20*** [0.08, 0.46]	0.22*** [0.11, 0.38]
Cognitive ability	0.56*** [0.4, 0.77]	0.69* [0.51, 0.92]	0.63* [0.42, 0.95]	0.91 [0.65, 1.28]
Psychological level of function	0.87 [0.65, 1.16]	0.96 [0.74, 1.24]	0.84 [0.57, 1.23]	0.86 [0.63, 1.17]
Nagelkerke *R*^2^	0.132***	0.043***	0.149***	0.065***

Dataset 2	Non-matched control sample		Matched control sample	
	Existing (*n* = 739)	Emergent (*n* = 808)	Existing (*n* = 435)	Emergent (*n* = 570)
Predictor	OR [95% CI]	OR [95% CI]	OR [95% CI]	OR [95% CI]
Age: 18–21 (Ref)	–	–	–	–
Age: 22–25	2.49*** [1.69, 3.68]	1.18 [0.81, 1.72]	2.58*** [1.61, 4.17]	1.23 [0.8, 1.92]
Age: 26–30	5.03*** [2.71, 9.53]	1.43 [0.69, 2.88]	5.19*** [2.36, 12.69]	1.59 [0.69, 3.73]
Age: 31+	1.86 [0.71, 4.58]	1.92 [0.84, 4.37]	–	7.77** [2.00, 51.74]
Sex (Woman)	0.17*** [0.07, 0.38]	0.37*** [0.20, 0.64]	0.24** [0.08, 0.59]	0.25*** [0.13, 0.46]
Cognitive ability	0.59** [0.42, 0.84]	0.68* [0.50, 0.92]	0.73 [0.47, 1.11]	0.99 [0.69, 1.43]
Suitability for military command	0.74 [0.53, 1.04]	0.76 [0.56, 1.03]	0.63* [0.4, 0.97]	0.7 [0.48, 1.00]
Nagelkerke *R*^2^	0.134***	0.042***	0.166***	0.080***

Model significance relative to the null (intercept-only) model was assessed using chi-square tests. The overall predictive power of the models were evaluated using Nagelkerke’s pseudo-*R*^2^. Non-matched comparisons used the full non-matched control sample, whereas matched comparisons used comparably sized matched control samples. Ref = reference category.

*p < 0.05, **p < 0.01, ***p < 0.001.

#### 3.2.1 Combined dataset 1: age at assessment, sex, cognitive ability, and psychological level of function

Two multivariate findings differed from the corresponding bivariate results. In the matched comparison, cognitive ability became significantly associated with adjudicated investigations involving pre-existing antecedents after adjusting for age at assessment, sex, and psychological level of function. Each one-point increase was associated with 37% lower odds (OR = 0.63) of these investigations. In the non-matched comparison, the association between the 31 + age group and investigations involving pre-existing antecedents was no longer statistically significant.

#### 3.2.2 Combined dataset 2: age at assessment, sex, cognitive ability, and suitability for military command

Several multivariate findings differed from the corresponding bivariate results. As in Dataset 2, the associations between the 31 + age group and investigations involving pre-existing antecedents were no longer statistically significant in the non-matched comparison. In the matched comparison, however, individuals aged 31 years or older had significantly higher odds (OR = 7.77) of an investigation involving emergent antecedents than those aged 18–21 years.

After adjustment for the other predictors, suitability for military command was significantly associated with investigations involving pre-existing antecedents only in the matched comparison. Its association with investigations involving emergent antecedents in the matched comparison did not reach statistical significance (*p* = 0.053). No significant association with either investigation subgroup was found in the non-matched models.

## 4 Discussion

The study found weak associations between psychological factors, measured through broad military selection assessments, and adjudicated personnel security investigations involving pre-existing or emergent antecedents. Age at assessment was strongly associated with investigations involving pre-existing antecedents, with older applicants having higher odds of these investigations. By contrast, age was not significantly associated with investigations involving emergent antecedents except in one analysis. Sex was consistently associated with adjudicated investigation status across all comparisons, with men having substantially higher odds of investigations involving both pre-existing and emergent antecedents than women.

Cognitive ability emerged as the most consistent psychological assessment factor, but only in the non-matched analyses. Higher cognitive ability scores were associated with significantly lower odds of investigations involving both pre-existing and emergent antecedents. However, this protective effect was absent in the matched comparisons. Suitability for military command demonstrated weaker and more variable associations. Lower ratings were associated with higher odds of investigations involving both types of antecedents, but only one of these associations remained statistically significant in the combined models. Psychological level of function was not associated with either investigation subgroup.

### 4.1 Age at assessment: age as time at risk

Age was measured at the initial assessment and does not represent age at antecedent onset or adjudication. This is particularly important for emergent antecedents, which arose an average of more than 4 years after assessment. As such, age at assessment is a weak proxy for age at antecedent onset and associations involving emergent antecedents should be interpreted cautiously.

The association between older age at assessment and adjudicated investigations involving pre-existing antecedents is likely attributable to longer lifetime exposure in the general population prior to enlistment; older applicants have simply had more time and opportunity for such antecedents to manifest before enlistment. Alternatively, age at assessment may reflect life-course differences. While many older applicants may undoubtedly possess constructive motives for seeking military service, such as a sense of purpose, meaning, or genuine interest, entering military service later in life may also indicate atypical life trajectories, potentially linked to prior instability or difficulties in civilian life. From this perspective, age at assessment may function less as a causal factor than as a proxy for accumulated exposure to risk-adjacent experiences.

Although the operational use of age as a screening variable may be restricted in some jurisdictions due to legal and ethical considerations, the finding remains potentially valuable from both theoretical and practical perspectives. Theoretically, it emphasizes the distinction between risk exposure and risk propensity, suggesting that demographic variables such as age at assessment may reflect opportunity rather than underlying psychological characteristics. Practically, the finding support thorough backgrounds checks across age groups but does not justify treating age itself as an indicator of ongoing personnel security risk.

### 4.2 Sex: maleness as a consistent but complex predictor

The strong association between sex and the identification of both pre-existing and emergent antecedents is not unexpected. Men are consistently overrepresented in insider threat research (e.g., Herbig, 2017; National Protective Security Authority, 2013; Randazzo et al., 2005). Because the control samples reflected the sex composition of the source population, the observed association cannot be attributed solely to the predominance of men in security-classified positions. However, the underlying mechanisms remain unclear and the study cannot establish that men have a greater underlying propensity to exhibit insider threat antecedents.

The associations could reflect sex differences in the prevalence or type of antecedents, their detectability, or the likelihood of investigation and adjudication. For example, men may be more likely to engage in behaviors that attract scrutiny, such as aggression (Björkqvist, 2018), risk-taking (Byrnes et al., 1999), or substance use (Alm et al., 2010; Nolen-Hoeksema, 2004). This explanation remains theoretical, as the present study did not directly examine detection or investigation processes. Future research should therefore determine whether antecedent prevalence, detection, investigation, and adjudication differ by sex, including whether less readily observable antecedents are undetected among women. Regardless of the underlying mechanism, sex may not be a permissible factor in operational personnel security frameworks due to legal and ethical concerns regarding discrimination.

### 4.3 Cognitive ability: intelligence as a protective factor

The finding that cognitive ability was only significant in the non-matched comparisons, and not in the comparison that controlled for category of service, likely reflects the influence of category of service as a mediating factor. Different categories vary in terms of cognitive ability thresholds; for instance, the Home Guard requires lower cognitive ability scores than career officers and specialist officers, which cause restriction of range in the data. Matching on the type of service may therefore have obscured the relationship between cognitive ability and the odds of investigation. Unfortunately, category of service was not available as a variable in the combined datasets, which precluded further examination of its influence on the outcome.

In the non-matched analyses, cognitive ability may exert both direct and indirect effects on insider threat antecedents. Past research has shown that higher general intelligence (g) predicts stronger reasoning, problem-solving, and decision-making abilities, which directly enhance the individuals’ capacity to cope with everyday demands (Gottfredson, 1997). Indirectly, *g* is associated with higher academic performance (Rosander et al., 2011; Kuncel et al., 2010) and greater socioeconomic success (Sorjonen et al., 2012; Strenze, 2007). Although *g* has not been found to generally predict counterproductive work behaviors, it has been positively linked to organizational citizenship behaviors (Gonzalez-Mulé et al., 2014). Collectively, these findings suggest that individuals with higher cognitive ability are generally better equipped to manage personal and professional challenges constructively, thereby reducing their potential exposure to insider threat antecedents such as financial issues and substance misuse. This interpretation aligns with research showing that lower intelligence predicts criminal offending, the most frequent antecedent identified in the personnel security registry data (Frisell et al., 2012; Ttofi et al., 2016).

The finding that cognitive ability was associated with investigations involving pre-existing antecedents only in the matched control sample, and only when demographic predictors were controlled for, remains somewhat unclear. Several explanations are possible. One is that this pattern reflects a partial suppression effect, whereby controlling for demographic variables removes shared variance from the measures of cognitive ability and allows the unique contribution of cognitive ability to emerge more clearly. Alternatively, because cognitive ability tends to be measured with relatively high reliability and was found in the correlation analyses to be weakly, but significantly, associated with demographic characteristics, controlling for the demographic variables may have added some shared and relevant variance from the demographic variables, thus elevating the estimate of risk associated with cognitive ability.

### 4.4 Psychological level of function: challenging the psychological hegemony

Psychological level of function is intended as a broad measure of overall suitability for military service, incorporating traits such as emotional stability, social skills, and stress resilience. Although insider threat research has identified characteristics such as impulsivity, immaturity, and low self-esteem as potential antecedents (e.g., National Protective Security Authority, 2013), traits likely to be reflected in lower psychological level of function ratings, the measure was not associated with the likelihood of subsequent risk-mitigative measures in the present study.

Several explanations warrant consideration. First, the construct underlying the psychological level of function assessment may be too broad to adequately capture insider threat-specific antecedents. Rather than focusing on traits more directly associated with insider threat, such as antisocial or grandiose tendencies (Maasberg et al., 2015), impulsivity (National Protective Security Authority, 2013), or low conscientiousness (McCormac et al., 2017), the assessment represents a composite measure encompassing a broad range of characteristics related to general psychological adjustment and suitability for military service. Consequently, the measure may lack the specificity necessary to differentiate individuals at higher versus lower risk of risk-mitigative measures. This interpretation is partly supported by the findings for suitability for military command, which, although also broad in scope, may better capture traits more directly relevant to insider threat, such as dependability and responsibility. Alternatively, as discussed previously, the absence of significant associations may also reflect substantial range restriction within the sample.

### 4.5 Suitability for military command: limited and inconsistent effects

The variability in the associations between suitability for military command and adjudicated investigations across the bivariate and multivariate analyses warrants further consideration. Although suitability for military command is a broad and somewhat loosely defined construct, its underlying attributes, such as dependability, responsibility, and leadership potential, align closely with established psychological frameworks of conscientiousness and integrity. These traits have consistently been linked to less counterproductive work behaviors (Ones et al., 1993), higher job satisfaction (Judge et al., 2002) and greater organizational citizenship behavior (Borman et al., 2001). From a theoretical perspective, such characteristics could reasonably be expected to function as protective factors against insider threat antecedents, consistent with the pattern observed in the bivariate analyses.

The attenuation of these associations in the combined, multivariate datasets may partly reflect the reduced statistical power resulting from smaller samples sizes. Consistent with this explanation, suitability for military command was already non-significant in the bivariate analyses conducted using the combined datasets. However, it is also important to consider the substantial range restriction affecting this predictor, as only individuals with cognitive ability scores of five or higher received a rating. Consequently, the observed associations may partly reflect the restricted distribution. This also raises the possibility that the relationship between suitability for military command and adjudicated investigations is partly shaped by cognitive ability itself, rather than suitability for military command functioning as a fully independent psychological predictor. That said, the correlation between cognitive ability and suitability for military command was low (see Supplementary Tables).

It is also important to consider the potential for confounding in the assessment process. If pre-existing antecedents, such as a history of risk-taking or criminal behavior, were disclosed during evaluation, they would influence the psychologist’s suitability rating. In this case, lower ratings would reflect the presence of pre-existing antecedents rather than predict them, artificially inflating association. However, because such pre-existing antecedents would also be expected to influence overall ratings of psychological functioning, and psychological level of function was not correlated with insider antecedents, this explanation appears less likely. Instead, the findings may highlight the conceptual overlap between suitability for military command ratings and other related constructs, such as conscientiousness and integrity, lending support to the theoretical emphasis on psychological characteristics and the potential utility of personality-based assessments within preventive frameworks.

### 4.6 Interpreting the models: investigation, concealment, and criminality

The models estimate the odds of belonging to the investigation sample, that is, of undergoing a personnel security investigation in which antecedents considered sufficiently relevant to warrant adjudication were identified. They do not directly estimate the odds of exhibiting pre-existing or emergent antecedent.

Furthermore, nearly half of the investigation sample had pre-existing antecedents that were either not identified during selection or were insufficiently dealt with at that time. This may indicate concealment through deception or omission, although incomplete records and limitations in the initial screening process cannot be excluded. Consequently, the analyses of pre-existing antecedents may partly capture a propensity to withhold unfavorable information in high-stakes settings, rather than the presence of antecedents alone.

Additionally, because criminal antecedents predominated in the dataset, the models may primarily be predicting criminal behaviors warranting adjudication, rather than personnel security concerns more broadly. Although criminality is a relevant proximal antecedent to insider threat actions and may reflect poor judgement or disregard for rules and norms, it represents only one element in a broader constellation of individual and situational risk factors. Insider threat actions are likely shaped by complex interactions among these factors rather than by criminality alone.

Together, the definition of the outcome, the differing interpretation of pre-existing antecedent cases, and the predominance of criminal antecedents affect the generalizability and practical utility of the models. These factors should be considered when applying the findings in operational contexts, particularly when the objective is to identify personnel security risks other than criminal conduct.

### 4.7 Limitations

The study’s unique design and datasets entailed several limitations affecting the generalizability and practical utility of its findings. Accordingly, both significant and non-significant results should be interpreted cautiously. Importantly, multiple selection processes restricted the range of predictors. Most individuals had met the minimum requirements for military service and passed basic security screening, while suitability for military command was only assessed among individuals scoring five or higher on the original nine-point cognitive ability scale. Consequently, variability within the samples was constrained, potentially influencing observed associations in ways that cannot be fully evaluated.

Data accessibility and granularity imposed further constraints. To minimize the risk of re-identification the authors only received aggregated, declassified data. The original nine-point assessment scales were also reduced to three-point scales, further restricting variability and potentially obscuring detectable associations.

Moreover, the models assume that the psychological assessments conducted by the Swedish Defense Conscription and Assessment Agency are valid and reliable. However, the assessment procedures, targeted constructs, and psychometric properties are not publicly documented, limiting evaluation of their research utility. More clearly defined and psychometrically transparent measures may be needed to capture psychological characteristics relevant to insider threat.

Finally, the outcome variable was highly heterogeneous and imprecise, combining pre-existing and emergent criminal and non-criminal antecedents. Furthermore, the procedures and findings of the personnel security investigations lacked transparency. Accordingly, the findings should be interpreted with caution. They should not be generalized beyond populations undergoing comparable military selection and security-investigation procedures without replication using more precise and clearly defined outcomes and psychometrically transparent measures.

### 4.8 Implications

The findings suggest that broad psychological assessments may have limited practical value for predicting risk-mitigative measures. More targeted measures of theoretically relevant constructs, such as conscientiousness, integrity, or maladaptive personality traits, may be better suited to translating knowledge of psychological vulnerabilities into operationally useful personnel security tools.

Neither the demographic associations, nor the results from the psychological assessments, support using these characteristics or measures as stand-alone screening or exclusion criteria. The association between age at assessment and investigations involving pre-existing antecedents likely reflect greater cumulative opportunity for antecedents to occur, while the sex differences may reflect broader behavioral, occupational, or reporting patterns. Moreover, using demographic characteristics to inform personnel security decisions could raise substantial legal and ethical concerns. Despite these limitations, demographics and basic psychological assessments may still have limited value in highly sensitive or risk-averse environments when used as one component of a broader evaluation, helping to flag at-risk individuals and guide implementation of more targeted, resource-intensive investigative or monitoring efforts.

Future research should prospectively evaluate targeted psychological measures at their original level of measurement and against clearly defined outcomes. Studies should also determine whether these measures provide incremental predictive value beyond existing selection and security information, and, perhaps most importantly, whether insider threat antecedents themselves meaningfully predict actual insider threat outcomes.

## 5 Conclusion

Sex and, for investigations involving pre-existing antecedents, age at assessment showed the strongest associations with adjudicated investigation status. Broad psychological assessments, however, demonstrated limited and inconsistent utility in distinguishing investigation cases from controls. These assessments may nevertheless have some operational value as one component of a broader evaluation in highly sensitive contexts, but the findings do not support their use as stand-alone screening or exclusion criteria.

## Data Availability

The original contributions presented in the study are included in the article/Supplementary material, further inquiries can be directed to the corresponding author.
