# Supplementary material for: Psychological assessments predict insider threat risk-mitigation measures, but only marginally
Source: Front Psychol. 2026 Sep 8;17:1926403. doi: 10.3389/fpsyg.2026.1926403 (PMC13593516; doi:10.3389/fpsyg.2026.1926403)
Supplement: Supplementary file 1 [file Supplementary_Tables.zip › Quarto_code.html]

Supplementary code: Psychological Assessments Predict Insider Threat Risk-Mitigation Measures, but Only Marginally


## Table of contents

- Introduction
- Dependencies
- Functions
- Demographics
  - Summary data
    - Import and transform data
  - Categories of Service
    - Import and transform data
    - Data analysis
- RQ 1: Bivariate datasets
  - 1: Age at assessment
    - Data Preparation and Visualization
    - Data analysis
  - 2: Sex
    - Data Preparation and Visualization
    - Data analysis
  - 3: Cognitive ability
    - Data Preparation and Visualization
    - Data analysis
  - 4: Psychological level of function
    - Data Preparation and Visualization
    - Data analysis
  - 5: Suitability for military command
    - Data Preparation and Visualization
    - Data analysis
- RQ 2: Combined datasets
  - Combined dataset 1: Age at assessment, sex, cognitive ability and psychological level of function
    - Data Preparation and Visualization
    - Data analysis
  - Combined dataset 2: Age at assessment, sex, cognitive ability, and suitability for military command
    - Data Preparation and Visualization
    - Data analysis

# Supplementary code: Psychological Assessments Predict Insider Threat Risk-Mitigation Measures, but Only Marginally

Published

August 30, 2026

# Introduction

This supplementary document provides a transparent and reproducible account of the personnel security register data, including the military selection assessment data, provided by the Swedish Armed Forces. The code details the data import, preprocessing, and analysis procedures referenced in the article.

The first phase included exploratory bivariate logistic regression analyses examining the potential relationships between a single predictor and two different outcome variables:

1. Adjudicated investigations involving insider threat antecedents present **prior** to the military selection assessment (Pre-existing antecedents).
2. Adjudicated investigations involving insider threat antecedents emerging **after** the military selection assessment (Emergent antecedents).

Six **bivariate datasets** containing either single predictor demographic or military selection assessment variables, was examined. The purpose of the bivariate analyses was to identify the most relevant predictors to include in a combined dataset of multiple predictors.

In the second phase of data collection and analysis, the Swedish Armed Forces provided two **combined datasets** that combined multiple predictors judged relevant to include in more complex modelling.

# Dependencies

All analyses were conducted in **R** (v4.5.0) using the following packages:

R packages used in this analysis.

| Package | Version |
| --- | --- |
| tidyverse | 2.0.0 |
| knitr | 1.50 |
| broom | 1.0.8 |
| kableExtra | 1.4.0 |
| polycor | 0.8.1 |
| glue | 1.8.0 |

# Functions

This code details two functions used to transform and present the data.

1. Logistic regression output function
2. Logistic regression model comparison function

- Logistic regression output function
- Logistic regression model comparison function

The logistic regression output function generates a table summarizing the key results from the logistic regression analyses.

Code

```
table.function <- function(model, model_name = "Model") {
  
  tidy(model, conf.int = TRUE, conf.level = 0.95) %>%
    mutate(
      Model = model_name,
      Estimate = round(estimate, 2),
      Std.Error = round(std.error, 2),
      z = round(statistic, 2),
      p_value = case_when(
        is.na(p.value) ~ NA_character_,
        p.value < 0.001 ~ "< .001",
        TRUE ~ formatC(p.value, digits = 3, format = "f")),
      Significance = case_when(
        is.na(p.value) ~ "",
        p.value < 0.001 ~ "***",
        p.value < 0.01 ~ "**",
        p.value < 0.05 ~ "*",
        TRUE ~ ""),
      odds = round(exp(estimate), 2),
      ci_low = round(exp(conf.low), 2),
      ci_high = round(exp(conf.high), 2),
      `Odds Ratio [95% CI]` = paste0(odds, " [", ci_low, ", ", ci_high, "]"),
      term = case_when(
        term == "(Intercept)" ~ "Intercept",
        term == "sexFemale" ~ "Female",
        term == "age22-25" ~ "Age 22-25",
        term == "age26-30" ~ "Age 26-30",
        term == "age31+" ~ "Age 31+",
        term == "applications2" ~ "2",
        term == "applications3" ~ "3",
        term == "applications4" ~ "4",
        term == "plf" ~ "plf rating",
        term == "ca" ~ "ca score",
        term == "smc" ~ "smc rating",
        TRUE ~ term)) %>%
    mutate(
      Variable = ifelse(term == "(Intercept)",
                        paste0("Intercept (", Model, ")"),
                        paste0(Model, ": ", term))) %>% select(
  Predictor = Variable, 
  Estimate,
  Std.Error,
  z,
  p_value,
  Significance,
  `Odds Ratio [95% CI]`)}
```

The logistic regression model comparison function performs model comparisons and presents the key results in a summary table.

Code

```
model_comparison_nagelkerke <- function(null_model, model) {
  ll_null  <- as.numeric(logLik(null_model))
  ll_model <- as.numeric(logLik(model))
  n <- tryCatch(attr(logLik(model), "nobs"), error = function(e) NULL)
  if (is.null(n)) n <- attr(logLik(null_model), "nobs")
  r2_cs <- 1 - exp((2 / n) * (ll_null - ll_model))
  denom <- 1 - exp((2 / n) * ll_null)
  r2_n <- if (isTRUE(all.equal(denom, 0))) NA_real_ else r2_cs / denom
  r2_n <- pmin(pmax(r2_n, 0), 1)  # clamp to [0,1]
  lr   <- anova(null_model, model, test = "Chisq")
  chi2 <- lr$Deviance[2]
  df   <- lr$Df[2]
  p    <- lr$`Pr(>Chi)`[2]
  p_fmt <- ifelse(is.na(p), NA,
                  ifelse(p < 0.001, "< .001", formatC(p, format = "f", digits = 3)))
  stars <- ifelse(is.na(p), "",
                  ifelse(p < 0.001, "***",
                  ifelse(p < 0.01,  "**",
                  ifelse(p < 0.05,  "*", ""))))
  tibble::tibble(
    Comparison    = "Model vs Null",
    Nagelkerke_R2 = round(r2_n, 3),
    Chi2          = chi2,
    df            = df,
    p_value       = p_fmt,
    Significance  = stars)}
```

# Demographics

This section contains summary statistics and data on categories of service.

## Summary data

Summary statistics for the investigation sample and the matched and non-matched control samples.

### Import and transform data

Code

```
summary_statistics_raw <- read_csv2("Summary_statistics.csv", col_names = FALSE, show_col_types = FALSE)
```

The imported data were preprocessed, including translating and renaming variables for improved readability. The table below presents the relevant summary statistics.

Code

```
summary_statistics_raw %>%
   rename(
      Sample = X1,
      Variable = X2,
      Value = X3) %>% 
  filter(
  !is.na(Variable),
  !is.na(Value)) %>% 
  fill(Sample) %>% 
  pivot_wider(
    names_from = Sample,    
    values_from = Value) %>%
  rename(
  "Total Investigation Sample" = "U TOTAL",
  "Total Matched Control Sample" = "Grupp 2 + 4",
  "Total Unmatched Control Sample" = "Grupp 5",
  "Pre-existing Antecedent Sample" = "Grupp 1",
  "Pre-existing Matched Control Sample" = "Grupp 2",
  "Emergent Antecedent Sample" = "Grupp 3",
  "Emergent Matched Control Sample" = "Grupp 4") %>%
  mutate(
    Variable = recode(Variable,
      "N" = "n",          
      "Max Ålder Prövn" = "max age at assessment",
      "Min Ålder Prövn" = "min age at assessment",
      "M Ålder Prövn" = "mean age at assessment",
      "SD Ålder Prövn" = "std age at assessment",
      "Max Ålder Utfall" = "max age at antecedent emergence",
      "Min Ålder Utfall" = "min age at antecedent emergence",
      "M Ålder Utfall" = "mean age at antecedent emergence",
      "SD Ålder Utfal" = "std age at antecedent emergence",
      "M ToR" = "mean years from assessment",
      "SD ToR" = "std years from assessment")) %>%
  mutate(
    across(
      -Variable,
      ~ if_else(
        grepl("^n($| )", Variable),
        formatC(.x, format = "d"),
        formatC(.x, format = "f", digits = 2)))) %>%
  kable()
```

| Variable | Total Investigation Sample | Pre-existing Antecedent Sample | Emergent Antecedent Sample | Pre-existing Matched Control Sample | Emergent Matched Control Sample | Total Unmatched Control Sample | Total Matched Control Sample |
| --- | --- | --- | --- | --- | --- | --- | --- |
| n | 650 | 289 | 361 | 289 | 361 | 650 | 650 |
| max age at assessment | 47.00 | NA | NA | NA | NA | 45.00 | 59.00 |
| min age at assessment | 18.00 | NA | NA | NA | NA | 18.00 | 18.00 |
| mean age at assessment | 22.11 | 22.87 | 21.51 | 20.99 | 21.14 | 21.00 | 21.07 |
| std age at assessment | 4.46 | 4.64 | 4.22 | 4.27 | 3.55 | 3.41 | 3.89 |
| mean age at antecedent emergence | 23.12 | 19.79 | 25.78 | NA | NA | NA | NA |
| std age at antecedent emergence | 5.53 | 4.00 | 5.12 | NA | NA | NA | NA |
| max age at antecedent emergence | NA | 38.00 | 54.00 | NA | NA | NA | NA |
| min age at antecedent emergence | NA | 15.00 | 19.00 | NA | NA | NA | NA |
| mean years from assessment | NA | -3.08 | 4.27 | NA | NA | NA | NA |
| std years from assessment | NA | 2.34 | 2.69 | NA | NA | NA | NA |

## Categories of Service

### Import and transform data

Code

```
category_of_service_raw <- read_csv2("Category_of_service.csv.", show_col_types = FALSE)
```

The imported data were preprocessed, including translating and renaming variables for improved readability. The table below presents the relevant summary statistics.

Code

```
category_summary_raw <- category_of_service_raw %>%
  rename(Group = Grupp, Category = Kategory) %>%
  mutate(
    Group = case_when(
      Group == 1 ~ "Pre-existing Antecedent Sample",
      Group == 2 ~ "Pre-existing Matched Control Sample",
      Group == 3 ~ "Emergent Antecedent Sample",
      Group == 4 ~ "Emergent Matched Control Sample",
      Group == 5 ~ "Non-matched Control Sample"),
    
    Category = case_when(
      Category == "AVTALSPERSONAL" ~ "Volunteers",
      Category == "GRUPPBEFÄL, SOLDAT, SJÖMAN" ~ "Squad leaders, soldiers and sailors",
      Category == "HEMVÄRNSSOLDAT" ~ "Home Guard",
      Category == "REKRYT" ~ "Recruits",
      Category == "YRKESOFFICER, RESERVOFFICER" ~ "Officers and specialist officers",
      Category == "SUA ARBETE" ~ "Consultants",
      Category == "CIVIL ANSTÄLLNING" ~ "Civilian employees",
      TRUE ~ NA_character_)) %>%
  filter(!is.na(Category)) %>%
  count(Category, Group) %>%

  pivot_wider(
    names_from = Group,
    values_from = n,
    values_fill = 0) %>%
  
  mutate(
    `Total Investigation Sample` =
      `Pre-existing Antecedent Sample` + `Emergent Antecedent Sample`,
    `Total Matched Control Sample` =
      `Pre-existing Matched Control Sample` + `Emergent Matched Control Sample`)

category_summary <- category_summary_raw %>%
  bind_rows(
    summarise(
      .,
      Category = "Total",
      across(where(is.numeric), \(x) sum(x, na.rm = TRUE)))) %>%
  
  mutate(
    across(
      where(is.numeric),
      ~ paste0(
        .x,
        " (",
        round(.x / .x[Category == "Total"] * 100, 1),
        "%)"))) %>%
  
  select(
    Category,
    `Pre-existing Antecedent Sample`,
    `Emergent Antecedent Sample`,
    `Total Investigation Sample`,
    `Pre-existing Matched Control Sample`,
    `Emergent Matched Control Sample`,
    `Total Matched Control Sample`,
    `Non-matched Control Sample`)

category_summary %>%
  kable(caption = "Distribution of Categories of Service by Sample")
```

Table 1: Table showing the distribution of categories of service within the different samples and subgroups.

Distribution of Categories of Service by Sample

| Category | Pre-existing Antecedent Sample | Emergent Antecedent Sample | Total Investigation Sample | Pre-existing Matched Control Sample | Emergent Matched Control Sample | Total Matched Control Sample | Non-matched Control Sample |
| --- | --- | --- | --- | --- | --- | --- | --- |
| Civilian employees | 2 (0.7%) | 8 (2.2%) | 10 (1.5%) | 2 (0.7%) | 7 (1.9%) | 9 (1.4%) | 10 (1.5%) |
| Consultants | 0 (0%) | 2 (0.6%) | 2 (0.3%) | 0 (0%) | 2 (0.6%) | 2 (0.3%) | 7 (1.1%) |
| Home Guard | 20 (6.9%) | 72 (19.9%) | 92 (14.2%) | 20 (6.9%) | 72 (19.9%) | 92 (14.2%) | 90 (13.8%) |
| Officers and specialist officers | 0 (0%) | 32 (8.9%) | 32 (4.9%) | 0 (0%) | 32 (8.9%) | 32 (4.9%) | 100 (15.4%) |
| Recruits | 236 (81.7%) | 43 (11.9%) | 279 (42.9%) | 236 (81.7%) | 44 (12.2%) | 280 (43.1%) | 55 (8.5%) |
| Squad leaders, soldiers and sailors | 24 (8.3%) | 190 (52.6%) | 214 (32.9%) | 24 (8.3%) | 190 (52.6%) | 214 (32.9%) | 355 (54.6%) |
| Volunteers | 7 (2.4%) | 14 (3.9%) | 21 (3.2%) | 7 (2.4%) | 14 (3.9%) | 21 (3.2%) | 33 (5.1%) |
| Total | 289 (100%) | 361 (100%) | 650 (100%) | 289 (100%) | 361 (100%) | 650 (100%) | 650 (100%) |

### Data analysis

A Pearson’s Chi-squared test was conducted to assess whether the distribution of categories of service differed between the Investigation sample and the non-matched control sample. The test was based on a contingency table representing the number of individuals in each category of service across the two samples.

Code

```
category_chisq_total <- category_summary_raw %>%
  select(Category, "Non-matched Control Sample", "Total Investigation Sample") %>%
  filter(Category != "Total") %>% 
  column_to_rownames("Category") %>%
  as.matrix() %>% 
  chisq.test()

print(category_chisq_total)
```

```
    Pearson's Chi-squared test

data:  .
X-squared = 225.66, df = 6, p-value < 2.2e-16
```

Standardized residuals were examined to identify which categories contributed most to any observed differences.

Code

```
category_chisq_total$stdres
```

```
                                    Non-matched Control Sample
Civilian employees                                   0.0000000
Consultants                                          1.6724660
Home Guard                                          -0.1598619
Officers and specialist officers                     6.2441338
Recruits                                           -14.2186347
Squad leaders, soldiers and sailors                  7.8827176
Volunteers                                           1.6680037
                                    Total Investigation Sample
Civilian employees                                   0.0000000
Consultants                                         -1.6724660
Home Guard                                           0.1598619
Officers and specialist officers                    -6.2441338
Recruits                                            14.2186347
Squad leaders, soldiers and sailors                 -7.8827176
Volunteers                                          -1.6680037
```

# RQ 1: Bivariate datasets

The five bivariate datasets included:

1. Age at assessment
2. Sex
3. Cognitive ability
4. Psychological level of function
5. Suitability for military command

## 1: Age at assessment

**age**: Categorical variable grouping age at the time of first completed military selection assessment into four intervals:

1. 18-21
2. 22-25
3. 26-30
4. 31 and above (31+).

The reference level is 18-21 years.

**sample**: Categorical variable indicating the sample to which the individual belongs:

1. Pre-existing antecedent sample
2. Pre-existing matched control sample
3. Emergent antecedent sample
4. Emergent matched control sample
5. Non-matched control sample

**risk**: Binary (dummy coded) outcome variable indicating whether the individual belongs to a antecedent sample (1) or a control sample (0).

### Data Preparation and Visualization

Code

```
age_raw <- read_csv2("Bivariate_dataset_age.csv.", show_col_types = FALSE)
```

- Data preprocessing
- Data visualization

The imported data were preprocessed by filtering out unused variables, translating and renaming variables for improved readability, and converting relevant variables into factors.

The first five rows of the preprocessed dataset are shown below.

Code

```
age_dataset <- age_raw %>%
  rename(
    sample = "Grupp",
    age = "Ålder",
    risk = "Utfall") %>%
  mutate(
    sample = as.numeric(sample),
    sample_group = case_when(
      sample %in% c(1, 3) ~ "Total investigation sample",
      sample %in% c(2, 4) ~ "Matched control sample",
      sample == 5 ~ "Non-matched control sample",
      TRUE ~ NA_character_),
    sample = factor(sample,
                    levels = 1:5,
                    labels = c("Pre-existing antecedent sample",
                               "Pre-existing matched control sample",
                               "Emergent antecedent sample",
                               "Emergent matched control sample",
                               "Non-matched control sample")),
    age = as.numeric(age),
    age = factor(age,
                 levels = 1:4,
                 labels = c("18-21", "22-25", "26-30", "31+")),
    
    risk = as.numeric(risk),
    risk = factor(risk,
                  levels = 0:1,
                  labels = c("Control", "Risk")))
head(age_dataset)
```

The table below displays the distribution of age at first completed military selection assessment, grouped by age brackets, within the three samples.

Code

```
age_dataset %>%
  group_by(sample_group, age) %>%
  summarise(count = n(), .groups = "drop") %>%
  group_by(sample_group) %>%
  mutate(
    total = sum(count),
    percent = round(100 * count / total, 1),
    value = paste0(count, " (", percent, "%)")) %>%
  ungroup() %>%
  select(sample_group, age, value, total) %>%
  pivot_wider(
    names_from = age,
    values_from = value) %>%
  mutate(Total = paste0(total, " (100.0%)")) %>%
  select(-total) %>%
  kable()
```

Table 2: Table showing the distribution of age at first assessment age brackets within the control samples and investigation sample.

| sample\_group | 18-21 | 22-25 | 26-30 | 31+ | Total |
| --- | --- | --- | --- | --- | --- |
| Matched control sample | 493 (75.8%) | 105 (16.2%) | 28 (4.3%) | 24 (3.7%) | 650 (100.0%) |
| Non-matched control sample | 482 (74.2%) | 118 (18.2%) | 28 (4.3%) | 22 (3.4%) | 650 (100.0%) |
| Total investigation sample | 403 (62%) | 157 (24.2%) | 56 (8.6%) | 34 (5.2%) | 650 (100.0%) |

The histogram displays the distribution of age at first completed assessment, grouped into age brackets, within the complete control samples and investigation samples.

Code

```
age_hist <- age_dataset %>% 
  group_by(sample_group, age) %>% 
  summarise(count = n(), .groups = "drop") %>% 
  complete(sample_group, age, fill = list(count = 0)) %>%
  
  ggplot(aes(x = age, y = count, fill = sample_group)) +
  geom_bar(stat = "identity", position = position_dodge(width = 0.9), alpha = 0.8) +
  
  scale_fill_manual(
    values = c("blue", "green", "red"),
    name = "Sample") +
  
  theme_minimal() +
  
  labs(
    title = "Distribution of age at assessment age brackets",
    x = "Age",
    y = "Count",
    fill = "Sample") +
  
  theme(
    axis.text.x = element_text(angle = 45, hjust = 1),
    legend.position = "right")

print(age_hist)
```

Figure 1: Histogram showing the distribution of age at assessment age brackets across the control samples and the investigation samples.

### Data analysis

Bivariate logistic regressions were used to examine the association between age at first assessment and each outcome variable (Pre-existing and Emergent antecedents). For each analysis, the antecedent group was compared with the corresponding control group. Model fit was evaluated by comparing each model to its null model.

- Pre-existing antecedents
- Emergent antecedents

- Matched control sample
- Non-matched control sample

Subset the data to include the Pre-existing antecedent sample and the Pre-existing matched control sample.

Code

```
age_pre_matched <- age_dataset %>% 
  filter(sample %in% c("Pre-existing antecedent sample", "Pre-existing matched control sample"))
```

###### Logistic regression analysis

Fits a bivariate logistic regression with age as a predictor of Pre-existing insider threat antecedents, and displays the results in a formatted table.

Code

```
age_pre_matched_glm <- glm(risk ~ age, data = age_pre_matched, family = binomial)

table.function(age_pre_matched_glm, "Age at assessment") |>
  kable(align = "lcc") |>
  kable_styling(full_width = FALSE, position = "left") |>
  add_footnote("* p < .05, ** p < .01, *** p < .001", notation = "symbol")
```

Table 3: Bivariate logistic regression results for the association between age at first assessment and pre-existing antecedents, comparing the antecedent sample to the matched control sample (n = 578).

| Predictor | Estimate | Std.Error | z | p\_value | Significance | Odds Ratio [95% CI] |
| --- | --- | --- | --- | --- | --- | --- |
| Age at assessment: Intercept | -0.43 | 0.11 | -4.06 | < .001 | \*\*\* | 0.65 [0.53, 0.8] |
| Age at assessment: Age 22-25 | 1.13 | 0.21 | 5.29 | < .001 | \*\*\* | 3.11 [2.05, 4.77] |
| Age at assessment: Age 26-30 | 1.71 | 0.37 | 4.59 | < .001 | \*\*\* | 5.53 [2.76, 12.09] |
| Age at assessment: Age 31+ | 1.07 | 0.40 | 2.65 | 0.008 | \*\* | 2.92 [1.35, 6.71] |
|  |  |  |  |  |  |  |
| --- | --- | --- | --- | --- | --- | --- |
| ^\*^ \* p < .05, \*\* p < .01, \*\*\* p < .001 |  |  |  |  |  |  |

###### Model comparison

To evaluate whether the bivariate model provided a better fit than the null model, model comparisons were conducted using Nagelkerke’s pseudo-R². Chi-square tests were then used to assess whether the increase in explained variance was statistically significant.

Code

```
age_pre_matched_glm_null <- glm(risk ~ 1, data = age_pre_matched, family = binomial)

model_comparison_nagelkerke(age_pre_matched_glm_null, age_pre_matched_glm) %>%
  kable(align = "lcc") |>
  kable_styling(full_width = FALSE, position = "left")
```

Table 4: Model comparison between the bivariate age at first assessment model and the null model in predicting Pre-existing antecedents, comparing the antecedent sample to the matched control sample.

| Comparison | Nagelkerke\_R2 | Chi2 | df | p\_value | Significance |
| --- | --- | --- | --- | --- | --- |
| Model vs Null | 0.112 | 50.48919 | 3 | < .001 | \*\*\* |

Subset the data to include the Pre-existing antecedent sample and the Pre-existing matched control sample.

Code

```
age_pre_nonmatched <- age_dataset %>% 
  filter(sample %in% c("Pre-existing antecedent sample", "Non-matched control sample"))
```

###### Logistic regression analysis

Fits a bivariate logistic regression with age at assessment as a predictor of Pre-existing insider threat antecedents, and displays the results in a formatted table.

Code

```
age_pre_nonmatched_glm <- glm(risk ~ age, data = age_pre_nonmatched, family = binomial)

table.function(age_pre_nonmatched_glm, "Age at assessment") |>
  kable(align = "lcc") |>
  kable_styling(full_width = FALSE, position = "left") |>
  add_footnote("* p < .05, ** p < .01, *** p < .001", notation = "symbol")
```

Table 5: Bivariate logistic regression results for the association between age at first assessment and pre-existing antecedents, comparing the antecedent sample to the total non-matched control sample (n = 939).

| Predictor | Estimate | Std.Error | z | p\_value | Significance | Odds Ratio [95% CI] |
| --- | --- | --- | --- | --- | --- | --- |
| Age at assessment: Intercept | -1.19 | 0.09 | -12.60 | < .001 | \*\*\* | 0.3 [0.25, 0.37] |
| Age at assessment: Age 22-25 | 0.88 | 0.17 | 5.20 | < .001 | \*\*\* | 2.42 [1.73, 3.37] |
| Age at assessment: Age 26-30 | 1.44 | 0.27 | 5.35 | < .001 | \*\*\* | 4.22 [2.5, 7.19] |
| Age at assessment: Age 31+ | 1.04 | 0.33 | 3.18 | 0.001 | \*\* | 2.83 [1.48, 5.38] |
|  |  |  |  |  |  |  |
| --- | --- | --- | --- | --- | --- | --- |
| ^\*^ \* p < .05, \*\* p < .01, \*\*\* p < .001 |  |  |  |  |  |  |

###### Model comparison

To evaluate whether the bivariate model provided a better fit than the null model, model comparisons were conducted using Nagelkerke’s pseudo-R². Chi-square tests were then used to assess whether the increase in explained variance was statistically significant.

Code

```
age_pre_nonmatched_glm_null <- glm(risk ~ 1, data = age_pre_nonmatched, family = binomial)

model_comparison_nagelkerke(age_pre_nonmatched_glm_null, age_pre_nonmatched_glm) %>%
  kable(align = "lcc") |>
  kable_styling(full_width = FALSE, position = "left")
```

Table 6: Model comparison between the bivariate age at first assessment model and the null model in predicting Pre-existing antecedents, comparing the antecedent sample to the non-matched control sample (n = 939).

| Comparison | Nagelkerke\_R2 | Chi2 | df | p\_value | Significance |
| --- | --- | --- | --- | --- | --- |
| Model vs Null | 0.075 | 51.48987 | 3 | < .001 | \*\*\* |

- Matched control sample
- Non-matched control sample

Subset the data to include the Emergent antecedent sample and the Emergent matched control sample.

Code

```
age_emergent_matched <- age_dataset %>% 
  filter(sample %in% c("Emergent antecedent sample", "Emergent matched control sample"))
```

###### Logistic regression analysis

Fits a bivariate logistic regression with age as a predictor of Emergent insider threat antecedents, and displays the results in a formatted table.

Code

```
age_emergent_matched_glm <- glm(risk ~ age, data = age_emergent_matched, family = binomial)

table.function(age_emergent_matched_glm, "Age at assessment") |>
  kable(align = "lcc") |>
  kable_styling(full_width = FALSE, position = "left") |>
  add_footnote("* p < .05, ** p < .01, *** p < .001", notation = "symbol")
```

Table 7: Bivariate logistic regression results for the association between age at first assessment and Emergent antecedents, comparing the antecedent sample to the matched control sample (n = 722).

| Predictor | Estimate | Std.Error | z | p\_value | Significance | Odds Ratio [95% CI] |
| --- | --- | --- | --- | --- | --- | --- |
| Age at assessment: Intercept | -0.04 | 0.09 | -0.48 | 0.631 |  | 0.96 [0.81, 1.14] |
| Age at assessment: Age 22-25 | 0.16 | 0.20 | 0.84 | 0.402 |  | 1.18 [0.8, 1.73] |
| Age at assessment: Age 26-30 | 0.15 | 0.34 | 0.44 | 0.661 |  | 1.16 [0.6, 2.26] |
| Age at assessment: Age 31+ | 0.11 | 0.38 | 0.29 | 0.771 |  | 1.12 [0.53, 2.39] |
|  |  |  |  |  |  |  |
| --- | --- | --- | --- | --- | --- | --- |
| ^\*^ \* p < .05, \*\* p < .01, \*\*\* p < .001 |  |  |  |  |  |  |

###### Model comparison

To evaluate whether the bivariate model provided a better fit than the null model, model comparisons were conducted using Nagelkerke’s pseudo-R². Chi-square tests were then used to assess whether the increase in explained variance was statistically significant.

Code

```
age_emergent_matched_glm_null <- glm(risk ~ 1, data = age_emergent_matched, family = binomial)

model_comparison_nagelkerke(age_emergent_matched_glm_null, age_emergent_matched_glm) %>%
  kable(align = "lcc") |>
  kable_styling(full_width = FALSE, position = "left")
```

Table 8: Model comparison between the bivariate age at first assessment model and the null model in predicting Emergent antecedents, comparing the antecedent sample to the matched control sample.

| Comparison | Nagelkerke\_R2 | Chi2 | df | p\_value | Significance |
| --- | --- | --- | --- | --- | --- |
| Model vs Null | 0.002 | 0.8563218 | 3 | 0.836 |  |

Subset the data to include the Emergent antecedent sample and the total non-matched control sample.

Code

```
age_emergent_nonmatched <- age_dataset %>% 
  filter(sample %in% c("Emergent antecedent sample", "Non-matched control sample"))
```

###### Logistic regression analysis

Fits a bivariate logistic regression with age as a predictor of Emergent insider threat antecedents, and displays the results in a formatted table.

Code

```
age_emergent_nonmatched_glm <- glm(risk ~ age, data = age_emergent_nonmatched, family = binomial)

table.function(age_emergent_nonmatched_glm, "Age at assessment") |>
  kable(align = "lcc") |>
  kable_styling(full_width = FALSE, position = "left") |>
  add_footnote("* p < .05, ** p < .01, *** p < .001", notation = "symbol")
```

Table 9: Bivariate logistic regression results for the association between age at first assessment and Emergent antecedents, comparing the antecedent sample to the total non-matched control sample (n = 1011).

| Predictor | Estimate | Std.Error | z | p\_value | Significance | Odds Ratio [95% CI] |
| --- | --- | --- | --- | --- | --- | --- |
| Age at assessment: Intercept | -0.63 | 0.08 | -8.18 | < .001 | \*\*\* | 0.53 [0.46, 0.62] |
| Age at assessment: Age 22-25 | 0.11 | 0.17 | 0.65 | 0.514 |  | 1.12 [0.8, 1.55] |
| Age at assessment: Age 26-30 | 0.30 | 0.30 | 0.98 | 0.328 |  | 1.34 [0.73, 2.42] |
| Age at assessment: Age 31+ | 0.25 | 0.34 | 0.73 | 0.467 |  | 1.28 [0.64, 2.5] |
|  |  |  |  |  |  |  |
| --- | --- | --- | --- | --- | --- | --- |
| ^\*^ \* p < .05, \*\* p < .01, \*\*\* p < .001 |  |  |  |  |  |  |

###### Model comparison

To evaluate whether the bivariate model provided a better fit than the null model, model comparisons were conducted using Nagelkerke’s pseudo-R². Chi-square tests were then used to assess whether the increase in explained variance was statistically significant.

Code

```
age_emergent_nonmatched_glm_null <- glm(risk ~ 1, data = age_emergent_nonmatched, family = binomial)

model_comparison_nagelkerke(age_emergent_nonmatched_glm_null, age_emergent_nonmatched_glm) %>%
  kable(align = "lcc") |>
  kable_styling(full_width = FALSE, position = "left")
```

Table 10: Model comparison between the bivariate age at first assessment model and the null model in predicting Emergent antecedents, comparing the antecedent sample to the non-matched control sample.

| Comparison | Nagelkerke\_R2 | Chi2 | df | p\_value | Significance |
| --- | --- | --- | --- | --- | --- |
| Model vs Null | 0.002 | 1.62129 | 3 | 0.655 |  |

## 2: Sex

**sex**: Binary categorical variable representing sex, with levels coded as 1 = male and 2 = female. The reference category in the analysis is male.

**sample**: Categorical variable indicating the sample to which the individual belongs:

1. Pre-existing antecedent sample
2. Pre-existing matched control sample
3. Emergent antecedent sample
4. Emergent matched control sample
5. Non-matched control sample

**risk**: Binary (dummy coded) outcome variable indicating whether the individual belongs to a antecedent sample (1) or a control sample (0).

### Data Preparation and Visualization

Code

```
sex_raw <- read_csv2("Bivariate_dataset_sex.csv.", show_col_types = FALSE)
```

- Data preprocessing
- Data visualization

The imported data were preprocessed by filtering out unused variables, translating and renaming variables for improved readability, and converting relevant variables into factors.

The first five rows of the preprocessed dataset are shown below.

Code

```
sex_dataset <- sex_raw %>%
  rename(
    sample = "Grupp",
    sex = "Kön",
    risk = "Utfall") %>%
  mutate(
    sample = as.numeric(sample),
    sample_group = case_when(
      sample %in% c(1, 3) ~ "Total investigation sample",
      sample %in% c(2, 4) ~ "Matched control sample",
      sample == 5 ~ "Non-matched control sample",
      TRUE ~ NA_character_),
    sample = factor(sample,
                    levels = 1:5,
                    labels = c("Pre-existing antecedent sample",
                               "Pre-existing matched control sample",
                               "Emergent antecedent sample",
                               "Emergent matched control sample",
                               "Non-matched control sample")),
    sex = as.numeric(sex),
    sex = factor(sex,
                    levels = 1:2,
                    labels = c("Male", "Female")),
    
    risk = as.numeric(risk),
    risk = factor(risk,
                  levels = 0:1,
                  labels = c("Control", "Risk")))

head(sex_dataset)
```

The table below displays the distribution of sex within the control samples and investigation sample.

Code

```
sex_dataset %>%
  group_by(sample_group, sex) %>%
  summarise(count = n(), .groups = "drop") %>%
  pivot_wider(names_from = sex, values_from = count, values_fill = 0) %>% 
  mutate(Total = Male + Female) %>% 
  kable()
```

Table 11: Table showing the distribution of sex within the control samples and investigation sample.

| sample\_group | Male | Female | Total |
| --- | --- | --- | --- |
| Matched control sample | 550 | 100 | 650 |
| Non-matched control sample | 554 | 96 | 650 |
| Total investigation sample | 615 | 35 | 650 |

The histogram displays the distribution of sex within the control samples and investigation sample.

Code

```
sex_hist <- sex_dataset %>% 
  group_by(sample_group, sex) %>% 
  summarise(count = n(), .groups = "drop") %>% 
  complete(sample_group, sex, fill = list(count = 0)) %>%
  
  ggplot(aes(x = sex, y = count, fill = sample_group)) +
  geom_bar(stat = "identity", position = position_dodge(width = 0.9), alpha = 0.8) +
  
  scale_fill_manual(
    values = c("blue", "green", "red"),
    name = "Sample") +
  
  theme_minimal() +
  
  labs(
    title = "Distribution of sex",
    x = "Age",
    y = "Count",
    fill = "Sample") +
  
  theme(
    axis.text.x = element_text(angle = 45, hjust = 1),
    legend.position = "right")

print(sex_hist)
```

Figure 2: Histogram showing the distribution of sex across the control samples and the investigation samples.

### Data analysis

Bivariate logistic regressions were used to examine the association between sex and each outcome variable (Pre-existing and Emergent antecedents). For each analysis, the antecedent group was compared with the corresponding control group. Model fit was evaluated by comparing each model to its null model.

- Pre-existing antecedents
- Emergent antecedents

- Matched control sample
- Non-matched control sample

Subset the data to include the Pre-existing antecedent sample and the Pre-existing matched control sample.

Code

```
sex_pre_matched <- sex_dataset %>% 
  filter(sample %in% c("Pre-existing antecedent sample", "Pre-existing matched control sample"))
```

###### Logistic regression analysis

Fits a bivariate logistic regression with sex as a predictor of Pre-existing insider threat antecedents, and displays the results in a formatted table.

Code

```
sex_pre_matched_glm <- glm(risk ~ sex, data = sex_pre_matched, family = binomial)

table.function(sex_pre_matched_glm, "sex") |>
  kable(align = "lcc") |>
  kable_styling(full_width = FALSE, position = "left") |>
  add_footnote("* p < .05, ** p < .01, *** p < .001", notation = "symbol")
```

Table 12: Bivariate logistic regression results for the association between sex and pre-existing antecedents, comparing the antecedent sample to the matched control sample (n = 578).

| Predictor | Estimate | Std.Error | z | p\_value | Significance | Odds Ratio [95% CI] |
| --- | --- | --- | --- | --- | --- | --- |
| sex: Intercept | 0.09 | 0.09 | 1.09 | 0.277 |  | 1.1 [0.93, 1.3] |
| sex: Female | -1.22 | 0.34 | -3.55 | < .001 | \*\*\* | 0.3 [0.14, 0.56] |
|  |  |  |  |  |  |  |
| --- | --- | --- | --- | --- | --- | --- |
| ^\*^ \* p < .05, \*\* p < .01, \*\*\* p < .001 |  |  |  |  |  |  |

###### Model comparison

To evaluate whether the bivariate model provided a better fit than the null model, model comparisons were conducted using Nagelkerke’s pseudo-R². Chi-square tests were then used to assess whether the increase in explained variance was statistically significant.

Code

```
sex_pre_matched_glm_null <- glm(risk ~ 1, data = sex_pre_matched, family = binomial)

model_comparison_nagelkerke(sex_pre_matched_glm_null, sex_pre_matched_glm) %>%
  kable(align = "lcc") |>
  kable_styling(full_width = FALSE, position = "left")
```

Table 13: Model comparison between the bivariate sex model and the null model in predicting Pre-existing antecedents, comparing the antecedent sample to the matched control sample.

| Comparison | Nagelkerke\_R2 | Chi2 | df | p\_value | Significance |
| --- | --- | --- | --- | --- | --- |
| Model vs Null | 0.033 | 14.55763 | 1 | < .001 | \*\*\* |

Subset the data to include the Pre-existing antecedent sample and the total non-matched control sample.

Code

```
sex_pre_nonmatched <- sex_dataset %>% 
  filter(sample %in% c("Pre-existing antecedent sample", "Non-matched control sample"))
```

###### Logistic regression analysis

Fits a bivariate logistic regression with sex as a predictor of Pre-existing insider threat antecedents, and displays the results in a formatted table.

Code

```
sex_pre_nonmatched_glm <- glm(risk ~ sex, data = sex_pre_nonmatched, family = binomial)

table.function(sex_pre_nonmatched_glm, "sex") |>
  kable(align = "lcc") |>
  kable_styling(full_width = FALSE, position = "left") |>
  add_footnote("* p < .05, ** p < .01, *** p < .001", notation = "symbol")
```

Table 14: Bivariate logistic regression results for the association between sex and pre-existing antecedents, comparing the antecedent sample to the total non-matched control sample (n = 939).

| Predictor | Estimate | Std.Error | z | p\_value | Significance | Odds Ratio [95% CI] |
| --- | --- | --- | --- | --- | --- | --- |
| sex: Intercept | -0.69 | 0.07 | -9.42 | < .001 | \*\*\* | 0.5 [0.43, 0.58] |
| sex: Female | -1.39 | 0.31 | -4.40 | < .001 | \*\*\* | 0.25 [0.13, 0.45] |
|  |  |  |  |  |  |  |
| --- | --- | --- | --- | --- | --- | --- |
| ^\*^ \* p < .05, \*\* p < .01, \*\*\* p < .001 |  |  |  |  |  |  |

###### Model comparison

To evaluate whether the bivariate model provided a better fit than the null model, model comparisons were conducted using Nagelkerke’s pseudo-R². Chi-square tests were then used to assess whether the increase in explained variance was statistically significant.

Code

```
sex_pre_nonmatched_glm_null <- glm(risk ~ 1, data = sex_pre_nonmatched, family = binomial)

model_comparison_nagelkerke(sex_pre_nonmatched_glm_null, sex_pre_nonmatched_glm) %>%
  kable(align = "lcc") |>
  kable_styling(full_width = FALSE, position = "left")
```

Table 15: Model comparison between the bivariate sex model and the null model in predicting Pre-existing antecedents, comparing the antecedent sample to the non-matched control sample.

| Comparison | Nagelkerke\_R2 | Chi2 | df | p\_value | Significance |
| --- | --- | --- | --- | --- | --- |
| Model vs Null | 0.039 | 26.07049 | 1 | < .001 | \*\*\* |

- Matched control sample
- Non-matched control sample

Subset the data to include the Emergent antecedent sample and the Emergent matched control sample.

Code

```
sex_emergent_matched <- sex_dataset %>% 
  filter(sample %in% c("Emergent antecedent sample", "Emergent matched control sample"))
```

###### Logistic regression analysis

Fits a bivariate logistic regression with sex as a predictor of Emergent insider threat antecedents, and displays the results in a formatted table.

Code

```
sex_emergent_matched_glm <- glm(risk ~ sex, data = sex_emergent_matched, family = binomial)

table.function(sex_emergent_matched_glm, "sex") |>
  kable(align = "lcc") |>
  kable_styling(full_width = FALSE, position = "left") |>
  add_footnote("* p < .05, ** p < .01, *** p < .001", notation = "symbol")
```

Table 16: Bivariate logistic regression results for the association between sex and Emergent antecedents, comparing the antecedent sample to the matched control sample (n = 722).

| Predictor | Estimate | Std.Error | z | p\_value | Significance | Odds Ratio [95% CI] |
| --- | --- | --- | --- | --- | --- | --- |
| sex: Intercept | 0.13 | 0.08 | 1.59 | 0.113 |  | 1.13 [0.97, 1.33] |
| sex: Female | -1.13 | 0.26 | -4.42 | < .001 | \*\*\* | 0.32 [0.19, 0.52] |
|  |  |  |  |  |  |  |
| --- | --- | --- | --- | --- | --- | --- |
| ^\*^ \* p < .05, \*\* p < .01, \*\*\* p < .001 |  |  |  |  |  |  |

###### Model comparison

To evaluate whether the bivariate model provided a better fit than the null model, model comparisons were conducted using Nagelkerke’s pseudo-R². Chi-square tests were then used to assess whether the increase in explained variance was statistically significant.

Code

```
sex_emergent_matched_glm_null <- glm(risk ~ 1, data = sex_emergent_matched, family = binomial)

model_comparison_nagelkerke(sex_emergent_matched_glm_null, sex_emergent_matched_glm) %>%
  kable(align = "lcc") |>
  kable_styling(full_width = FALSE, position = "left")
```

Table 17: Model comparison between the bivariate sex model and the null model in predicting Emergent antecedents, comparing the antecedent sample to the matched control sample.

| Comparison | Nagelkerke\_R2 | Chi2 | df | p\_value | Significance |
| --- | --- | --- | --- | --- | --- |
| Model vs Null | 0.04 | 21.85867 | 1 | < .001 | \*\*\* |

Subset the data to include the Emergent antecedent sample and the total non-matched control sample.

Code

```
sex_emergent_nonmatched <- sex_dataset %>% 
  filter(sample %in% c("Emergent antecedent sample", "Non-matched control sample"))
```

###### Logistic regression analysis

Fits a bivariate logistic regression with sex as a predictor of Emergent insider threat antecedents, and displays the results in a formatted table.

Code

```
sex_emergent_nonmatched_glm <- glm(risk ~ sex, data = sex_emergent_nonmatched, family = binomial)

table.function(sex_emergent_nonmatched_glm, "Sex") |>
  kable(align = "lcc") |>
  kable_styling(full_width = FALSE, position = "left") |>
  add_footnote("* p < .05, ** p < .01, *** p < .001", notation = "symbol")
```

Table 18: Bivariate logistic regression results for the association between sex and Emergent antecedents, comparing the antecedent sample to the total non-matched control sample (n = 1011).

| Predictor | Estimate | Std.Error | z | p\_value | Significance | Odds Ratio [95% CI] |
| --- | --- | --- | --- | --- | --- | --- |
| Sex: Intercept | -0.49 | 0.07 | -7.16 | < .001 | \*\*\* | 0.61 [0.53, 0.7] |
| Sex: Female | -0.93 | 0.24 | -3.86 | < .001 | \*\*\* | 0.39 [0.24, 0.62] |
|  |  |  |  |  |  |  |
| --- | --- | --- | --- | --- | --- | --- |
| ^\*^ \* p < .05, \*\* p < .01, \*\*\* p < .001 |  |  |  |  |  |  |

###### Model comparison

To evaluate whether the bivariate model provided a better fit than the null model, model comparisons were conducted using Nagelkerke’s pseudo-R². Chi-square tests were then used to assess whether the increase in explained variance was statistically significant.

Code

```
sex_emergent_nonmatched_glm_null <- glm(risk ~ 1, data = sex_emergent_nonmatched, family = binomial)

model_comparison_nagelkerke(sex_emergent_nonmatched_glm_null, sex_emergent_nonmatched_glm) %>%
  kable(align = "lcc") |>
  kable_styling(full_width = FALSE, position = "left")
```

Table 19: Model comparison between the bivariate sex model and the null model in predicting Emergent antecedents, comparing the antecedent sample to the non-matched control sample.

| Comparison | Nagelkerke\_R2 | Chi2 | df | p\_value | Significance |
| --- | --- | --- | --- | --- | --- |
| Model vs Null | 0.023 | 17.17789 | 1 | < .001 | \*\*\* |

## 3: Cognitive ability

**ca**: Ordinal variable representing cognitive ability scores on a three-point scale (1-3). Treated as a continuous variable in the logistic regression analyses.

**ca\_factor**: Cognitive ability represented as an factor on a three-point scale (1-3). Used when modeling cognitive ability as a categorical variable. The reference level is 2.

**sample**: Categorical variable indicating the sample to which the individual belongs:

1. Pre-existing antecedent sample
2. Pre-existing matched control sample
3. Emergent antecedent sample
4. Emergent matched control sample
5. Non-matched control sample

**risk**: Binary (dummy coded) outcome variable indicating whether the individual belongs to a antecedent sample (1) or a control sample (0).

### Data Preparation and Visualization

Code

```
ca_raw <- read_csv2("Bivariate_dataset_ca.csv.", show_col_types = FALSE)
```

- Data preprocessing
- Data visualization

The imported data were preprocessed by filtering out unused variables, translating and renaming variables for improved readability, and converting relevant variables into factors.

The first five rows of the preprocessed dataset are shown below.

Code

```
ca_dataset <- ca_raw %>%
  rename(
    sample = "Grupp",
    ca = "Gkap Agg",
    risk = "Utfall") %>%
  mutate(
    sample = as.numeric(sample),
    sample_group = case_when(
      sample %in% c(1, 3) ~ "Total investigation sample",
      sample %in% c(2, 4) ~ "Matched control sample",
      sample == 5 ~ "Non-matched control sample",
      TRUE ~ NA_character_),
    sample = factor(sample,
                    levels = 1:5,
                    labels = c("Pre-existing antecedent sample",
                               "Pre-existing matched control sample",
                               "Emergent antecedent sample",
                               "Emergent matched control sample",
                               "Non-matched control sample")),
    ca = as.numeric(ca),
    ca_factor = factor(ca, levels = 1:3),
    ca_factor = relevel(ca_factor, ref = "2"),
    risk = as.numeric(risk),
    risk = factor(risk,
                  levels = 0:1,
                  labels = c("Control", "Risk")))
head(ca_dataset)
```

The table below displays the distribution of cognitive ability scores within the control samples and investigation samples, including missing cases and descriptive statistics.

Code

```
ca_dataset %>%
  group_by(sample_group, ca) %>%
  summarise(count = n(), .groups = "drop") %>%
  pivot_wider(
    names_from = ca,
    values_from = count,
    values_fill = 0) %>%
  
  mutate(
    complete_cases = `1` + `2` + `3`,
    total = complete_cases + `NA`) %>%
  
  left_join(
    ca_dataset %>%
      group_by(sample_group) %>%
      summarise(
        mean_ca = mean(ca, na.rm = TRUE),
        sd_ca = sd(ca, na.rm = TRUE),
        .groups = "drop"),
    by = "sample_group") %>%
  
  select(
    sample_group,
    `1`, `2`, `3`,
    `NA`,
    complete_cases,
    total,
    mean_ca,
    sd_ca) %>%
  
  rename(Missing = `NA`) %>% 
  
  kable(digits = 2)
```

Table 20: Table showing the distribution of cognitive ability scores and descriptive statistics for by sample

| sample\_group | 1 | 2 | 3 | Missing | complete\_cases | total | mean\_ca | sd\_ca |
| --- | --- | --- | --- | --- | --- | --- | --- | --- |
| Matched control sample | 13 | 432 | 191 | 14 | 636 | 650 | 2.28 | 0.49 |
| Non-matched control sample | 6 | 400 | 237 | 7 | 643 | 650 | 2.36 | 0.50 |
| Total investigation sample | 6 | 454 | 186 | 4 | 646 | 650 | 2.28 | 0.47 |

The histogram displays the distribution of cognitive ability scores within the complete control samples and investigation samples.

Code

```
ca_hist <- ca_dataset %>% 
  group_by(sample_group, ca) %>% 
  summarise(count = n(), .groups = "drop") %>% 
  complete(sample_group, ca, fill = list(count = 0)) %>%
  
  ggplot(aes(x = ca, y = count, fill = sample_group)) +
  geom_bar(stat = "identity", position = position_dodge(width = 0.9), alpha = 0.8) +
  
  scale_fill_manual(
    values = c("blue", "green", "red"),
    name = "Sample") +
  
  theme_minimal() +
  
  labs(
    title = "Distribution of cognitive ability scores",
    x = "Cognitive Ability Score",
    y = "Count",
    fill = "Sample") +
  
  theme(
    axis.text.x = element_text(angle = 45, hjust = 1),
    legend.position = "right")

print(ca_hist)
```

Figure 3: Histogram showing the distribution of cognitive ability scores across the control samples and the investigation samples.

### Data analysis

Bivariate logistic regressions were used to examine the association between cognitive ability scores and each outcome variable (Pre-existing and Emergent antecedents). For each analysis, the antecedent group was compared with the corresponding control group. The first analysis treated cognitive ability as a continuous variable, while the second treated it as an ordinal factor. Model fit was evaluated by comparing each model to its null model.

- Pre-existing antecedents
- Emergent antecedents

- Matched control sample
- Non-matched control sample

Subset the data to include the Pre-existing antecedent sample and the Pre-existing matched control sample.

Code

```
ca_pre_matched <- ca_dataset %>% 
  filter(sample %in% c("Pre-existing antecedent sample", "Pre-existing matched control sample")) %>% 
  drop_na()
```

###### Logstic regression

Fits a bivariate logistic regression with cognitive ability as a predictor (continuous) of Pre-existing insider threat antecedents, and displays the results in a formatted table.

*Cognitive Ability as a continuous variable*

Code

```
ca_pre_matched_glm <- glm(risk ~ ca, data = ca_pre_matched, family = binomial)

table.function(ca_pre_matched_glm, "Cognitive ability") |>
  kable(align = "lcc") |>
  kable_styling(full_width = FALSE, position = "left") |>
  add_footnote("* p < .05, ** p < .01, *** p < .001", notation = "symbol")
```

Table 21: Bivariate logistic regression results for the association between cognitive ability scores as a continous variable and pre-existing antecedents, comparing the antecedent sample to the matched control sample (n = 567).

| Predictor | Estimate | Std.Error | z | p\_value | Significance | Odds Ratio [95% CI] |
| --- | --- | --- | --- | --- | --- | --- |
| Cognitive ability: Intercept | 0.07 | 0.42 | 0.16 | 0.869 |  | 1.07 [0.47, 2.43] |
| Cognitive ability: ca score | -0.02 | 0.18 | -0.13 | 0.900 |  | 0.98 [0.69, 1.39] |
|  |  |  |  |  |  |  |
| --- | --- | --- | --- | --- | --- | --- |
| ^\*^ \* p < .05, \*\* p < .01, \*\*\* p < .001 |  |  |  |  |  |  |

*Cognitive ability as a categorical variable*

Code

```
ca_factor_pre_matched_glm <- glm(risk ~ ca_factor, data = ca_pre_matched, family = binomial)

table.function(ca_factor_pre_matched_glm, "Cognitive ability as a factor") |>
  kable(align = "lcc") |>
  kable_styling(full_width = FALSE, position = "left") |>
  add_footnote("* p < .05, ** p < .01, *** p < .001", notation = "symbol")
```

Table 22: Bivariate logistic regression results for the association between cognitive ability scores as a categorical variable and pre-existing antecedents, comparing the antecedent sample to the matched control sample (n = 567).

| Predictor | Estimate | Std.Error | z | p\_value | Significance | Odds Ratio [95% CI] |
| --- | --- | --- | --- | --- | --- | --- |
| Cognitive ability as a factor: Intercept | 0.08 | 0.10 | 0.75 | 0.453 |  | 1.08 [0.89, 1.31] |
| Cognitive ability as a factor: ca\_factor1 | -15.64 | 650.87 | -0.02 | 0.981 |  | 0 [NA, 3.6720642145264e+22] |
| Cognitive ability as a factor: ca\_factor3 | -0.14 | 0.19 | -0.73 | 0.463 |  | 0.87 [0.61, 1.26] |
|  |  |  |  |  |  |  |
| --- | --- | --- | --- | --- | --- | --- |
| ^\*^ \* p < .05, \*\* p < .01, \*\*\* p < .001 |  |  |  |  |  |  |

###### Model comparisons

To evaluate whether the bivariate model provided a better fit than the null model, model comparisons were conducted using Nagelkerke’s pseudo-R². Chi-square tests were then used to assess whether the increase in explained variance was statistically significant.

*Cognitive ability as a continuous variable*

Code

```
ca_pre_matched_glm_null <- glm(risk ~ 1, data = ca_pre_matched, family = binomial)

model_comparison_nagelkerke(ca_pre_matched_glm_null, ca_pre_matched_glm) %>%
  kable(align = "lcc") |>
  kable_styling(full_width = FALSE, position = "left")
```

Table 23: Model comparison between the bivariate cognitive ability model (continuous) and the null model in predicting Pre-existing antecedents, comparing the antecedent sample to the matched control sample.

| Comparison | Nagelkerke\_R2 | Chi2 | df | p\_value | Significance |
| --- | --- | --- | --- | --- | --- |
| Model vs Null | 0 | 0.0156601 | 1 | 0.900 |  |

*Cognitive ability as a categorical variable*

Code

```
ca_pre_matched_glm_null <- glm(risk ~ 1, data = ca_pre_matched, family = binomial)

model_comparison_nagelkerke(ca_pre_matched_glm_null, ca_factor_pre_matched_glm) %>%
  kable(align = "lcc") |>
  kable_styling(full_width = FALSE, position = "left")
```

Table 24: Model comparison between the bivariate cognitive ability model (categorical) and the null model in predicting Pre-existing antecedents, comparing the antecedent sample to the matched control sample.

| Comparison | Nagelkerke\_R2 | Chi2 | df | p\_value | Significance |
| --- | --- | --- | --- | --- | --- |
| Model vs Null | 0.018 | 7.604819 | 2 | 0.022 | \* |

*Continuous vs categorical*

Model fit for the continuous and categorical models were compared using Akaike´s Information Criterion (AIC).

Code

```
AIC(ca_pre_matched_glm, ca_factor_pre_matched_glm)
```

Subset the data to include the Pre-existing antecedent sample and the total non-matched control sample.

Code

```
ca_pre_nonmatched <- ca_dataset %>% 
  filter(sample %in% c("Pre-existing antecedent sample", "Non-matched control sample")) %>%
  drop_na()
```

###### Logistic regression

Fits a bivariate logistic regression with cognitive ability as a predictor of Pre-existing insider threat antecedents, and displays the results in a formatted table.

*Cognitive ability as a continuous variable*

Code

```
ca_pre_nonmatched_glm <- glm(risk ~ ca, data = ca_pre_nonmatched, family = binomial)

table.function(ca_pre_nonmatched_glm, "Cognitive ability") |>
  kable(align = "lcc") |>
  kable_styling(full_width = FALSE, position = "left") |>
  add_footnote("* p < .05, ** p < .01, *** p < .001", notation = "symbol")
```

Table 25: Bivariate logistic regression results for the association between cognitive ability scores as a continous variable and pre-existing antecedents, comparing the antecedent sample to the total non-matched control sample (n = 929).

| Predictor | Estimate | Std.Error | z | p\_value | Significance | Odds Ratio [95% CI] |
| --- | --- | --- | --- | --- | --- | --- |
| Cognitive ability: Intercept | 0.03 | 0.35 | 0.08 | 0.940 |  | 1.03 [0.51, 2.06] |
| Cognitive ability: ca score | -0.36 | 0.15 | -2.40 | 0.016 | \* | 0.7 [0.52, 0.93] |
|  |  |  |  |  |  |  |
| --- | --- | --- | --- | --- | --- | --- |
| ^\*^ \* p < .05, \*\* p < .01, \*\*\* p < .001 |  |  |  |  |  |  |

*Cognitive ability as a categorical variable*

Code

```
ca_factor_pre_nonmatched_glm <- glm(risk ~ ca_factor, data = ca_pre_nonmatched, family = binomial)

table.function(ca_factor_pre_nonmatched_glm, "Cognitive ability as a factor") |>
  kable(align = "lcc") |>
  kable_styling(full_width = FALSE, position = "left") |>
  add_footnote("* p < .05, ** p < .01, *** p < .001", notation = "symbol")
```

Table 26: Bivariate logistic regression results for the association between cognitive ability scores as a categorical variable and pre-existing antecedents, comparing the antecedent sample to the total non-matched control sample (n = 929).

| Predictor | Estimate | Std.Error | z | p\_value | Significance | Odds Ratio [95% CI] |
| --- | --- | --- | --- | --- | --- | --- |
| Cognitive ability as a factor: Intercept | -0.66 | 0.09 | -7.69 | < .001 | \*\*\* | 0.52 [0.44, 0.61] |
| Cognitive ability as a factor: ca\_factor1 | -13.91 | 360.38 | -0.04 | 0.969 |  | 0 [NA, 932157251.56] |
| Cognitive ability as a factor: ca\_factor3 | -0.44 | 0.16 | -2.83 | 0.005 | \*\* | 0.64 [0.47, 0.87] |
|  |  |  |  |  |  |  |
| --- | --- | --- | --- | --- | --- | --- |
| ^\*^ \* p < .05, \*\* p < .01, \*\*\* p < .001 |  |  |  |  |  |  |

###### Model comparisons

To evaluate whether the bivariate model provided a better fit than the null model, model comparisons were conducted using Nagelkerke’s pseudo-R². Chi-square tests were then used to assess whether the increase in explained variance was statistically significant.

*Cognitive ability as a continuous variable*

Code

```
ca_pre_nonmatched_glm_null <- glm(risk ~ 1, data = ca_pre_nonmatched, family = binomial)

model_comparison_nagelkerke(ca_pre_nonmatched_glm_null, ca_pre_nonmatched_glm) %>%
  kable(align = "lcc") |>
  kable_styling(full_width = FALSE, position = "left")
```

Table 27: Model comparison between the bivariate cognitive ability model and the null model in predicting Pre-existing antecedents, comparing the antecedent sample to the non-matched control sample.

| Comparison | Nagelkerke\_R2 | Chi2 | df | p\_value | Significance |
| --- | --- | --- | --- | --- | --- |
| Model vs Null | 0.009 | 5.884886 | 1 | 0.015 | \* |

*Cognitive ability as a categorical variable*

Code

```
model_comparison_nagelkerke(ca_pre_nonmatched_glm_null, ca_factor_pre_nonmatched_glm) %>%
  kable(align = "lcc") |>
  kable_styling(full_width = FALSE, position = "left")
```

Table 28: Model comparison between the bivariate cognitive ability model (categorical) and the null model in predicting Pre-existing antecedents, comparing the antecedent sample to the nonmatched control sample.

| Comparison | Nagelkerke\_R2 | Chi2 | df | p\_value | Significance |
| --- | --- | --- | --- | --- | --- |
| Model vs Null | 0.019 | 12.65205 | 2 | 0.002 | \*\* |

*Continuous vs categorical*

Model fit for the continuous and categorical models were compared using AIC.

Code

```
AIC(ca_pre_nonmatched_glm, ca_factor_pre_nonmatched_glm)
```

- Matched control sample
- Non-matched control sample

Subset the data to include the Emergent antecedent sample and the Emergent matched control sample.

Code

```
ca_emergent_matched <- ca_dataset %>% 
  filter(sample %in% c("Emergent antecedent sample", "Emergent matched control sample")) %>% 
  drop_na()
```

###### Logistic regression analysis

Fits a bivariate logistic regression with cognitive ability as a predictor of Emergent insider threat antecedents, and displays the results in a formatted table.

*Cognitive ability as a continuous variable*

Code

```
ca_emergent_matched_glm <- glm(risk ~ ca, data = ca_emergent_matched, family = binomial)

table.function(ca_emergent_matched_glm, "Cognitive ability") |>
  kable(align = "lcc") |>
  kable_styling(full_width = FALSE, position = "left") |>
  add_footnote("* p < .05, ** p < .01, *** p < .001", notation = "symbol")
```

Table 29: Bivariate logistic regression results for the association between cognitive ability scores as a continous variable and Emergent antecedents, comparing the antecedent sample to the matched control sample n = 715).

| Predictor | Estimate | Std.Error | z | p\_value | Significance | Odds Ratio [95% CI] |
| --- | --- | --- | --- | --- | --- | --- |
| Cognitive ability: Intercept | 0.00 | 0.36 | -0.01 | 0.996 |  | 1 [0.5, 2] |
| Cognitive ability: ca score | 0.01 | 0.15 | 0.05 | 0.963 |  | 1.01 [0.75, 1.36] |
|  |  |  |  |  |  |  |
| --- | --- | --- | --- | --- | --- | --- |
| ^\*^ \* p < .05, \*\* p < .01, \*\*\* p < .001 |  |  |  |  |  |  |

*Cognitive ability as a categorical variable*

Code

```
ca_factor_emergent_matched_glm <- glm(risk ~ ca_factor, data = ca_emergent_matched, family = binomial)

table.function(ca_factor_emergent_matched_glm, "Cognitive ability as a factor") |>
  kable(align = "lcc") |>
  kable_styling(full_width = FALSE, position = "left") |>
  add_footnote("* p < .05, ** p < .01, *** p < .001", notation = "symbol")
```

Table 30: Bivariate logistic regression results for the association between cognitive ability scores as a categorical variable and Emergent antecedents, comparing the antecedent sample to the matched control sample n = 715).

| Predictor | Estimate | Std.Error | z | p\_value | Significance | Odds Ratio [95% CI] |
| --- | --- | --- | --- | --- | --- | --- |
| Cognitive ability as a factor: Intercept | 0.03 | 0.09 | 0.32 | 0.751 |  | 1.03 [0.86, 1.23] |
| Cognitive ability as a factor: ca\_factor1 | -0.32 | 0.55 | -0.58 | 0.563 |  | 0.73 [0.24, 2.13] |
| Cognitive ability as a factor: ca\_factor3 | -0.03 | 0.16 | -0.18 | 0.861 |  | 0.97 [0.7, 1.34] |
|  |  |  |  |  |  |  |
| --- | --- | --- | --- | --- | --- | --- |
| ^\*^ \* p < .05, \*\* p < .01, \*\*\* p < .001 |  |  |  |  |  |  |

###### Model comparisons

To evaluate whether the bivariate model provided a better fit than the null model, model comparisons were conducted using Nagelkerke’s pseudo-R². Chi-square tests were then used to assess whether the increase in explained variance was statistically significant.

*Cognitive ability as a continuous variable*

Code

```
ca_emergent_matched_glm_null <- glm(risk ~ 1, data = ca_emergent_matched, family = binomial)

model_comparison_nagelkerke(ca_emergent_matched_glm_null, ca_emergent_matched_glm) %>%
  kable(align = "lcc") |>
  kable_styling(full_width = FALSE, position = "left")
```

Table 31: Model comparison between the bivariate cognitive ability model and the null model in predicting Emergent antecedents, comparing the antecedent sample to the matched control sample.

| Comparison | Nagelkerke\_R2 | Chi2 | df | p\_value | Significance |
| --- | --- | --- | --- | --- | --- |
| Model vs Null | 0 | 0.0021022 | 1 | 0.963 |  |

*Cognitive ability as a categorical variable*

Code

```
model_comparison_nagelkerke(ca_emergent_matched_glm_null, ca_factor_emergent_matched_glm) %>%
  kable(align = "lcc") |>
  kable_styling(full_width = FALSE, position = "left")
```

Table 32: Model comparison between the bivariate cognitive ability model (categorical) and the null model in predicting Pre-existing antecedents, comparing the antecedent sample to the nonmatched control sample.

| Comparison | Nagelkerke\_R2 | Chi2 | df | p\_value | Significance |
| --- | --- | --- | --- | --- | --- |
| Model vs Null | 0.001 | 0.3523483 | 2 | 0.838 |  |

*Continuous vs categorical*

Model fit for the continuous and categorical models were compared using AIC.

Code

```
AIC(ca_emergent_matched_glm, ca_factor_emergent_matched_glm)
```

Subset the data to include the Emergent antecedent sample and the total non-matched control sample.

Code

```
ca_emergent_nonmatched <- ca_dataset %>% 
  filter(sample %in% c("Emergent antecedent sample", "Non-matched control sample")) %>% 
  drop_na()
```

###### Logistic regression analysis

Fits a bivariate logistic regression with cognitive ability as a predictor of Emergent insider threat antecedents, and displays the results in a formatted table.

*Cognitive ability as a continuous variable*

Code

```
ca_emergent_nonmatched_glm <- glm(risk ~ ca, data = ca_emergent_nonmatched, family = binomial)

table.function(ca_emergent_nonmatched_glm, "Cognitive ability") |>
  kable(align = "lcc") |>
  kable_styling(full_width = FALSE, position = "left") |>
  add_footnote("* p < .05, ** p < .01, *** p < .001", notation = "symbol")
```

Table 33: Bivariate logistic regression results for the association between cognitive ability scores as a continous variable and Emergent antecedents, comparing the antecedent sample to the total non-matched control sample n = 1003).

| Predictor | Estimate | Std.Error | z | p\_value | Significance | Odds Ratio [95% CI] |
| --- | --- | --- | --- | --- | --- | --- |
| Cognitive ability: Intercept | 0.17 | 0.32 | 0.55 | 0.584 |  | 1.19 [0.64, 2.23] |
| Cognitive ability: ca score | -0.33 | 0.14 | -2.41 | 0.016 | \* | 0.72 [0.55, 0.94] |
|  |  |  |  |  |  |  |
| --- | --- | --- | --- | --- | --- | --- |
| ^\*^ \* p < .05, \*\* p < .01, \*\*\* p < .001 |  |  |  |  |  |  |

*Cognitive ability as a categorical variable*

Code

```
ca_factor_emergent_nonmatched_glm <- glm(risk ~ ca_factor, data = ca_emergent_nonmatched, family = binomial)

table.function(ca_factor_emergent_nonmatched_glm, "Cognitive ability as a factor") |>
  kable(align = "lcc") |>
  kable_styling(full_width = FALSE, position = "left") |>
  add_footnote("* p < .05, ** p < .01, *** p < .001", notation = "symbol")
```

Table 34: Bivariate logistic regression results for the association between cognitive ability scores as a categorical variable and Emergent antecedents, comparing the antecedent sample to the total non-matched control sample n = 1003).

| Predictor | Estimate | Std.Error | z | p\_value | Significance | Odds Ratio [95% CI] |
| --- | --- | --- | --- | --- | --- | --- |
| Cognitive ability as a factor: Intercept | -0.48 | 0.08 | -5.96 | < .001 | \*\*\* | 0.62 [0.53, 0.72] |
| Cognitive ability as a factor: ca\_factor1 | 0.48 | 0.58 | 0.83 | 0.408 |  | 1.62 [0.5, 5.23] |
| Cognitive ability as a factor: ca\_factor3 | -0.31 | 0.14 | -2.21 | 0.027 | \* | 0.73 [0.55, 0.96] |
|  |  |  |  |  |  |  |
| --- | --- | --- | --- | --- | --- | --- |
| ^\*^ \* p < .05, \*\* p < .01, \*\*\* p < .001 |  |  |  |  |  |  |

###### Model comparisons

*Cognitive ability as a continuous variable*

To evaluate whether the bivariate model provided a better fit than the null model, model comparisons were conducted using Nagelkerke’s pseudo-R². Chi-square tests were then used to assess whether the increase in explained variance was statistically significant.

Code

```
ca_emergent_nonmatched_glm_null <- glm(risk ~ 1, data = ca_emergent_nonmatched, family = binomial)

model_comparison_nagelkerke(ca_emergent_nonmatched_glm_null, ca_emergent_nonmatched_glm) %>%
  kable(align = "lcc") |>
  kable_styling(full_width = FALSE, position = "left")
```

Table 35: Model comparison between the bivariate cognitive ability model and the null model in predicting Emergent antecedents, comparing the antecedent sample to the non-matched control sample.

| Comparison | Nagelkerke\_R2 | Chi2 | df | p\_value | Significance |
| --- | --- | --- | --- | --- | --- |
| Model vs Null | 0.008 | 5.874902 | 1 | 0.015 | \* |

*Cognitive ability as a categorical variable*

Code

```
model_comparison_nagelkerke(ca_emergent_nonmatched_glm_null, ca_factor_emergent_nonmatched_glm) %>%
  kable(align = "lcc") |>
  kable_styling(full_width = FALSE, position = "left")
```

Table 36: Model comparison between the bivariate cognitive ability model (categorical) and the null model in predicting Emergent antecedents, comparing the antecedent sample to the nonmatched control sample.

| Comparison | Nagelkerke\_R2 | Chi2 | df | p\_value | Significance |
| --- | --- | --- | --- | --- | --- |
| Model vs Null | 0.008 | 5.951318 | 2 | 0.051 |  |

*Continuous vs categorical*

Model fit for the continuous and categorical models were compared using AIC.

Code

```
AIC(ca_emergent_nonmatched_glm, ca_factor_emergent_nonmatched_glm)
```

## 4: Psychological level of function

**plf**: Ordinal variable representing psychological level of function on a three-point scale (1-3). Treated as a continuous variable in the logistic regression analyses.

**plf\_factor**: Psychological level of function represented as an ordinal factor on a three-point scale (1-3). Used when modeling Psychological level of function as a categorical variable.

**sample**: Categorical variable indicating the sample to which the individual belongs:

1. Pre-existing antecedent sample
2. Pre-existing matched control sample
3. Emergent antecedent sample
4. Emergent matched control sample
5. Non-matched control sample

**risk**: Binary (dummy coded) outcome variable indicating whether the individual belongs to a antecedent sample (1) or a control sample (0).

### Data Preparation and Visualization

Code

```
plf_raw <- read_csv2("Bivariate_dataset_plf.csv.", show_col_types = FALSE)
```

- Data preprocessing
- Data visualization

The imported data were preprocessed by filtering out unused variables, translating and renaming variables for improved readability, and converting relevant variables into factors.

The first five rows of the preprocessed dataset are shown below.

Code

```
plf_dataset <- plf_raw %>%
  rename(
    sample = "Grupp",
    plf = "Psyf Agg",
    risk = "Utfall") %>%
  select(sample, plf, risk) %>%
  mutate(
    sample = as.numeric(sample),
    sample_group = case_when(
      sample %in% c(1, 3) ~ "Total investigation sample",
      sample %in% c(2, 4) ~ "Matched control sample",
      sample == 5 ~ "Non-matched control sample",
      TRUE ~ NA_character_),
    sample = factor(sample,
                    levels = 1:5,
                    labels = c("Pre-existing antecedent sample",
                               "Pre-existing matched control sample",
                               "Emergent antecedent sample",
                               "Emergent matched control sample",
                               "Non-matched control sample")),
    plf = as.numeric(plf),
    plf_factor = factor(plf, levels = 1:3),
    plf_factor = relevel(plf_factor, ref = "2"),
    
    risk = as.numeric(risk),
    risk = factor(risk,
                  levels = 0:1,
                  labels = c("Control", "Risk")))

head(plf_dataset)
```

The table below displays the distribution of psychological level of function ratings within the control samples and investigation samples, including missing cases and descriptive statistics.

Code

```
plf_dataset %>%
  group_by(sample_group, plf) %>%
  summarise(count = n(), .groups = "drop") %>%
  pivot_wider(
    names_from = plf,
    values_from = count,
    values_fill = 0) %>%
  
  mutate(
    complete_cases = `1` + `2` + `3`,
    total = complete_cases + `NA`) %>%
  
  left_join(
   plf_dataset %>%
      group_by(sample_group) %>%
      summarise(
        mean_plf = mean(plf, na.rm = TRUE),
        sd_plf = sd(plf, na.rm = TRUE),
        .groups = "drop"),
    by = "sample_group") %>%
  
  select(
    sample_group,
    `1`, `2`, `3`,
    `NA`,
    complete_cases,
    total,
    mean_plf,
    sd_plf) %>%
  
  rename(Missing = `NA`) %>% 
  
  kable(digits = 2)
```

Table 37: Table showing the distribution of psychological level of function ratings and descriptive statistics for by sample

| sample\_group | 1 | 2 | 3 | Missing | complete\_cases | total | mean\_plf | sd\_plf |
| --- | --- | --- | --- | --- | --- | --- | --- | --- |
| Matched control sample | 16 | 425 | 185 | 24 | 626 | 650 | 2.27 | 0.50 |
| Non-matched control sample | 34 | 402 | 194 | 20 | 630 | 650 | 2.25 | 0.55 |
| Total investigation sample | 31 | 435 | 167 | 17 | 633 | 650 | 2.21 | 0.52 |

The histogram displays the distribution of psychological level of function ratings within the control samples and the investigation sample.

Code

```
plf_hist <- plf_dataset %>% 
  group_by(sample_group, plf) %>% 
  summarise(count = n(), .groups = "drop") %>% 
  complete(sample_group, plf, fill = list(count = 0)) %>%
  
  ggplot(aes(x = plf, y = count, fill = sample_group)) +
  geom_bar(stat = "identity", position = position_dodge(width = 0.9), alpha = 0.8) +
  
  scale_fill_manual(
    values = c("blue", "green", "red"),
    name = "Sample") +
  
  theme_minimal() +
  
  labs(
    title = "Distribution of psychological level of function ratings",
    x = "Psychological level of function",
    y = "Count",
    fill = "Sample") +
  
  theme(
    axis.text.x = element_text(angle = 45, hjust = 1),
    legend.position = "right")

print(plf_hist)
```

Figure 4: Histogram showing the distribution of psychological level of function ratings across the control samples and the investigation samples.

### Data analysis

Bivariate logistic regressions were used to examine the association between psychological level of function ratings and each outcome variable (Pre-existing and Emergent antecedents). For each analysis, the antecedent group was compared with the corresponding control group. The first analysis treated cognitive ability as a continuous variable, while the second treated it as an ordinal factor. Model fit was evaluated by comparing each model to its null model.

- Pre-existing antecedents
- Emergent antecedents

- Matched control sample
- Non-matched control sample

Subset the data to include the Pre-existing antecedent sample and the Pre-existing matched control sample.

Code

```
plf_pre_matched <- plf_dataset %>% 
  filter(sample %in% c("Pre-existing antecedent sample", "Pre-existing matched control sample")) %>% 
  drop_na()
```

###### Logistic regression analysis

Fits a bivariate logistic regression with psychological level of function as a predictor of Pre-existing insider threat antecedents, and displays the results in a formatted table.

*Psychological level of function as a continuous variable*

Code

```
plf_pre_matched_glm <- glm(risk ~ plf, data = plf_pre_matched, family = binomial)

table.function(plf_pre_matched_glm, "Psychological level of function") |>
  kable(align = "lcc") |>
  kable_styling(full_width = FALSE, position = "left") |>
  add_footnote("* p < .05, ** p < .01, *** p < .001", notation = "symbol")
```

Table 38: Bivariate logistic regression results for the association between psychological level of function as a continous variable and pre-existing antecedents, comparing the antecedent sample to the matched control sample n = 561).

| Predictor | Estimate | Std.Error | z | p\_value | Significance | Odds Ratio [95% CI] |
| --- | --- | --- | --- | --- | --- | --- |
| Psychological level of function: Intercept | 0.56 | 0.40 | 1.41 | 0.159 |  | 1.75 [0.81, 3.81] |
| Psychological level of function: plf rating | -0.24 | 0.17 | -1.39 | 0.163 |  | 0.78 [0.56, 1.1] |
|  |  |  |  |  |  |  |
| --- | --- | --- | --- | --- | --- | --- |
| ^\*^ \* p < .05, \*\* p < .01, \*\*\* p < .001 |  |  |  |  |  |  |

*Psychological level of function as a categorical variable*

Code

```
plf_factor_pre_matched_glm <- glm(risk ~ plf_factor, data = plf_pre_matched, family = binomial)

table.function(plf_factor_pre_matched_glm, "Psychological level of function as a factor") |>
  kable(align = "lcc") |>
  kable_styling(full_width = FALSE, position = "left") |>
  add_footnote("* p < .05, ** p < .01, *** p < .001", notation = "symbol")
```

Table 39: Bivariate logistic regression results for the association between psychological level of function as a categorical variable and pre-existing antecedents, comparing the antecedent sample to the matched control sample n = 561).

| Predictor | Estimate | Std.Error | z | p\_value | Significance | Odds Ratio [95% CI] |
| --- | --- | --- | --- | --- | --- | --- |
| Psychological level of function as a factor: Intercept | -0.02 | 0.10 | -0.20 | 0.841 |  | 0.98 [0.81, 1.19] |
| Psychological level of function as a factor: plf\_factor1 | 1.63 | 0.64 | 2.54 | 0.011 | \* | 5.1 [1.65, 22.27] |
| Psychological level of function as a factor: plf\_factor3 | -0.02 | 0.19 | -0.11 | 0.910 |  | 0.98 [0.67, 1.43] |
|  |  |  |  |  |  |  |
| --- | --- | --- | --- | --- | --- | --- |
| ^\*^ \* p < .05, \*\* p < .01, \*\*\* p < .001 |  |  |  |  |  |  |

###### Model comparisons

To evaluate whether the bivariate model provided a better fit than the null model, model comparisons were conducted using Nagelkerke’s pseudo-R². Chi-square tests were then used to assess whether the increase in explained variance was statistically significant.

*Psychological level of function as a continuous variable*

Code

```
plf_pre_matched_glm_null <- glm(risk ~ 1, data = plf_pre_matched, family = binomial)

model_comparison_nagelkerke(plf_pre_matched_glm_null, plf_pre_matched_glm) %>%
  kable(align = "lcc") |>
  kable_styling(full_width = FALSE, position = "left")
```

Table 40: Model comparison between the bivariate psychological level of function model and the null model in predicting Pre-existing antecedents, comparing the antecedent sample to the matched control sample.

| Comparison | Nagelkerke\_R2 | Chi2 | df | p\_value | Significance |
| --- | --- | --- | --- | --- | --- |
| Model vs Null | 0.005 | 1.953376 | 1 | 0.162 |  |

*Psychological level of function as a categorical variable*

Code

```
model_comparison_nagelkerke(plf_pre_matched_glm_null, plf_factor_pre_matched_glm) %>%
  kable(align = "lcc") |>
  kable_styling(full_width = FALSE, position = "left")
```

Table 41: Model comparison between the bivariate psychological level of functiony model (categorical) and the null model in predicting Pre-existing antecedents, comparing the antecedent sample to the matched control sample.

| Comparison | Nagelkerke\_R2 | Chi2 | df | p\_value | Significance |
| --- | --- | --- | --- | --- | --- |
| Model vs Null | 0.021 | 8.791474 | 2 | 0.012 | \* |

*Continuous vs categorical*

Model fit for the continuous and categorical models were compared using AIC.

Code

```
AIC(plf_pre_matched_glm, plf_factor_pre_matched_glm)
```

Subset the data to include the Pre-existing antecedent sample and the total non-matched control sample.

Code

```
plf_pre_nonmatched <- plf_dataset %>% 
  filter(sample %in% c("Pre-existing antecedent sample", "Non-matched control sample")) %>%
  drop_na()
```

###### Logistic regression abalysis

Fit a bivariate logistic regression with psychological level of function as a predictor of Pre-existing insider threat antecedents, and display the results in a formatted table.

*Psychological level of function as a continuous variable*

Code

```
plf_pre_nonmatched_glm <- glm(risk ~ plf, data = plf_pre_nonmatched, family = binomial)

table.function(plf_pre_nonmatched_glm, "Psychological level of function")|>
  kable(align = "lcc") |>
  kable_styling(full_width = FALSE, position = "left") |>
  add_footnote("* p < .05, ** p < .01, *** p < .001", notation = "symbol")
```

Table 42: Bivariate logistic regression results for the association between psychological level of function as a continous variable and pre-existing antecedents, comparing the antecedent sample to the total non-matched control sample n = 913).

| Predictor | Estimate | Std.Error | z | p\_value | Significance | Odds Ratio [95% CI] |
| --- | --- | --- | --- | --- | --- | --- |
| Psychological level of function: Intercept | -0.34 | 0.31 | -1.10 | 0.271 |  | 0.71 [0.39, 1.3] |
| Psychological level of function: plf rating | -0.21 | 0.13 | -1.55 | 0.121 |  | 0.81 [0.62, 1.06] |
|  |  |  |  |  |  |  |
| --- | --- | --- | --- | --- | --- | --- |
| ^\*^ \* p < .05, \*\* p < .01, \*\*\* p < .001 |  |  |  |  |  |  |

*Psychological level of function as a categorical variable*

Code

```
plf_factor_pre_nonmatched_glm <- glm(risk ~ plf_factor, data = plf_pre_nonmatched, family = binomial)

table.function(plf_factor_pre_nonmatched_glm, "Psychological level of function as a factor") |>
  kable(align = "lcc") |>
  kable_styling(full_width = FALSE, position = "left") |>
  add_footnote("* p < .05, ** p < .01, *** p < .001", notation = "symbol")
```

Table 43: Bivariate logistic regression results for the association between psychological level of function as a categorical variable and pre-existing antecedents, comparing the antecedent sample to the total non-matched control sample n = 913).

| Predictor | Estimate | Std.Error | z | p\_value | Significance | Odds Ratio [95% CI] |
| --- | --- | --- | --- | --- | --- | --- |
| Psychological level of function as a factor: Intercept | -0.71 | 0.09 | -8.16 | < .001 | \*\*\* | 0.49 [0.41, 0.58] |
| Psychological level of function as a factor: plf\_factor1 | -0.11 | 0.32 | -0.34 | 0.732 |  | 0.9 [0.46, 1.65] |
| Psychological level of function as a factor: plf\_factor3 | -0.31 | 0.16 | -1.89 | 0.058 |  | 0.73 [0.53, 1.01] |
|  |  |  |  |  |  |  |
| --- | --- | --- | --- | --- | --- | --- |
| ^\*^ \* p < .05, \*\* p < .01, \*\*\* p < .001 |  |  |  |  |  |  |

###### Model comparisons

To evaluate whether the bivariate model provided a better fit than the null model, model comparisons were conducted using Nagelkerke’s pseudo-R². Chi-square tests were then used to assess whether the increase in explained variance was statistically significant.

*Psychological level of function as a continuous variable*

Code

```
plf_pre_nonmatched_glm_null <- glm(risk ~ 1, data = plf_pre_nonmatched, family = binomial)

model_comparison_nagelkerke(plf_pre_nonmatched_glm_null, plf_pre_nonmatched_glm) %>%
  kable(align = "lcc") |>
  kable_styling(full_width = FALSE, position = "left")
```

Table 44: Model comparison between the bivariate psychological level of function model and the null model in predicting Pre-existing antecedents, comparing the antecedent sample to the non-matched control sample.

| Comparison | Nagelkerke\_R2 | Chi2 | df | p\_value | Significance |
| --- | --- | --- | --- | --- | --- |
| Model vs Null | 0.004 | 2.423022 | 1 | 0.120 |  |

*Psychological level of function as a categorical variable*

Code

```
model_comparison_nagelkerke(plf_pre_nonmatched_glm_null, plf_factor_pre_nonmatched_glm) %>%
  kable(align = "lcc") |>
  kable_styling(full_width = FALSE, position = "left")
```

Table 45: Model comparison between the bivariate psychological level of functiony model (categorical) and the null model in predicting Pre-existing antecedents, comparing the antecedent sample to the matched control sample.

| Comparison | Nagelkerke\_R2 | Chi2 | df | p\_value | Significance |
| --- | --- | --- | --- | --- | --- |
| Model vs Null | 0.006 | 3.668244 | 2 | 0.160 |  |

*Continuous vs categorical*

Model fit for the continuous and categorical models were compared using AIC.

Code

```
AIC(plf_pre_nonmatched_glm, plf_factor_pre_nonmatched_glm)
```

- Matched control sample
- Non-matched control sample

Subset the data to include the Emergent antecedent sample and the Emergent matched control sample.

###### Logistic regression analysis

Fit a bivariate logistic regression with psychological level of function as a predictor of Emergent insider threat antecedents, and display the results in a formatted table.

Code

```
plf_emergent_matched <- plf_dataset %>% 
  filter(sample %in% c("Emergent antecedent sample", "Emergent matched control sample")) %>%
  drop_na()
```

*Psychological level of function as a continuous variable*

Code

```
plf_emergent_matched_glm <- glm(risk ~ plf, data = plf_emergent_matched, family = binomial)

table.function(plf_emergent_matched_glm, "Psychological level of function") |>
  kable(align = "lcc") |>
  kable_styling(full_width = FALSE, position = "left") |>
  add_footnote("* p < .05, ** p < .01, *** p < .001", notation = "symbol")
```

Table 46: Bivariate logistic regression results for the association between psychological level of function as a continous variable and Emergent antecedents, comparing the antecedent sample to the matched control sample n = 698).

| Predictor | Estimate | Std.Error | z | p\_value | Significance | Odds Ratio [95% CI] |
| --- | --- | --- | --- | --- | --- | --- |
| Psychological level of function: Intercept | 0.44 | 0.34 | 1.32 | 0.187 |  | 1.56 [0.81, 3.02] |
| Psychological level of function: plf rating | -0.19 | 0.14 | -1.34 | 0.181 |  | 0.82 [0.62, 1.09] |
|  |  |  |  |  |  |  |
| --- | --- | --- | --- | --- | --- | --- |
| ^\*^ \* p < .05, \*\* p < .01, \*\*\* p < .001 |  |  |  |  |  |  |

*Psychological level of function as a categorical variable*

Code

```
plf_factor_emergent_matched_glm <- glm(risk ~ plf_factor, data = plf_emergent_matched, family = binomial)

table.function(plf_factor_emergent_matched_glm, "Psychological level of function as a factor") |>
  kable(align = "lcc") |>
  kable_styling(full_width = FALSE, position = "left") |>
  add_footnote("* p < .05, ** p < .01, *** p < .001", notation = "symbol")
```

Table 47: Bivariate logistic regression results for the association between psychological level of function as a categorical variable and Emergent antecedents, comparing the antecedent sample to the matched control sample n = 698).

| Predictor | Estimate | Std.Error | z | p\_value | Significance | Odds Ratio [95% CI] |
| --- | --- | --- | --- | --- | --- | --- |
| Psychological level of function as a factor: Intercept | 0.06 | 0.09 | 0.65 | 0.514 |  | 1.06 [0.89, 1.28] |
| Psychological level of function as a factor: plf\_factor1 | 0.15 | 0.38 | 0.38 | 0.703 |  | 1.16 [0.55, 2.5] |
| Psychological level of function as a factor: plf\_factor3 | -0.20 | 0.17 | -1.22 | 0.221 |  | 0.81 [0.59, 1.13] |
|  |  |  |  |  |  |  |
| --- | --- | --- | --- | --- | --- | --- |
| ^\*^ \* p < .05, \*\* p < .01, \*\*\* p < .001 |  |  |  |  |  |  |

###### Model comparisons

To evaluate whether the bivariate model provided a better fit than the null model, model comparisons were conducted using Nagelkerke’s pseudo-R². Chi-square tests were then used to assess whether the increase in explained variance was statistically significant.

*Psychological level of function as a continuous variable*

Code

```
plf_emergent_matched_glm_null <- glm(risk ~ 1, data = plf_emergent_matched, family = binomial)

model_comparison_nagelkerke(plf_emergent_matched_glm_null, plf_emergent_matched_glm) %>%
  kable(align = "lcc") |>
  kable_styling(full_width = FALSE, position = "left")
```

Table 48: Model comparison between the bivariate psychological level of function model and the null model in predicting Emergent antecedents, comparing the antecedent sample to the matched control sample.

| Comparison | Nagelkerke\_R2 | Chi2 | df | p\_value | Significance |
| --- | --- | --- | --- | --- | --- |
| Model vs Null | 0.003 | 1.79148 | 1 | 0.181 |  |

*Psychological level of function as a categorical variable*

Code

```
model_comparison_nagelkerke(plf_emergent_matched_glm_null, plf_factor_emergent_matched_glm) %>%
  kable(align = "lcc") |>
  kable_styling(full_width = FALSE, position = "left")
```

Table 49: Model comparison between the bivariate psychological level of function model (categorical) and the null model in predicting Emergent antecedents, comparing the antecedent sample to the matched control sample.

| Comparison | Nagelkerke\_R2 | Chi2 | df | p\_value | Significance |
| --- | --- | --- | --- | --- | --- |
| Model vs Null | 0.003 | 1.808804 | 2 | 0.405 |  |

*Continuous vs categorical*

Model fit for the continuous and categorical models were compared using AIC.

Code

```
AIC(plf_emergent_matched_glm, plf_factor_emergent_matched_glm)
```

Subset the data to include the Emergent antecedent sample and the total non-matched control sample.

Code

```
plf_emergent_nonmatched <- plf_dataset %>% 
  filter(sample %in% c("Emergent antecedent sample", "Non-matched control sample")) %>% 
  drop_na()
```

###### Logistic regression analysis

Fit a bivariate logistic regression with psychological level of function as a predictor of Emergent insider threat antecedents, and display the results in a formatted table.

*Psychological level of function as a continuous variable*

Code

```
plf_emergent_nonmatched_glm <- glm(risk ~ plf, data = plf_emergent_nonmatched, family = binomial)

table.function(plf_emergent_nonmatched_glm, "Psychological level of function") |>
  kable(align = "lcc") |>
  kable_styling(full_width = FALSE, position = "left") |>
  add_footnote("* p < .05, ** p < .01, *** p < .001", notation = "symbol")
```

Table 50: Bivariate logistic regression results for the association between psychological level of function as a continous variable and Emergent antecedents, comparing the antecedent sample to the total non-matched control sample n = 980).

| Predictor | Estimate | Std.Error | z | p\_value | Significance | Odds Ratio [95% CI] |
| --- | --- | --- | --- | --- | --- | --- |
| Psychological level of function: Intercept | -0.41 | 0.29 | -1.44 | 0.151 |  | 0.66 [0.38, 1.16] |
| Psychological level of function: plf rating | -0.08 | 0.12 | -0.63 | 0.528 |  | 0.92 [0.72, 1.18] |
|  |  |  |  |  |  |  |
| --- | --- | --- | --- | --- | --- | --- |
| ^\*^ \* p < .05, \*\* p < .01, \*\*\* p < .001 |  |  |  |  |  |  |

*Psychological level of function as a categorical variable*

Code

```
plf_factor_emergent_nonmatched_glm <- glm(risk ~ plf_factor, data = plf_emergent_nonmatched, family = binomial)

table.function(plf_factor_emergent_nonmatched_glm, "Psychological level of function as a factor") |>
  kable(align = "lcc") |>
  kable_styling(full_width = FALSE, position = "left") |>
  add_footnote("* p < .05, ** p < .01, *** p < .001", notation = "symbol")
```

Table 51: Bivariate logistic regression results for the association between psychological level of function as a categorical variable and Emergent antecedents, comparing the antecedent sample to the total non-matched control sample n = 980).

| Predictor | Estimate | Std.Error | z | p\_value | Significance | Odds Ratio [95% CI] |
| --- | --- | --- | --- | --- | --- | --- |
| Psychological level of function as a factor: Intercept | -0.53 | 0.08 | -6.45 | < .001 | \*\*\* | 0.59 [0.5, 0.69] |
| Psychological level of function as a factor: plf\_factor1 | -0.23 | 0.31 | -0.72 | 0.473 |  | 0.8 [0.42, 1.45] |
| Psychological level of function as a factor: plf\_factor3 | -0.16 | 0.15 | -1.11 | 0.269 |  | 0.85 [0.63, 1.13] |
|  |  |  |  |  |  |  |
| --- | --- | --- | --- | --- | --- | --- |
| ^\*^ \* p < .05, \*\* p < .01, \*\*\* p < .001 |  |  |  |  |  |  |

###### Model comparisons

To evaluate whether the bivariate model provided a better fit than the null model, model comparisons were conducted using Nagelkerke’s pseudo-R². Chi-square tests were then used to assess whether the increase in explained variance was statistically significant.

*Psychological level of function as a continuous variable*

Code

```
plf_emergent_nonmatched_glm_null <- glm(risk ~ 1, data = plf_emergent_nonmatched, family = binomial)

model_comparison_nagelkerke(plf_emergent_nonmatched_glm_null, plf_emergent_nonmatched_glm) %>%
  kable(align = "lcc") |>
  kable_styling(full_width = FALSE, position = "left")
```

Table 52: Model comparison between the bivariate psychological level of function model and the null model in predicting Emergent antecedents, comparing the antecedent sample to the non-matched control sample.

| Comparison | Nagelkerke\_R2 | Chi2 | df | p\_value | Significance |
| --- | --- | --- | --- | --- | --- |
| Model vs Null | 0.001 | 0.3978877 | 1 | 0.528 |  |

*Psychological level of function as a categorical variable*

Code

```
model_comparison_nagelkerke(plf_emergent_nonmatched_glm_null, plf_factor_emergent_nonmatched_glm) %>%
  kable(align = "lcc") |>
  kable_styling(full_width = FALSE, position = "left")
```

Table 53: Model comparison between the bivariate psychological level of function model (categorical) and the null model in predicting Emergent antecedents, comparing the antecedent sample to the nonmatched control sample.

| Comparison | Nagelkerke\_R2 | Chi2 | df | p\_value | Significance |
| --- | --- | --- | --- | --- | --- |
| Model vs Null | 0.002 | 1.554762 | 2 | 0.460 |  |

*Continuous vs categorical*

Model fit for the continuous and categorical models were compared using AIC.

Code

```
AIC(plf_emergent_nonmatched_glm, plf_factor_emergent_nonmatched_glm)
```

## 5: Suitability for military command

**smc**: Ordinal variable representing suitability for military command on a three-point scale (1-3). Treated as a continuous variable in the logistic regression analyses.

**smc\_factor**: Suitability for military command represented as an ordinal factor on a three-point scale (1-3). Used when modeling Suitability for military command as a categorical variable.

**sample**: Categorical variable indicating the sample to which the individual belongs:

1. Pre-existing antecedent sample
2. Pre-existing matched control sample
3. Emergent antecedent sample
4. Emergent matched control sample
5. Non-matched control sample

**risk**: Binary (dummy coded) outcome variable indicating whether the individual belongs to a antecedent sample (1) or a control sample (0).

**Kod**: Randomized code for anonymization.

**Kombination**: Unique combination of each individual’s specific variable pattern, used to detect potentially identifiable data patterns.

**Antal**: Count of individuals in the dataset sharing the exact same variable combination. Used to identify uniqueness in the data pattern.

### Data Preparation and Visualization

Code

```
smc_raw <- read_csv2("Bivariate_dataset_smc.csv.", show_col_types = FALSE)
```

- Data preprocessing
- Data visualization

The imported data were preprocessed by filtering out unused variables, translating and renaming variables for improved readability, and converting relevant variables into factors.

The first five rows of the preprocessed dataset are shown below.

Code

```
smc_dataset <- smc_raw %>%
  rename(
    sample = "Grupp",
    smc = "BF Agg",
    risk = "Utfall") %>%
  mutate(
    sample = as.numeric(sample),
    sample_group = case_when(
      sample %in% c(1, 3) ~ "Total investigation sample",
      sample %in% c(2, 4) ~ "Matched control sample",
      sample == 5 ~ "Non-matched control sample",
      TRUE ~ NA_character_),
    sample = factor(sample,
                    levels = 1:5,
                    labels = c("Pre-existing antecedent sample",
                               "Pre-existing matched control sample",
                               "Emergent antecedent sample",
                               "Emergent matched control sample",
                               "Non-matched control sample")),
    smc = as.numeric(smc),
    smc_factor = factor(smc, levels = 1:3),
    smc_factor = relevel(smc_factor, ref = "2"),
    
    risk = as.numeric(risk),
    risk = factor(risk,
                  levels = 0:1,
                  labels = c("Control", "Risk")))
head(smc_dataset)
```

The table below displays the distribution of Suitability for military command ratings within the control samples and antecedent samples, including missing cases and descriptive statistics.

Code

```
smc_dataset %>%
  group_by(sample_group, smc) %>%
  summarise(count = n(), .groups = "drop") %>%
  pivot_wider(
    names_from = smc,
    values_from = count,
    values_fill = 0) %>%
  
  mutate(
    complete_cases = `1` + `2` + `3`,
    total = complete_cases + `NA`) %>%
  
  left_join(
    smc_dataset %>%
      group_by(sample_group) %>%
      summarise(
        mean_smc = mean(smc, na.rm = TRUE),
        sd_smc = sd(smc, na.rm = TRUE),
        .groups = "drop"),
    by = "sample_group") %>%
  
  select(
    sample_group,
    `1`, `2`, `3`,
    `NA`,
    complete_cases,
    total,
    mean_smc,
    sd_smc) %>%
  
  rename(Missing = `NA`) %>% 
  
  kable(digits = 2)
```

Table 54: Table showing the distribution of suitability for military command ratings and descriptive statistics for by sample

| sample\_group | 1 | 2 | 3 | Missing | complete\_cases | total | mean\_smc | sd\_smc |
| --- | --- | --- | --- | --- | --- | --- | --- | --- |
| Matched control sample | 21 | 395 | 118 | 116 | 534 | 650 | 2.18 | 0.48 |
| Non-matched control sample | 36 | 374 | 127 | 113 | 537 | 650 | 2.17 | 0.52 |
| Total investigation sample | 44 | 385 | 91 | 130 | 520 | 650 | 2.09 | 0.50 |

The histogram displays the distribution of suitability for military command ratings within the control samples and the investigation sample.

Code

```
smc_hist <- smc_dataset %>% 
  group_by(sample_group, smc) %>% 
  summarise(count = n(), .groups = "drop") %>% 
  complete(sample_group, smc, fill = list(count = 0)) %>%
  
  ggplot(aes(x = smc, y = count, fill = sample_group)) +
  geom_bar(stat = "identity", position = position_dodge(width = 0.9), alpha = 0.8) +
  
  scale_fill_manual(
    values = c("blue", "green", "red"),
    name = "Sample") +
  
  theme_minimal() +
  
  labs(
    title = "Distribution of suitability for military command ratingss",
    x = "Suitability for military command",
    y = "Count",
    fill = "Sample") +
  
  theme(
    axis.text.x = element_text(angle = 45, hjust = 1),
    legend.position = "right")

print(smc_hist)
```

Figure 5: Histogram showing the distribution of suitability for military command ratings across the control samples and the investigation samples.

### Data analysis

Bivariate logistic regressions were used to examine the association between suitability for military command ratings and each outcome variable (Pre-existing and Emergent antecedents). For each analysis, the antecedent group was compared with the corresponding control group. The first analysis treated cognitive ability as a continuous variable, while the second treated it as an ordinal factor. Model fit was evaluated by comparing each model to its null model.

- Pre-existing antecedents
- Emergent antecedents

- Matched control sample
- Non-matched control sample

Subset the data to include the Pre-existing antecedent sample and the Pre-existing matched control sample.

Code

```
smc_pre_matched <- smc_dataset %>% 
  filter(sample %in% c("Pre-existing antecedent sample", "Pre-existing matched control sample")) %>% 
  drop_na()
```

###### Logistic regression analysis:

Fits a bivariate logistic regression with suitability for military command as a predictor of Pre-existing insider threat antecedents, and displays the results in a formatted table.

*Suitability for military command as a continuous variable*

Code

```
smc_pre_matched_glm <- glm(risk ~ smc, data = smc_pre_matched, family = binomial)

table.function(smc_pre_matched_glm, "Suitability for military command") |>
  kable(align = "lcc") |>
  kable_styling(full_width = FALSE, position = "left") |>
  add_footnote("* p < .05, ** p < .01, *** p < .001", notation = "symbol")
```

Table 55: Bivariate logistic regression results for the association between suitability for military command as a continous variable and pre-existing antecedents, comparing the antecedent sample to the matched control sample n = 458).

| Predictor | Estimate | Std.Error | z | p\_value | Significance | Odds Ratio [95% CI] |
| --- | --- | --- | --- | --- | --- | --- |
| Suitability for military command: Intercept | 0.86 | 0.42 | 2.03 | 0.043 | \* | 2.36 [1.04, 5.46] |
| Suitability for military command: smc rating | -0.41 | 0.19 | -2.12 | 0.034 | \* | 0.66 [0.45, 0.97] |
|  |  |  |  |  |  |  |
| --- | --- | --- | --- | --- | --- | --- |
| ^\*^ \* p < .05, \*\* p < .01, \*\*\* p < .001 |  |  |  |  |  |  |

*Suitability for military command as a categorical variable*

Code

```
smc_factor_pre_matched_glm <- glm(risk ~ smc_factor, data = smc_pre_matched, family = binomial)

table.function(smc_factor_pre_matched_glm, "Suitability for military command") |>
  kable(align = "lcc") |>
  kable_styling(full_width = FALSE, position = "left") |>
  add_footnote("* p < .05, ** p < .01, *** p < .001", notation = "symbol")
```

Table 56: Bivariate logistic regression results for the association between suitability for military command as a categorical variable and pre-existing antecedents, comparing the antecedent sample to the matched control sample n = 458).

| Predictor | Estimate | Std.Error | z | p\_value | Significance | Odds Ratio [95% CI] |
| --- | --- | --- | --- | --- | --- | --- |
| Suitability for military command: Intercept | -0.09 | 0.11 | -0.81 | 0.415 |  | 0.92 [0.74, 1.13] |
| Suitability for military command: smc\_factor1 | 1.43 | 0.47 | 3.04 | 0.002 | \*\* | 4.19 [1.77, 11.57] |
| Suitability for military command: smc\_factor3 | -0.04 | 0.24 | -0.19 | 0.850 |  | 0.96 [0.6, 1.52] |
|  |  |  |  |  |  |  |
| --- | --- | --- | --- | --- | --- | --- |
| ^\*^ \* p < .05, \*\* p < .01, \*\*\* p < .001 |  |  |  |  |  |  |

###### Model comparisons

To evaluate whether the bivariate model provided a better fit than the null model, model comparisons were conducted using Nagelkerke’s pseudo-R². Chi-square tests were then used to assess whether the increase in explained variance was statistically significant.

*Suitability for military command as a continuous variable*

Code

```
smc_pre_matched_glm_null <- glm(risk ~ 1, data = smc_pre_matched, family = binomial)

model_comparison_nagelkerke(smc_pre_matched_glm_null, smc_pre_matched_glm) %>%
  kable(align = "lcc") |>
  kable_styling(full_width = FALSE, position = "left")
```

Table 57: Model comparison between the bivariate suitability for military command model and the null model in predicting Pre-existing antecedents, comparing the antecedent sample to the matched control sample.

| Comparison | Nagelkerke\_R2 | Chi2 | df | p\_value | Significance |
| --- | --- | --- | --- | --- | --- |
| Model vs Null | 0.013 | 4.581925 | 1 | 0.032 | \* |

*Suitability for military command as a categorical variable*

Code

```
model_comparison_nagelkerke(smc_pre_matched_glm_null, smc_factor_pre_matched_glm) %>%
  kable(align = "lcc") |>
  kable_styling(full_width = FALSE, position = "left")
```

Table 58: Model comparison between the bivariate suitability for military command model (categorical) and the null model in predicting Pre-existing antecedents, comparing the antecedent sample to the matched control sample.

| Comparison | Nagelkerke\_R2 | Chi2 | df | p\_value | Significance |
| --- | --- | --- | --- | --- | --- |
| Model vs Null | 0.034 | 11.66252 | 2 | 0.003 | \*\* |

*Continuous vs categorical*

Model fit for the continuous and categorical models were compared using AIC.

Code

```
AIC(smc_pre_matched_glm, smc_factor_pre_matched_glm)
```

Subset the data to include the Pre-existing antecedent sample and the total non-matched control sample.

Code

```
smc_pre_nonmatched <- smc_dataset %>% 
  filter(sample %in% c("Pre-existing antecedent sample", "Non-matched control sample")) %>%
  drop_na()
```

###### Logistic regression analysis

Fit a bivariate logistic regression with suitability for military command as a predictor of Pre-existing insider threat antecedents, and display the results in a formatted table.

*Suitability for military command as a continuous variable*

Code

```
smc_pre_nonmatched_glm <- glm(risk ~ smc, data = smc_pre_nonmatched, family = binomial)

table.function(smc_pre_nonmatched_glm, "Suitability for military command") |>
  kable(align = "lcc") |>
  kable_styling(full_width = FALSE, position = "left") |>
  add_footnote("* p < .05, ** p < .01, *** p < .001", notation = "symbol")
```

Table 59: Bivariate logistic regression results for the association between suitability for military command as a continous variable and pre-existing antecedents, comparing the antecedent sample to the total non-matched control sample n = 764).

| Predictor | Estimate | Std.Error | z | p\_value | Significance | Odds Ratio [95% CI] |
| --- | --- | --- | --- | --- | --- | --- |
| Suitability for military command: Intercept | -0.20 | 0.33 | -0.60 | 0.551 |  | 0.82 [0.43, 1.57] |
| Suitability for military command: smc rating | -0.31 | 0.15 | -2.05 | 0.040 | \* | 0.73 [0.54, 0.98] |
|  |  |  |  |  |  |  |
| --- | --- | --- | --- | --- | --- | --- |
| ^\*^ \* p < .05, \*\* p < .01, \*\*\* p < .001 |  |  |  |  |  |  |

*Suitability for military command as a categorical variable*

Code

```
smc_factor_pre_nonmatched_glm <- glm(risk ~ smc_factor, data = smc_pre_nonmatched, family = binomial)

table.function(smc_factor_pre_nonmatched_glm, "Suitability for military command as a factor") |>
  kable(align = "lcc") |>
  kable_styling(full_width = FALSE, position = "left") |>
  add_footnote("* p < .05, ** p < .01, *** p < .001", notation = "symbol")
```

Table 60: Bivariate logistic regression results for the association between suitability for military command as a categorical variable and pre-existing antecedents, comparing the antecedent sample to the total non-matched control sample n = 764).

| Predictor | Estimate | Std.Error | z | p\_value | Significance | Odds Ratio [95% CI] |
| --- | --- | --- | --- | --- | --- | --- |
| Suitability for military command as a factor: Intercept | -0.84 | 0.09 | -8.90 | < .001 | \*\*\* | 0.43 [0.36, 0.52] |
| Suitability for military command as a factor: smc\_factor1 | 0.39 | 0.28 | 1.37 | 0.170 |  | 1.47 [0.84, 2.55] |
| Suitability for military command as a factor: smc\_factor3 | -0.27 | 0.20 | -1.34 | 0.180 |  | 0.76 [0.51, 1.13] |
|  |  |  |  |  |  |  |
| --- | --- | --- | --- | --- | --- | --- |
| ^\*^ \* p < .05, \*\* p < .01, \*\*\* p < .001 |  |  |  |  |  |  |

###### Model comparisons

To evaluate whether the bivariate model provided a better fit than the null model, model comparisons were conducted using Nagelkerke’s pseudo-R². Chi-square tests were then used to assess whether the increase in explained variance was statistically significant.

*Suitability for military command as a continuous variable*

Code

```
smc_pre_nonmatched_glm_null <- glm(risk ~ 1, data = smc_pre_nonmatched, family = binomial)

model_comparison_nagelkerke(smc_pre_nonmatched_glm_null, smc_pre_nonmatched_glm) %>%
  kable(align = "lcc") |>
  kable_styling(full_width = FALSE, position = "left")
```

Table 61: Model comparison between the bivariate suitability for military command model and the null model in predicting Pre-existing antecedents, comparing the antecedent sample to the non-matched control sample.

| Comparison | Nagelkerke\_R2 | Chi2 | df | p\_value | Significance |
| --- | --- | --- | --- | --- | --- |
| Model vs Null | 0.008 | 4.25204 | 1 | 0.039 | \* |

*Suitability for military command as a categorical variable*

Code

```
model_comparison_nagelkerke(smc_pre_nonmatched_glm_null, smc_factor_pre_nonmatched_glm) %>%
  kable(align = "lcc") |>
  kable_styling(full_width = FALSE, position = "left")
```

Table 62: Model comparison between the bivariate suitability for military command model (categorical) and the null model in predicting Pre-existing antecedents, comparing the antecedent sample to the nonmatched control sample.

| Comparison | Nagelkerke\_R2 | Chi2 | df | p\_value | Significance |
| --- | --- | --- | --- | --- | --- |
| Model vs Null | 0.008 | 4.35373 | 2 | 0.113 |  |

*Continuous vs categorical*

Model fit for the continuous and categorical models were compared using AIC.

Code

```
AIC(smc_pre_nonmatched_glm, smc_factor_pre_nonmatched_glm)
```

- Matched control sample
- Non-matched control sample

Subset the data to include the Emergent antecedent sample and the Emergent matched control sample.

Code

```
smc_emergent_matched <- smc_dataset %>% 
  filter(sample %in% c("Emergent antecedent sample", "Emergent matched control sample")) %>%
  drop_na()
```

###### Logistic regression analysis

Fit a bivariate logistic regression with suitability for military command as a predictor of Emergent insider threat antecedents, and display the results in a formatted table.

*Suitability for military command as a continuous variable*

Code

```
smc_emergent_matched_glm <- glm(risk ~ smc, data = smc_emergent_matched, family = binomial)

table.function(smc_emergent_matched_glm, "Suitability for military command") |>
  kable(align = "lcc") |>
  kable_styling(full_width = FALSE, position = "left") |>
  add_footnote("* p < .05, ** p < .01, *** p < .001", notation = "symbol")
```

Table 63: Bivariate logistic regression results for the association between suitability for military command as a continous variable and Emergent antecedents, comparing the antecedent sample to the matched control sample n = 596).

| Predictor | Estimate | Std.Error | z | p\_value | Significance | Odds Ratio [95% CI] |
| --- | --- | --- | --- | --- | --- | --- |
| Suitability for military command: Intercept | 0.74 | 0.37 | 1.99 | 0.047 | \* | 2.09 [1.01, 4.36] |
| Suitability for military command: smc rating | -0.36 | 0.17 | -2.13 | 0.033 | \* | 0.7 [0.5, 0.97] |
|  |  |  |  |  |  |  |
| --- | --- | --- | --- | --- | --- | --- |
| ^\*^ \* p < .05, \*\* p < .01, \*\*\* p < .001 |  |  |  |  |  |  |

*Suitability for military command as a categorical variable*

Code

```
smc_factor_emergent_matched_glm <- glm(risk ~ smc_factor, data = smc_emergent_matched, family = binomial)

table.function(smc_factor_emergent_matched_glm, "Suitability for military command as a factor") |>
  kable(align = "lcc") |>
  kable_styling(full_width = FALSE, position = "left") |>
  add_footnote("* p < .05, ** p < .01, *** p < .001", notation = "symbol")
```

Table 64: Bivariate logistic regression results for the association between suitability for military command as a categorical variable and Emergent antecedents, comparing the antecedent sample to the matched control sample n = 596).

| Predictor | Estimate | Std.Error | z | p\_value | Significance | Odds Ratio [95% CI] |
| --- | --- | --- | --- | --- | --- | --- |
| Suitability for military command as a factor: Intercept | 0.02 | 0.10 | 0.24 | 0.812 |  | 1.02 [0.85, 1.23] |
| Suitability for military command as a factor: smc\_factor1 | 0.31 | 0.35 | 0.89 | 0.372 |  | 1.37 [0.69, 2.77] |
| Suitability for military command as a factor: smc\_factor3 | -0.38 | 0.21 | -1.81 | 0.070 |  | 0.68 [0.45, 1.03] |
|  |  |  |  |  |  |  |
| --- | --- | --- | --- | --- | --- | --- |
| ^\*^ \* p < .05, \*\* p < .01, \*\*\* p < .001 |  |  |  |  |  |  |

###### Model comparisons

To evaluate whether the bivariate model provided a better fit than the null model, model comparisons were conducted using Nagelkerke’s pseudo-R². Chi-square tests were then used to assess whether the increase in explained variance was statistically significant.

*Suitability for military command as a continuous variable*

Code

```
smc_emergent_matched_glm_null <- glm(risk ~ 1, data = smc_emergent_matched, family = binomial)

model_comparison_nagelkerke(smc_emergent_matched_glm_null, smc_emergent_matched_glm) %>%
  kable(align = "lcc") |>
  kable_styling(full_width = FALSE, position = "left")
```

Table 65: Model comparison between the bivariate suitability for military command model and the null model in predicting Emergent antecedents, comparing the antecedent sample to the matched control sample.

| Comparison | Nagelkerke\_R2 | Chi2 | df | p\_value | Significance |
| --- | --- | --- | --- | --- | --- |
| Model vs Null | 0.01 | 4.595773 | 1 | 0.032 | \* |

*Suitability for military command as a categorical variable*

Code

```
model_comparison_nagelkerke(smc_emergent_matched_glm_null, smc_factor_emergent_matched_glm) %>%
  kable(align = "lcc") |>
  kable_styling(full_width = FALSE, position = "left")
```

Table 66: Model comparison between the bivariate suitability for military command model (categorical) and the null model in predicting Emergent antecedents, comparing the antecedent sample to the matched control sample.

| Comparison | Nagelkerke\_R2 | Chi2 | df | p\_value | Significance |
| --- | --- | --- | --- | --- | --- |
| Model vs Null | 0.01 | 4.61894 | 2 | 0.099 |  |

*Continuous vs categorical*

Model fit for the continuous and categorical models were compared using AIC.

Code

```
AIC(smc_emergent_matched_glm, smc_factor_emergent_matched_glm)
```

Subset the data to include the Emergent antecedent sample and the total non-matched control sample.

Code

```
smc_emergent_nonmatched <- smc_dataset %>% 
  filter(sample %in% c("Emergent antecedent sample", "Non-matched control sample")) %>% 
  drop_na()
```

###### Logistic regression analysis

Fit a bivariate logistic regression with suitability for military command as a predictor of Emergent insider threat antecedents, and display the results in a formatted table.

*Suitability for military command as a continuous variable*

Code

```
smc_emergent_nonmatched_glm <- glm(risk ~ smc, data = smc_emergent_nonmatched, family = binomial)

table.function(smc_emergent_nonmatched_glm, "Suitability for military command") |>
  kable(align = "lcc") |>
  kable_styling(full_width = FALSE, position = "left") |>
  add_footnote("* p < .05, ** p < .01, *** p < .001", notation = "symbol")
```

Table 67: Bivariate logistic regression results for the association between suitability for military command as a continous variable and Emergent antecedents, comparing the antecedent sample to the total non-matched control sample n = 830).

| Predictor | Estimate | Std.Error | z | p\_value | Significance | Odds Ratio [95% CI] |
| --- | --- | --- | --- | --- | --- | --- |
| Suitability for military command: Intercept | 0.01 | 0.31 | 0.02 | 0.985 |  | 1.01 [0.54, 1.87] |
| Suitability for military command: smc rating | -0.29 | 0.14 | -1.99 | 0.047 | \* | 0.75 [0.56, 0.99] |
|  |  |  |  |  |  |  |
| --- | --- | --- | --- | --- | --- | --- |
| ^\*^ \* p < .05, \*\* p < .01, \*\*\* p < .001 |  |  |  |  |  |  |

*Suitability for military command as a categorical variable*

Code

```
smc_factor_emergent_nonmatched_glm <- glm(risk ~ smc_factor, data = smc_emergent_nonmatched, family = binomial)

table.function(smc_factor_emergent_nonmatched_glm, "Suitability for military command as a factor") |>
  kable(align = "lcc") |>
  kable_styling(full_width = FALSE, position = "left") |>
  add_footnote("* p < .05, ** p < .01, *** p < .001", notation = "symbol")
```

Table 68: Bivariate logistic regression results for the association between suitability for military command as a categorical variable and Emergent antecedents, comparing the antecedent sample to the total non-matched control sample n = 830).

| Predictor | Estimate | Std.Error | z | p\_value | Significance | Odds Ratio [95% CI] |
| --- | --- | --- | --- | --- | --- | --- |
| Suitability for military command as a factor: Intercept | -0.52 | 0.08 | -6.11 | < .001 | \*\*\* | 0.6 [0.5, 0.7] |
| Suitability for military command as a factor: smc\_factor1 | -0.02 | 0.29 | -0.08 | 0.939 |  | 0.98 [0.55, 1.7] |
| Suitability for military command as a factor: smc\_factor3 | -0.44 | 0.19 | -2.31 | 0.021 | \* | 0.65 [0.44, 0.93] |
|  |  |  |  |  |  |  |
| --- | --- | --- | --- | --- | --- | --- |
| ^\*^ \* p < .05, \*\* p < .01, \*\*\* p < .001 |  |  |  |  |  |  |

###### Model comparisons

To evaluate whether the bivariate model provided a better fit than the null model, model comparisons were conducted using Nagelkerke’s pseudo-R². Chi-square tests were then used to assess whether the increase in explained variance was statistically significant.

*Suitability for military command as a continuous variable*

Code

```
smc_emergent_nonmatched_glm_null <- glm(risk ~ 1, data = smc_emergent_nonmatched, family = binomial)

model_comparison_nagelkerke(smc_emergent_nonmatched_glm_null, smc_emergent_nonmatched_glm) %>%
  kable(align = "lcc") |>
  kable_styling(full_width = FALSE, position = "left")
```

Table 69: Model comparison between the bivariate suitability for military command model and the null model in predicting Emergent antecedents, comparing the antecedent sample to the nonmatched control sample.

| Comparison | Nagelkerke\_R2 | Chi2 | df | p\_value | Significance |
| --- | --- | --- | --- | --- | --- |
| Model vs Null | 0.007 | 4.002181 | 1 | 0.045 | \* |

*Suitability for military command as a categorical variable*

Code

```
model_comparison_nagelkerke(smc_emergent_nonmatched_glm_null, smc_factor_emergent_nonmatched_glm) %>%
  kable(align = "lcc") |>
  kable_styling(full_width = FALSE, position = "left")
```

Table 70: Model comparison between the bivariate suitability for military command model (categorical) and the null model in predicting Emergent antecedents, comparing the antecedent sample to the nonmatched control sample.

| Comparison | Nagelkerke\_R2 | Chi2 | df | p\_value | Significance |
| --- | --- | --- | --- | --- | --- |
| Model vs Null | 0.009 | 5.603478 | 2 | 0.061 |  |

*Continuous vs categorical*

Model fit for the continuous and categorical models were compared using AIC.

Code

```
AIC(smc_emergent_nonmatched_glm, smc_factor_emergent_nonmatched_glm)
```

# RQ 2: Combined datasets

Following the bivariate analyses, two **combined datasets** was examined:

1. sex (sex), age at first assessment (age), cognitive ability (ca), and psychological level of function (plf).
2. sex (sex), age at first assessment (age), cognitive ability (with a stanine score of at least 5; ca), and suitability of military command (smc).

## Combined dataset 1: Age at assessment, sex, cognitive ability and psychological level of function

**age**: Categorical variable grouping age at the time of first completed military selection assessment into four intervals:

1. 18-21
2. 22-25
3. 26-30
4. 31 and above (31+).

The reference level is 18-21 years.

**sex**: Binary categorical variable representing sex, with levels coded as 1 = male and 2 = female. The reference category in the analysis is male.

**ca**: Ordinal variable representing cognitive ability scores on a three-point scale (1-3). Treated as a continuous variable in the logistic regression analyses.

**plf**: Ordinal variable representing psychological level of function on a three-point scale (1-3). Treated as a continuous variable in the logistic regression analyses.

**sample**: Categorical variable indicating the sample to which the individual belongs:

1. Pre-existing antecedent sample
2. Pre-existing matched control sample
3. Emergent antecedent sample
4. Emergent matched control sample
5. Non-matched control sample

**risk**: Binary (dummy coded) outcome variable indicating whether the individual belongs to a antecedent sample (1) or a control sample (0).

### Data Preparation and Visualization

Code

```
combined1_raw <- read_csv2("Combined_dataset_1.csv.", show_col_types = FALSE)
```

- Data preprocessing
- Data visualization
- Correlation tables

The imported data were preprocessed by filtering out unused variables, translating and renaming variables for improved readability, and converting relevant variables into factors.

The first five rows of the preprocessed dataset are shown below.

Code

```
combined1_dataset <- combined1_raw %>%
  rename(
    sample = "Grupp",
    sex = "Kön",
    plf = "Psyf Agg",
    ca = "Gkap Agg",
    age = "Ålderagg",
    risk = "Utfall") %>%
  mutate(
    sample = as.numeric(sample),
    sample_group = case_when(
      sample %in% c(1, 3) ~ "Total investigation sample",
      sample %in% c(2, 4) ~ "Matched control sample",
      sample == 5 ~ "Non-matched control sample",
      TRUE ~ NA_character_),
    sample = factor(sample,
                    levels = 1:5,
                    labels = c("Pre-existing antecedent sample",
                               "Pre-existing matched control sample",
                               "Emergent antecedent sample",
                               "Emergent matched control sample",
                               "Non-matched control sample")),
    sex = factor(sex,
                    levels = 1:2,
                    labels = c("Male", "Female"), ordered = FALSE),
    plf = as.numeric(plf),
    ca = as.numeric(ca),
    age = factor(age,
                 levels = 1:4,
                 labels = c("18-21", "22-25", "26-30", "31+"), ordered = FALSE),
    risk = as.numeric(risk),
    risk = factor(risk,
                    levels = 0:1,
                    labels = c("Control", "Risk"), ordered = FALSE),

    age_num = as.numeric(age),
    sex_num = as.numeric(sex) - 1,  
    risk_num = as.numeric(risk) - 1)
```

The table below displays the number of individuals per sample in the combined dataset, highlighting the reduction in sample size resulting from merging the single-predictor datasets into one combined dataset. Since all demographic predictors include 650 complete cases, the comparison focuses on the assessment variables, which vary in completeness across samples.

Code

```
ca_counts <- ca_dataset %>%
  filter(!is.na(ca)) %>%
  count(sample, name = "Cognitive ability")

plf_counts <- plf_dataset %>%
  filter(!is.na(plf)) %>%
  count(sample, name = "Psychological level of function")

combined1_counts <- combined1_dataset %>%
  count(sample, name = "Combined dataset 1")

summary_table <- full_join(ca_counts, plf_counts, by = "sample") %>%
  full_join(combined1_counts, by = "sample")

summary_table %>%
  arrange(sample) %>%
  kable() %>%
  kable_styling(full_width = FALSE, position = "left")
```

Table 71: Number of individuals per sample in each dataset, including the single-predictor datasets and the first combined dataset with multiple predictors.

| sample | Cognitive ability | Psychological level of function | Combined dataset 1 |
| --- | --- | --- | --- |
| Pre-existing antecedent sample | 286 | 283 | 270 |
| Pre-existing matched control sample | 281 | 278 | 264 |
| Emergent antecedent sample | 360 | 350 | 341 |
| Emergent matched control sample | 355 | 348 | 334 |
| Non-matched control sample | 643 | 630 | 618 |

The tables below display heterogeneous correlation matrices (maximum-likelihood estimates) for each sample in combined dataset 1. Correlations were estimated using the hetcor() function from the polycor package, which computes Pearson correlations for continuous-continuous pairs, polyserial correlations for continuous-ordinal/dichotomous pairs, and polychoric correlations for ordinal-ordinal pairs.

Age and sex were treated as ordered variables to ensure appropriate estimation of ordinal associations. Each table includes the correlation coefficient, the correlation type (in parentheses), and significance levels based on asymptotic z-tests (p < .05 *, p < .01 **, p < .001*** ). The sample size (N) reflects the number of complete cases used in each estimation.

Code

```
options(kableExtra.html.bsTable = FALSE)

combined1_dataset %>%
  filter(!is.na(sample_group)) %>%  
  group_by(sample_group) %>%
  group_walk(~ {

    .x_ord <- .x %>%
      mutate(
        age = ordered(age, levels = c("18-21","22-25","26-30","31+")),
        sex = ordered(sex, levels = c("Male","Female"))
      )
    
    res <- .x_ord %>%
      select(age, sex, ca, plf) %>%
      drop_na() %>%
      as.data.frame() %>%
      hetcor(ML = TRUE)
    
    rmat <- res$correlations
    tmat <- res$type
    se   <- res$std.errors

    zmat <- rmat / se
    pmat <- 2 * pnorm(-abs(zmat))

    stars <- function(p)
      ifelse(is.na(p), "",
             ifelse(p < .001, "***",
             ifelse(p < .01,  "**",
             ifelse(p < .05, "*", ""))))

    rround <- round(rmat, 2)
    lab <- matrix(
      sprintf("%.2f (%s)%s",
              rround,
              ifelse(tmat == "", "—", tmat),
              stars(pmat)),
      nrow = nrow(rmat), dimnames = dimnames(rmat)
    )
    diag(lab) <- ""
    lab[upper.tri(lab)] <- ""

    N <- .x_ord %>% drop_na(plf, ca, age, sex) %>% nrow()
    
    kable(
      lab, align = "l",
      caption = paste0(
        "Combined dataset 1: Heterogeneous correlations (ML) — ",
        unique(.y$sample_group),
        " (N = ", N, ")")) %>%
      footnote(
        general = paste(
          "Note. Correlations are maximum-likelihood estimates:",
          "Pearson between continuous variables;",
          "polyserial between continuous and ordinal/dichotomous variables;",
          "polychoric between ordinal variables (tetrachoric when both dichotomous).",
          "Age coded as ordered categories (18-21 < 22-25 < 26-30 < 31+);",
          "sex treated as an ordered dichotomous variable solely for estimation.",
          "p < .05 *, p < .01 **, p < .001 ***."),
        general_title = "") %>%
      print()})
```

Combined dataset 1: Heterogeneous correlations (ML) — Matched control sample (N = 598)

|  | age | sex | ca | plf |
| --- | --- | --- | --- | --- |
| age |  |  |  |  |
| sex | 0.17 (Polychoric)\* |  |  |  |
| ca | 0.12 (Polyserial)\* | -0.20 (Polyserial)\*\* |  |  |
| plf | 0.11 (Polyserial)\* | 0.12 (Polyserial) | 0.12 (Pearson)\*\* |  |
|  |  |  |  |  |
| --- | --- | --- | --- | --- |
| Note. Correlations are maximum-likelihood estimates: Pearson between continuous variables; polyserial between continuous and ordinal/dichotomous variables; polychoric between ordinal variables (tetrachoric when both dichotomous). Age coded as ordered categories (18-21 < 22-25 < 26-30 < 31+); sex treated as an ordered dichotomous variable solely for estimation. p < .05 *, p < .01 **, p < .001*** . |  |  |  |  |

Combined dataset 1: Heterogeneous correlations (ML) — Non-matched control sample (N = 618)

|  | age | sex | ca | plf |
| --- | --- | --- | --- | --- |
| age |  |  |  |  |
| sex | -0.06 (Polychoric) |  |  |  |
| ca | 0.06 (Polyserial) | -0.08 (Polyserial) |  |  |
| plf | -0.05 (Polyserial) | -0.05 (Polyserial) | 0.15 (Pearson)\*\*\* |  |
|  |  |  |  |  |
| --- | --- | --- | --- | --- |
| Note. Correlations are maximum-likelihood estimates: Pearson between continuous variables; polyserial between continuous and ordinal/dichotomous variables; polychoric between ordinal variables (tetrachoric when both dichotomous). Age coded as ordered categories (18-21 < 22-25 < 26-30 < 31+); sex treated as an ordered dichotomous variable solely for estimation. p < .05 *, p < .01 **, p < .001*** . |  |  |  |  |

Combined dataset 1: Heterogeneous correlations (ML) — Total investigation sample (N = 611)

|  | age | sex | ca | plf |
| --- | --- | --- | --- | --- |
| age |  |  |  |  |
| sex | 0.02 (Polychoric) |  |  |  |
| ca | 0.05 (Polyserial) | -0.18 (Polyserial) |  |  |
| plf | -0.05 (Polyserial) | 0.09 (Polyserial) | 0.10 (Pearson)\* |  |
|  |  |  |  |  |
| --- | --- | --- | --- | --- |
| Note. Correlations are maximum-likelihood estimates: Pearson between continuous variables; polyserial between continuous and ordinal/dichotomous variables; polychoric between ordinal variables (tetrachoric when both dichotomous). Age coded as ordered categories (18-21 < 22-25 < 26-30 < 31+); sex treated as an ordered dichotomous variable solely for estimation. p < .05 *, p < .01 **, p < .001*** . |  |  |  |  |

### Data analysis

- Pre-existing antecedents - Matched control sample
- Pre-existing antecedents - Non-matched control sample
- Emergent antecedents - Matched control sample
- Emergent antecedents - Non-matched control sample

- Multivariate regression analysis
- Bivariate analyses
- Model comparison

Fits a multivariate logistic regression and presents the results in a formatted table.

Code

```
combined1_pre_matched_glm <- glm(risk ~ sex + age + plf + ca, data = combined1_pre_matched, family = binomial)

table.function(combined1_pre_matched_glm, "Combined1") |>
  kable(align = "lcc") |>
  kable_styling(full_width = FALSE, position = "left") |>
  add_footnote("* p < .05, ** p < .01, *** p < .001", notation = "symbol")
```

Table 72: Multivariate logistic regression results examining the association between demographic and assessment predictors and pre-existing antecedents, comparing the antecedent sample to the matched control sample (n = 534).

| Predictor | Estimate | Std.Error | z | p\_value | Significance | Odds Ratio [95% CI] |
| --- | --- | --- | --- | --- | --- | --- |
| Combined1: Intercept | 1.16 | 0.62 | 1.86 | 0.063 |  | 3.19 [0.95, 10.94] |
| Combined1: Female | -1.61 | 0.45 | -3.56 | < .001 | \*\*\* | 0.2 [0.08, 0.46] |
| Combined1: Age 22-25 | 1.06 | 0.22 | 4.79 | < .001 | \*\*\* | 2.9 [1.89, 4.51] |
| Combined1: Age 26-30 | 1.73 | 0.42 | 4.15 | < .001 | \*\*\* | 5.64 [2.61, 13.64] |
| Combined1: Age 31+ | 1.35 | 0.59 | 2.30 | 0.022 | \* | 3.85 [1.28, 13.39] |
| Combined1: plf rating | -0.18 | 0.20 | -0.90 | 0.368 |  | 0.84 [0.57, 1.23] |
| Combined1: ca score | -0.46 | 0.21 | -2.20 | 0.028 | \* | 0.63 [0.42, 0.95] |
|  |  |  |  |  |  |  |
| --- | --- | --- | --- | --- | --- | --- |
| ^\*^ \* p < .05, \*\* p < .01, \*\*\* p < .001 |  |  |  |  |  |  |

###### Age at assessment

While the 31+ age bracket was significantly associated with Pre-existing antecedents in the earlier bivariate logistic regression analyses, it was not significant in the combined dataset. To explore whether this change was due to interactions with other predictors or the reduced sample size in the combined dataset, a separate bivariate logistic regression analysis was conducted using only the age at assessment predictor within the combined dataset.

Code

```
combined1_pre_matched_age_glm <- glm(risk ~ age, data = combined1_pre_matched, family = binomial)

table.function(combined1_pre_matched_age_glm, "Combined model ") |>
  kable(align = "lcc") |>
  kable_styling(full_width = FALSE, position = "left") |>
  add_footnote("* p < .05, ** p < .01, *** p < .001", notation = "symbol")
```

Table 73: Bivariate logistic regression results examining the association between the age at assessment and pre-existing antecedents, comparing the antecedent sample to the matched control sample (n = 534).

| Predictor | Estimate | Std.Error | z | p\_value | Significance | Odds Ratio [95% CI] |
| --- | --- | --- | --- | --- | --- | --- |
| Combined model : Intercept | -0.37 | 0.11 | -3.44 | < .001 | \*\*\* | 0.69 [0.56, 0.85] |
| Combined model : Age 22-25 | 1.07 | 0.22 | 4.89 | < .001 | \*\*\* | 2.9 [1.9, 4.48] |
| Combined model : Age 26-30 | 1.73 | 0.41 | 4.20 | < .001 | \*\*\* | 5.62 [2.63, 13.46] |
| Combined model : Age 31+ | 1.16 | 0.55 | 2.11 | 0.035 | \* | 3.19 [1.14, 10.31] |
|  |  |  |  |  |  |  |
| --- | --- | --- | --- | --- | --- | --- |
| ^\*^ \* p < .05, \*\* p < .01, \*\*\* p < .001 |  |  |  |  |  |  |

To evaluate whether the multivariate model provided a better fit than the null model, model comparisons were conducted using Nagelkerke’s pseudo-R². Chi-square tests were then used to assess whether the increase in explained variance was statistically significant.

Code

```
combined1_pre_matched_null <- glm(risk ~ 1, data = combined1_pre_matched, family = binomial)

model_comparison_nagelkerke(combined1_pre_matched_null, combined1_pre_matched_glm) %>%
  kable(align = "lcc") |>
  kable_styling(full_width = FALSE, position = "left")
```

Table 74: Model comparison between the first combined multivariate model and the null model in predicting Pre-existing antecedents, comparing the antecedent sample to the matched control sample.

| Comparison | Nagelkerke\_R2 | Chi2 | df | p\_value | Significance |
| --- | --- | --- | --- | --- | --- |
| Model vs Null | 0.149 | 63.43187 | 6 | < .001 | \*\*\* |

- Multivariate regression analysis
- Bivariate analysis
- Model comparison

Fits a multivariate logistic regression and presents the results in a formatted table.

Code

```
combined1_pre_nonmatched_glm <- glm(risk ~ sex + age + plf + ca, data = combined1_pre_nonmatched, family = binomial)

table.function(combined1_pre_nonmatched_glm, "Combined1") |>
  kable(align = "lcc") |>
  kable_styling(full_width = FALSE, position = "left") |>
  add_footnote("* p < .05, ** p < .01, *** p < .001", notation = "symbol")
```

Table 75: Multivariate logistic regression results examining the association between demographic and assessment predictors and pre-existing antecedents, comparing the antecedent sample to the total non-matched control sample (n = 888).

| Predictor | Estimate | Std.Error | z | p\_value | Significance | Odds Ratio [95% CI] |
| --- | --- | --- | --- | --- | --- | --- |
| Combined1: Intercept | 0.63 | 0.49 | 1.28 | 0.199 |  | 1.89 [0.72, 4.99] |
| Combined1: Female | -1.82 | 0.41 | -4.49 | < .001 | \*\*\* | 0.16 [0.07, 0.33] |
| Combined1: Age 22-25 | 0.91 | 0.18 | 5.11 | < .001 | \*\*\* | 2.49 [1.76, 3.54] |
| Combined1: Age 26-30 | 1.46 | 0.30 | 4.93 | < .001 | \*\*\* | 4.3 [2.42, 7.75] |
| Combined1: Age 31+ | 0.33 | 0.39 | 0.84 | 0.399 |  | 1.39 [0.63, 2.93] |
| Combined1: plf rating | -0.14 | 0.15 | -0.97 | 0.333 |  | 0.87 [0.65, 1.16] |
| Combined1: ca score | -0.59 | 0.17 | -3.44 | < .001 | \*\*\* | 0.56 [0.4, 0.77] |
|  |  |  |  |  |  |  |
| --- | --- | --- | --- | --- | --- | --- |
| ^\*^ \* p < .05, \*\* p < .01, \*\*\* p < .001 |  |  |  |  |  |  |

While the 31+ age bracket was significantly associated with Pre-existing antecedents in the earlier bivariate logistic regression analyses, it was not significant in the combined dataset. To explore whether this change was due to interactions with other predictors or the reduced sample size in the combined dataset, a separate bivariate logistic regression analysis was conducted using only the age at assessment predictor within the combined dataset.

###### Age at assessment

Code

```
combined1_pre_nonmatched_age_glm <- glm(risk ~ age, data = combined1_pre_nonmatched, family = binomial)

table.function(combined1_pre_nonmatched_age_glm, "Combined model 1") |>
  kable(align = "lcc") |>
  kable_styling(full_width = FALSE, position = "left") |>
  add_footnote("* p < .05, ** p < .01, *** p < .001", notation = "symbol")
```

Table 76: Bivariate logistic regression results examining the association between the age at assessment and pre-existing antecedents, comparing the antecedent sample to the non-matched control sample (n = 888).

| Predictor | Estimate | Std.Error | z | p\_value | Significance | Odds Ratio [95% CI] |
| --- | --- | --- | --- | --- | --- | --- |
| Combined model 1: Intercept | -1.17 | 0.10 | -12.24 | < .001 | \*\*\* | 0.31 [0.26, 0.37] |
| Combined model 1: Age 22-25 | 0.91 | 0.17 | 5.22 | < .001 | \*\*\* | 2.48 [1.76, 3.48] |
| Combined model 1: Age 26-30 | 1.38 | 0.29 | 4.85 | < .001 | \*\*\* | 3.99 [2.28, 7.02] |
| Combined model 1: Age 31+ | 0.52 | 0.38 | 1.36 | 0.175 |  | 1.68 [0.77, 3.51] |
|  |  |  |  |  |  |  |
| --- | --- | --- | --- | --- | --- | --- |
| ^\*^ \* p < .05, \*\* p < .01, \*\*\* p < .001 |  |  |  |  |  |  |

To evaluate whether the multivariate model provided a better fit than the null model, model comparisons were conducted using Nagelkerke’s pseudo-R². Chi-square tests were then used to assess whether the increase in explained variance was statistically significant.

Code

```
combined1_pre_nonmatched_null <- glm(risk ~ 1, data = combined1_pre_nonmatched, family = binomial)

model_comparison_nagelkerke(combined1_pre_nonmatched_null, combined1_pre_nonmatched_glm) %>%
  kable(align = "lcc") |>
  kable_styling(full_width = FALSE, position = "left")
```

Table 77: Model comparison between the first combined multivariate model and the null model in predicting Pre-existing antecedents, comparing the antecedent sample to the non-matched control sample.

| Comparison | Nagelkerke\_R2 | Chi2 | df | p\_value | Significance |
| --- | --- | --- | --- | --- | --- |
| Model vs Null | 0.132 | 87.13202 | 6 | < .001 | \*\*\* |

- Multivariate regression analysis
- Bivariate analysis
- Model comparison

Fits a multivariate logistic regression and presents the results in a formatted table.

Code

```
combined1_emergent_matched_glm <- glm(risk ~ sex + age + plf + ca, data = combined1_emergent_matched, family = binomial)

table.function(combined1_emergent_matched_glm, "Combined Modell 1") |>
  kable(align = "lcc") |>
  kable_styling(full_width = FALSE, position = "left") |>
  add_footnote("* p < .05, ** p < .01, *** p < .001", notation = "symbol")
```

Table 78: Multivariate logistic regression results examining the association between demographic and assessment predictors and Emergent antecedents, comparing the antecedent sample to the matched control sample (n = 675).

| Predictor | Estimate | Std.Error | z | p\_value | Significance | Odds Ratio [95% CI] |
| --- | --- | --- | --- | --- | --- | --- |
| Combined Modell 1: Intercept | 0.65 | 0.51 | 1.28 | 0.200 |  | 1.92 [0.71, 5.2] |
| Combined Modell 1: Female | -1.53 | 0.31 | -5.00 | < .001 | \*\*\* | 0.22 [0.11, 0.38] |
| Combined Modell 1: Age 22-25 | 0.24 | 0.21 | 1.15 | 0.249 |  | 1.27 [0.85, 1.92] |
| Combined Modell 1: Age 26-30 | 0.32 | 0.39 | 0.82 | 0.410 |  | 1.38 [0.64, 3] |
| Combined Modell 1: Age 31+ | 0.51 | 0.47 | 1.10 | 0.274 |  | 1.67 [0.67, 4.34] |
| Combined Modell 1: plf rating | -0.15 | 0.16 | -0.96 | 0.337 |  | 0.86 [0.63, 1.17] |
| Combined Modell 1: ca score | -0.09 | 0.17 | -0.54 | 0.589 |  | 0.91 [0.65, 1.28] |
|  |  |  |  |  |  |  |
| --- | --- | --- | --- | --- | --- | --- |
| ^\*^ \* p < .05, \*\* p < .01, \*\*\* p < .001 |  |  |  |  |  |  |

While the 31+ age bracket was significantly associated with Pre-existing antecedents in the earlier bivariate logistic regression analyses, it was not significant in the combined dataset. To explore whether this change was due to interactions with other predictors or the reduced sample size in the combined dataset, a separate bivariate logistic regression analysis was conducted using only the age at assessment predictor within the combined dataset.

###### Age at assessment

Code

```
combined1_emergent_matched_age_glm <- glm(risk ~ age, data = combined1_emergent_matched, family = binomial)

table.function(combined1_emergent_matched_age_glm, "Combined model 1") |>
  kable(align = "lcc") |>
  kable_styling(full_width = FALSE, position = "left") |>
  add_footnote("* p < .05, ** p < .01, *** p < .001", notation = "symbol")
```

Table 79: Bivariate logistic regression results examining the association between the age at assessment and Emergent antecedents, comparing the antecedent sample to the matched control sample (n = 675).

| Predictor | Estimate | Std.Error | z | p\_value | Significance | Odds Ratio [95% CI] |
| --- | --- | --- | --- | --- | --- | --- |
| Combined model 1: Intercept | -0.03 | 0.09 | -0.31 | 0.754 |  | 0.97 [0.82, 1.16] |
| Combined model 1: Age 22-25 | 0.19 | 0.20 | 0.95 | 0.342 |  | 1.21 [0.82, 1.81] |
| Combined model 1: Age 26-30 | 0.09 | 0.37 | 0.25 | 0.803 |  | 1.1 [0.53, 2.29] |
| Combined model 1: Age 31+ | 0.32 | 0.45 | 0.70 | 0.483 |  | 1.37 [0.57, 3.41] |
|  |  |  |  |  |  |  |
| --- | --- | --- | --- | --- | --- | --- |
| ^\*^ \* p < .05, \*\* p < .01, \*\*\* p < .001 |  |  |  |  |  |  |

To evaluate whether the multivariate model provided a better fit than the null model, model comparisons were conducted using Nagelkerke’s pseudo-R². Chi-square tests were then used to assess whether the increase in explained variance was statistically significant.

Code

```
combined1_emergent_matched_null <- glm(risk ~ 1, data = combined1_emergent_matched, family = binomial)

model_comparison_nagelkerke(combined1_emergent_matched_null, combined1_emergent_matched_glm) %>%
  kable(align = "lcc") |>
  kable_styling(full_width = FALSE, position = "left")
```

Table 80: Model comparison between the first combined multivariate model and the null model in predicting Emergent antecedents, comparing the antecedent sample to the matched control sample.

| Comparison | Nagelkerke\_R2 | Chi2 | df | p\_value | Significance |
| --- | --- | --- | --- | --- | --- |
| Model vs Null | 0.065 | 33.88678 | 6 | < .001 | \*\*\* |

- Multivariate regression analysis
- Bivariate analysis
- Model comparison

Fits a multivariate logistic regression and presents the results in a formatted table.

Code

```
combined1_emergent_nonmatched_glm <- glm(risk ~ sex + age + plf + ca, data = combined1_emergent_nonmatched, family = binomial)

table.function(combined1_emergent_nonmatched_glm, "Combined Modell 1") |>
  kable(align = "lcc") |>
  kable_styling(full_width = FALSE, position = "left") |>
  add_footnote("* p < .05, ** p < .01, *** p < .001", notation = "symbol")
```

Table 81: Multivariate logistic regression results examining the association between demographic and assessment predictors and Emergent antecedents, comparing the antecedent sample to the non-matched control sample (n = 959).

| Predictor | Estimate | Std.Error | z | p\_value | Significance | Odds Ratio [95% CI] |
| --- | --- | --- | --- | --- | --- | --- |
| Combined Modell 1: Intercept | 0.44 | 0.43 | 1.03 | 0.305 |  | 1.55 [0.67, 3.61] |
| Combined Modell 1: Female | -1.26 | 0.29 | -4.34 | < .001 | \*\*\* | 0.28 [0.16, 0.49] |
| Combined Modell 1: Age 22-25 | 0.14 | 0.18 | 0.80 | 0.423 |  | 1.15 [0.81, 1.63] |
| Combined Modell 1: Age 26-30 | 0.24 | 0.34 | 0.72 | 0.475 |  | 1.27 [0.65, 2.44] |
| Combined Modell 1: Age 31+ | 0.05 | 0.37 | 0.14 | 0.890 |  | 1.05 [0.49, 2.16] |
| Combined Modell 1: plf rating | -0.04 | 0.13 | -0.31 | 0.756 |  | 0.96 [0.74, 1.24] |
| Combined Modell 1: ca score | -0.38 | 0.15 | -2.53 | 0.011 | \* | 0.69 [0.51, 0.92] |
|  |  |  |  |  |  |  |
| --- | --- | --- | --- | --- | --- | --- |
| ^\*^ \* p < .05, \*\* p < .01, \*\*\* p < .001 |  |  |  |  |  |  |

While the 31+ age bracket was significantly associated with Pre-existing antecedents in the earlier bivariate logistic regression analyses, it was not significant in the combined dataset. To explore whether this change was due to interactions with other predictors or the reduced sample size in the combined dataset, a separate bivariate logistic regression analysis was conducted using only the age at assessment predictor within the combined dataset.

###### Age at assessment

Code

```
combined1_emergent_nonmatched_age_glm <- glm(risk ~ age, data = combined1_emergent_nonmatched, family = binomial)

table.function(combined1_emergent_nonmatched_age_glm, "Combined model 1") |>
  kable(align = "lcc") |>
  kable_styling(full_width = FALSE, position = "left") |>
  add_footnote("* p < .05, ** p < .01, *** p < .001", notation = "symbol")
```

Table 82: Bivariate logistic regression results examining the association between the age at assessment and Emergent antecedents, comparing the antecedent sample to the non-matched control sample (n = 959).

| Predictor | Estimate | Std.Error | z | p\_value | Significance | Odds Ratio [95% CI] |
| --- | --- | --- | --- | --- | --- | --- |
| Combined model 1: Intercept | -0.63 | 0.08 | -7.97 | < .001 | \*\*\* | 0.53 [0.46, 0.62] |
| Combined model 1: Age 22-25 | 0.13 | 0.17 | 0.72 | 0.469 |  | 1.14 [0.8, 1.59] |
| Combined model 1: Age 26-30 | 0.18 | 0.33 | 0.55 | 0.581 |  | 1.2 [0.62, 2.27] |
| Combined model 1: Age 31+ | 0.07 | 0.37 | 0.19 | 0.853 |  | 1.07 [0.5, 2.18] |
|  |  |  |  |  |  |  |
| --- | --- | --- | --- | --- | --- | --- |
| ^\*^ \* p < .05, \*\* p < .01, \*\*\* p < .001 |  |  |  |  |  |  |

To evaluate whether the multivariate model provided a better fit than the null model, model comparisons were conducted using Nagelkerke’s pseudo-R². Chi-square tests were then used to assess whether the increase in explained variance was statistically significant.

Code

```
combined1_emergent_nonmatched_null <- glm(risk ~ 1, data = combined1_emergent_nonmatched, family = binomial)

model_comparison_nagelkerke(combined1_emergent_nonmatched_null, combined1_emergent_nonmatched_glm) %>%
  kable(align = "lcc") |>
  kable_styling(full_width = FALSE, position = "left")
```

Table 83: Model comparison between the first combined multivariate model and the null model in predicting Emergent antecedents, comparing the antecedent sample to the non-matched control sample.

| Comparison | Nagelkerke\_R2 | Chi2 | df | p\_value | Significance |
| --- | --- | --- | --- | --- | --- |
| Model vs Null | 0.043 | 30.44503 | 6 | < .001 | \*\*\* |

## Combined dataset 2: Age at assessment, sex, cognitive ability, and suitability for military command

**age**: Categorical variable grouping age at the time of first completed military selection assessment into four intervals:

1. 18-21
2. 22-25
3. 26-30
4. 31 and above (31+).

The reference level is 18-21 years.

**sex**: Binary categorical variable representing sex, with levels coded as 1 = male and 2 = female. The reference category in the analysis is male.

**ca**: Ordinal variable representing cognitive ability scores on a three-point scale (1-3). Treated as a continuous variable in the logistic regression analyses.

**smc**: Ordinal variable representing suitability for military command on a three-point scale (1-3). Treated as a continuous variable in the logistic regression analyses.

**sample**: Categorical variable indicating the sample to which the individual belongs:

1. Pre-existing antecedent sample
2. Pre-existing matched control sample
3. Emergent antecedent sample
4. Emergent matched control sample
5. Non-matched control sample

**risk**: Binary (dummy coded) outcome variable indicating whether the individual belongs to a antecedent sample (1) or a control sample (0).

### Data Preparation and Visualization

Code

```
combined2_raw <- read_csv2("Combined_dataset_2.csv.", show_col_types = FALSE)
```

- Data preprocessing
- Data visualization
- Correlation table

The imported data were preprocessed by filtering out unused variables, translating and renaming variables for improved readability, and converting relevant variables into factors.

The first five rows of the preprocessed dataset are shown below.

Code

```
combined2_dataset <- combined2_raw %>%
  
  rename(
    sample = "Grupp",
    sex = "Kön",
    smc = "Befl Agg",
    ca = "Gkap Agg",
    age = "Ålderagg",
    risk = "Utfall") %>%
  
  mutate(
    sample = as.numeric(sample),
    
    sample_group = case_when(
      sample %in% c(1, 3) ~ "Total investigation sample",
      sample %in% c(2, 4) ~ "Matched control sample",
      sample == 5 ~ "Non-matched control sample",
      TRUE ~ NA_character_),
    
    sample = factor(sample,
                    levels = 1:5,
                    labels = c("Pre-existing antecedent sample",
                               "Pre-existing matched control sample",
                               "Emergent antecedent sample",
                               "Emergent matched control sample",
                               "Non-matched control sample")),
    sex = factor(sex,
                    levels = 1:2,
                    labels = c("Male", "Female")),
    
    smc = as.numeric(smc),
    ca = as.numeric(ca),
    age = factor(age,
                 levels = 1:4,
                 labels = c("18-21", "22-25", "26-30", "31+")),
    risk = as.numeric(risk),
    risk = factor(risk,
                    levels = 0:1,
                    labels = c("Control", "Risk")),

    age_num = as.numeric(age),
    sex_num = as.numeric(sex) - 1,  
    risk_num = as.numeric(risk) - 1)
```

The table below displays the number of individuals per sample in the combined dataset, highlighting the reduction in sample size resulting from merging the single-predictor datasets into one combined dataset. Since all demographic predictors include 650 complete cases, the comparison focuses on the assessment variables, which vary in completeness across samples.

Code

```
smc_counts <- smc_dataset %>%
  filter(!is.na(smc)) %>%
  count(sample, name = "Suitability for military command")

combined2_counts <- combined2_dataset %>%
  count(sample, name = "Combined dataset 2")

comined2_summary <- full_join(ca_counts, smc_counts, by = "sample") %>%
  full_join(combined2_counts, by = "sample")

comined2_summary %>%
  arrange(sample) %>%
  kable() %>%
  kable_styling(full_width = FALSE, position = "left")
```

Table 84: Number of individuals per sample in each dataset, including the single-predictor datasets and the second combined dataset with multiple predictors.

| sample | Cognitive ability | Suitability for military command | Combined dataset 2 |
| --- | --- | --- | --- |
| Pre-existing antecedent sample | 286 | 227 | 218 |
| Pre-existing matched control sample | 281 | 231 | 217 |
| Emergent antecedent sample | 360 | 293 | 287 |
| Emergent matched control sample | 355 | 303 | 283 |
| Non-matched control sample | 643 | 537 | 521 |

The tables below display heterogeneous correlation matrices (maximum-likelihood estimates) for each sample in combined dataset 2. Correlations were estimated using the hetcor() function from the polycor package, which computes Pearson correlations for continuous-continuous pairs, polyserial correlations for continuous-ordinal/dichotomous pairs, and polychoric correlations for ordinal-ordinal pairs.

Age and sex were treated as ordered variables to ensure appropriate estimation of ordinal associations. Each table includes the correlation coefficient, the correlation type (in parentheses), and significance levels based on asymptotic z-tests (p < .05 *, p < .01 **, p < .001*** ). The sample size (N) reflects the number of complete cases used in each estimation.

Code

```
options(kableExtra.html.bsTable = FALSE)

combined2_dataset %>%

  filter(!is.na(sample_group)) %>%  
  group_by(sample_group) %>%
  group_walk(~ {

    .x_ord <- .x %>%
      mutate(
        age = ordered(age, levels = c("18-21","22-25","26-30","31+")),
        sex = ordered(sex, levels = c("Male","Female")))
    
    res <- .x_ord %>%
      select(age, sex, ca, smc) %>%
      drop_na() %>%
      as.data.frame() %>%
      hetcor(ML = TRUE)
    
    rmat <- res$correlations
    tmat <- res$type
    se   <- res$std.errors

    zmat <- rmat / se
    pmat <- 2 * pnorm(-abs(zmat))

    stars <- function(p)
      ifelse(is.na(p), "",
             ifelse(p < .001, "***",
             ifelse(p < .01,  "**",
             ifelse(p < .05, "*", ""))))

    rround <- round(rmat, 2)
    lab <- matrix(
      sprintf("%.2f (%s)%s",
              rround,
              ifelse(tmat == "", "—", tmat),
              stars(pmat)),
      nrow = nrow(rmat), dimnames = dimnames(rmat))
    diag(lab) <- ""
    lab[upper.tri(lab)] <- ""

    N <- .x_ord %>% drop_na(smc, ca, age, sex) %>% nrow()
    
    kable(
      lab, align = "l",
      caption = paste0(
        "Combined dataset 1: Heterogeneous correlations (ML) — ",
        unique(.y$sample_group),
        " (N = ", N, ")")) %>%
      footnote(
        general = paste(
          "Note. Correlations are maximum-likelihood estimates:",
          "Pearson between continuous variables;",
          "polyserial between continuous and ordinal/dichotomous variables;",
          "polychoric between ordinal variables (tetrachoric when both dichotomous).",
          "Age coded as ordered categories (18-21 < 22-25 < 26-30 < 31+);",
          "sex treated as an ordered dichotomous variable solely for estimation.",
          "p < .05 *, p < .01 **, p < .001 ***."),
        general_title = "") %>%
      print()})
```

Combined dataset 1: Heterogeneous correlations (ML) — Matched control sample (N = 500)

|  | age | sex | ca | smc |
| --- | --- | --- | --- | --- |
| age |  |  |  |  |
| sex | 0.04 (Polychoric) |  |  |  |
| ca | 0.11 (Polyserial) | -0.19 (Polyserial)\*\* |  |  |
| smc | 0.04 (Polyserial) | 0.09 (Polyserial) | 0.14 (Pearson)\*\* |  |
|  |  |  |  |  |
| --- | --- | --- | --- | --- |
| Note. Correlations are maximum-likelihood estimates: Pearson between continuous variables; polyserial between continuous and ordinal/dichotomous variables; polychoric between ordinal variables (tetrachoric when both dichotomous). Age coded as ordered categories (18-21 < 22-25 < 26-30 < 31+); sex treated as an ordered dichotomous variable solely for estimation. p < .05 *, p < .01 **, p < .001*** . |  |  |  |  |

Combined dataset 1: Heterogeneous correlations (ML) — Non-matched control sample (N = 521)

|  | age | sex | ca | smc |
| --- | --- | --- | --- | --- |
| age |  |  |  |  |
| sex | 0.01 (Polychoric) |  |  |  |
| ca | 0.09 (Polyserial) | -0.07 (Polyserial) |  |  |
| smc | -0.01 (Polyserial) | -0.06 (Polyserial) | 0.14 (Pearson)\*\* |  |
|  |  |  |  |  |
| --- | --- | --- | --- | --- |
| Note. Correlations are maximum-likelihood estimates: Pearson between continuous variables; polyserial between continuous and ordinal/dichotomous variables; polychoric between ordinal variables (tetrachoric when both dichotomous). Age coded as ordered categories (18-21 < 22-25 < 26-30 < 31+); sex treated as an ordered dichotomous variable solely for estimation. p < .05 *, p < .01 **, p < .001*** . |  |  |  |  |

Combined dataset 1: Heterogeneous correlations (ML) — Total investigation sample (N = 505)

|  | age | sex | ca | smc |
| --- | --- | --- | --- | --- |
| age |  |  |  |  |
| sex | 0.07 (Polychoric) |  |  |  |
| ca | 0.04 (Polyserial) | -0.23 (Polyserial)\* |  |  |
| smc | 0.01 (Polyserial) | -0.18 (Polyserial) | 0.11 (Pearson)\* |  |
|  |  |  |  |  |
| --- | --- | --- | --- | --- |
| Note. Correlations are maximum-likelihood estimates: Pearson between continuous variables; polyserial between continuous and ordinal/dichotomous variables; polychoric between ordinal variables (tetrachoric when both dichotomous). Age coded as ordered categories (18-21 < 22-25 < 26-30 < 31+); sex treated as an ordered dichotomous variable solely for estimation. p < .05 *, p < .01 **, p < .001*** . |  |  |  |  |

### Data analysis

- Pre-existing antecedents - Matched control sample
- Pre-existing antecedents - Non-matched control sample
- Emergent antecedents - Matched control sample
- Emergent antecedents - Non-matched control sample

- Multivariate regression analysis
- Bivariate analysis
- Model comparison

Fit a multivariate logistic regression and presents the results in a formatted table.

Code

```
combined2_pre_matched_glm <- glm(risk ~ sex + age + smc + ca, data = combined2_pre_matched, family = binomial)

table.function(combined2_pre_matched_glm, "Combined model 2") |>
  kable(align = "lcc") |>
  kable_styling(full_width = FALSE, position = "left") |>
  add_footnote("* p < .05, ** p < .01, *** p < .001", notation = "symbol")
```

Table 85: Multivariate logistic regression results examining the association between demographic and assessment predictors and pre-existing antecedents, comparing the antecedent sample to the total matched control sample (n = 435).

| Predictor | Estimate | Std.Error | z | p\_value | Significance | Odds Ratio [95% CI] |
| --- | --- | --- | --- | --- | --- | --- |
| Combined model 2: Intercept | 1.44 | 0.67 | 2.15 | 0.032 | \* | 4.21 [1.15, 15.93] |
| Combined model 2: Female | -1.43 | 0.49 | -2.92 | 0.003 | \*\* | 0.24 [0.08, 0.59] |
| Combined model 2: Age 22-25 | 0.95 | 0.24 | 3.92 | < .001 | \*\*\* | 2.58 [1.61, 4.17] |
| Combined model 2: Age 26-30 | 1.65 | 0.42 | 3.88 | < .001 | \*\*\* | 5.19 [2.36, 12.69] |
| Combined model 2: Age 31+ | 15.65 | 514.24 | 0.03 | 0.976 |  | 6292795.22 [0, NA] |
| Combined model 2: smc rating | -0.46 | 0.22 | -2.08 | 0.038 | \* | 0.63 [0.4, 0.97] |
| Combined model 2: ca score | -0.32 | 0.22 | -1.46 | 0.146 |  | 0.73 [0.47, 1.11] |
|  |  |  |  |  |  |  |
| --- | --- | --- | --- | --- | --- | --- |
| ^\*^ \* p < .05, \*\* p < .01, \*\*\* p < .001 |  |  |  |  |  |  |

While the 31+ age bracket was significantly associated with Pre-existing antecedents in the earlier bivariate logistic regression analyses, it was not significant in the combined dataset. Similarly, although suitability for military command was significantly associated with both pre-existing and Emergent antecedents in the bivariate logistic regression analyses, it was not a significant predictor in the combined dataset when predicting Emergent antecedents or when predicting Pre-existing antecedents using the non-matched control comparison.

To explore whether this change was due to interactions with other predictors or the reduced sample size in the combined dataset, separate bivariate logistic regression analyses were conducted using only age at assessment and suitability for military command as predictors, respectively.

###### Age at assessment

Code

```
combined2_pre_matched_age_glm <- glm(risk ~ age, data = combined2_pre_matched, family = binomial)

table.function(combined2_pre_matched_age_glm, "Combined model 2") |>
  kable(align = "lcc") |>
  kable_styling(full_width = FALSE, position = "left") |>
  add_footnote("* p < .05, ** p < .01, *** p < .001", notation = "symbol")
```

Table 86: Bivariate logistic regression results examining the association between the age at assessment and pre-existing antecedents, comparing the antecedent sample to the matched control sample (n = 435).

| Predictor | Estimate | Std.Error | z | p\_value | Significance | Odds Ratio [95% CI] |
| --- | --- | --- | --- | --- | --- | --- |
| Combined model 2: Intercept | -0.39 | 0.12 | -3.23 | 0.001 | \*\* | 0.68 [0.53, 0.86] |
| Combined model 2: Age 22-25 | 0.94 | 0.24 | 3.98 | < .001 | \*\*\* | 2.56 [1.62, 4.1] |
| Combined model 2: Age 26-30 | 1.64 | 0.42 | 3.92 | < .001 | \*\*\* | 5.16 [2.37, 12.49] |
| Combined model 2: Age 31+ | 15.95 | 514.56 | 0.03 | 0.975 |  | 8487810.34 [0, NA] |
|  |  |  |  |  |  |  |
| --- | --- | --- | --- | --- | --- | --- |
| ^\*^ \* p < .05, \*\* p < .01, \*\*\* p < .001 |  |  |  |  |  |  |

###### Suitability for military command

Code

```
combined2_pre_matched_smc_glm <- glm(risk ~ smc, data = combined2_pre_matched, family = binomial)

table.function(combined2_pre_matched_smc_glm, "Combined model 2") |>
  kable(align = "lcc") |>
  kable_styling(full_width = FALSE, position = "left") |>
  add_footnote("* p < .05, ** p < .01, *** p < .001", notation = "symbol")
```

Table 87: Bivariate logistic regression results examining the association between the suitability for military command rating and pre-existing antecedents, comparing the antecedent sample to the matched control sample (n = 435).

| Predictor | Estimate | Std.Error | z | p\_value | Significance | Odds Ratio [95% CI] |
| --- | --- | --- | --- | --- | --- | --- |
| Combined model 2: Intercept | 1.09 | 0.46 | 2.38 | 0.017 | \* | 2.97 [1.22, 7.36] |
| Combined model 2: smc rating | -0.51 | 0.21 | -2.43 | 0.015 | \* | 0.6 [0.4, 0.9] |
|  |  |  |  |  |  |  |
| --- | --- | --- | --- | --- | --- | --- |
| ^\*^ \* p < .05, \*\* p < .01, \*\*\* p < .001 |  |  |  |  |  |  |

To evaluate whether the multivariate model provided a better fit than the null model, model comparisons were conducted using Nagelkerke’s pseudo-R². Chi-square tests were then used to assess whether the increase in explained variance was statistically significant.

Code

```
combined2_pre_matched_null <- glm(risk ~ 1, data = combined2_pre_matched, family = binomial)

model_comparison_nagelkerke(combined2_pre_matched_null, combined2_pre_matched_glm) %>%
  kable(align = "lcc") |>
  kable_styling(full_width = FALSE, position = "left")
```

Table 88: Model comparison between the second combined multivariate model and the null model in predicting Pre-existing antecedents, comparing the antecedent sample to the matched control sample.

| Comparison | Nagelkerke\_R2 | Chi2 | df | p\_value | Significance |
| --- | --- | --- | --- | --- | --- |
| Model vs Null | 0.166 | 57.7273 | 6 | < .001 | \*\*\* |

- Multivariate regression analysis
- Bivariate analysis
- Model comparison

Fit a multivariate logistic regression and presents the results in a formatted table.

Code

```
combined2_pre_nonmatched_glm <- glm(risk ~ sex + age + smc + ca, data = combined2_pre_nonmatched, family = binomial)

table.function(combined2_pre_nonmatched_glm, "Combined model 2") |>
  kable(align = "lcc") |>
  kable_styling(full_width = FALSE, position = "left") |>
  add_footnote("* p < .05, ** p < .01, *** p < .001", notation = "symbol")
```

Table 89: Multivariate logistic regression results examining the association between demographic and assessment predictors and pre-existing antecedents, comparing the antecedent sample to the total non-matched control sample (n = 739).

| Predictor | Estimate | Std.Error | z | p\_value | Significance | Odds Ratio [95% CI] |
| --- | --- | --- | --- | --- | --- | --- |
| Combined model 2: Intercept | 0.75 | 0.53 | 1.41 | 0.158 |  | 2.13 [0.75, 6.1] |
| Combined model 2: Female | -1.75 | 0.44 | -3.94 | < .001 | \*\*\* | 0.17 [0.07, 0.38] |
| Combined model 2: Age 22-25 | 0.91 | 0.20 | 4.61 | < .001 | \*\*\* | 2.49 [1.69, 3.68] |
| Combined model 2: Age 26-30 | 1.62 | 0.32 | 5.06 | < .001 | \*\*\* | 5.03 [2.71, 9.53] |
| Combined model 2: Age 31+ | 0.62 | 0.47 | 1.33 | 0.183 |  | 1.86 [0.71, 4.58] |
| Combined model 2: smc rating | -0.29 | 0.17 | -1.72 | 0.085 |  | 0.74 [0.53, 1.04] |
| Combined model 2: ca score | -0.52 | 0.18 | -2.93 | 0.003 | \*\* | 0.59 [0.42, 0.84] |
|  |  |  |  |  |  |  |
| --- | --- | --- | --- | --- | --- | --- |
| ^\*^ \* p < .05, \*\* p < .01, \*\*\* p < .001 |  |  |  |  |  |  |

While the 31+ age bracket was significantly associated with Pre-existing antecedents in the earlier bivariate logistic regression analyses, it was not significant in the combined dataset. Similarly, although suitability for military command was significantly associated with both pre-existing and Emergent antecedents in the bivariate logistic regression analyses, it was not a significant predictor in the combined dataset when predicting Emergent antecedents or when predicting Pre-existing antecedents using the non-matched control comparison.

To explore whether this change was due to interactions with other predictors or the reduced sample size in the combined dataset, separate bivariate logistic regression analyses were conducted using only age at assessment and suitability for military command as predictors, respectively.

###### Age at assessment

Code

```
combined2_pre_nonmatched_age_glm <- glm(risk ~ age, data = combined2_pre_nonmatched, family = binomial)

table.function(combined2_pre_nonmatched_age_glm, "Combined model 2") |>
  kable(align = "lcc") |>
  kable_styling(full_width = FALSE, position = "left") |>
  add_footnote("* p < .05, ** p < .01, *** p < .001", notation = "symbol")
```

Table 90: Bivariate logistic regression results examining the association between the age at assessment and pre-existing antecedents, comparing the antecedent sample to the non-matched control sample (n = 739).

| Predictor | Estimate | Std.Error | z | p\_value | Significance | Odds Ratio [95% CI] |
| --- | --- | --- | --- | --- | --- | --- |
| Combined model 2: Intercept | -1.22 | 0.11 | -11.58 | < .001 | \*\*\* | 0.29 [0.24, 0.36] |
| Combined model 2: Age 22-25 | 0.88 | 0.19 | 4.57 | < .001 | \*\*\* | 2.41 [1.65, 3.51] |
| Combined model 2: Age 26-30 | 1.51 | 0.31 | 4.91 | < .001 | \*\*\* | 4.53 [2.49, 8.36] |
| Combined model 2: Age 31+ | 0.74 | 0.46 | 1.60 | 0.110 |  | 2.09 [0.81, 5.08] |
|  |  |  |  |  |  |  |
| --- | --- | --- | --- | --- | --- | --- |
| ^\*^ \* p < .05, \*\* p < .01, \*\*\* p < .001 |  |  |  |  |  |  |

###### Suitability for military command

Code

```
combined2_pre_nonmatched_smc_glm <- glm(risk ~ smc, data = combined2_pre_nonmatched, family = binomial)

table.function(combined2_pre_nonmatched_smc_glm, "Combined model 2") |>
  kable(align = "lcc") |>
  kable_styling(full_width = FALSE, position = "left") |>
  add_footnote("* p < .05, ** p < .01, *** p < .001", notation = "symbol")
```

Table 91: Bivariate logistic regression results examining the association between the suitability for military command rating and pre-existing antecedents, comparing the antecedent sample to the non-matched control sample (n = 739).

| Predictor | Estimate | Std.Error | z | p\_value | Significance | Odds Ratio [95% CI] |
| --- | --- | --- | --- | --- | --- | --- |
| Combined model 2: Intercept | -0.18 | 0.35 | -0.53 | 0.595 |  | 0.83 [0.42, 1.65] |
| Combined model 2: smc rating | -0.32 | 0.16 | -2.02 | 0.044 | \* | 0.72 [0.53, 0.99] |
|  |  |  |  |  |  |  |
| --- | --- | --- | --- | --- | --- | --- |
| ^\*^ \* p < .05, \*\* p < .01, \*\*\* p < .001 |  |  |  |  |  |  |

To evaluate whether the multivariate model provided a better fit than the null model, model comparisons were conducted using Nagelkerke’s pseudo-R². Chi-square tests were then used to assess whether the increase in explained variance was statistically significant.

Code

```
combined2_pre_nonmatched_null <- glm(risk ~ 1, data = combined2_pre_nonmatched, family = binomial)

model_comparison_nagelkerke(combined2_pre_nonmatched_null, combined2_pre_nonmatched_glm) %>%
  kable(align = "lcc") |>
  kable_styling(full_width = FALSE, position = "left")
```

Table 92: Model comparison between the second combined multivariate model and the null model in predicting Pre-existing antecedents, comparing the antecedent sample to the non-matched control sample.

| Comparison | Nagelkerke\_R2 | Chi2 | df | p\_value | Significance |
| --- | --- | --- | --- | --- | --- |
| Model vs Null | 0.134 | 73.35707 | 6 | < .001 | \*\*\* |

- Multivariate regression analysis
- Bivariate analysis
- Model comparison

Fits a multivariate logistic regression and presents the results in a formatted table.

Code

```
combined2_emergent_matched_glm <- glm(risk ~ sex + age + smc + ca, data = combined2_emergent_matched, family = binomial)

table.function(combined2_emergent_matched_glm, "Combined model 2") |>
  kable(align = "lcc") |>
  kable_styling(full_width = FALSE, position = "left") |>
  add_footnote("* p < .05, ** p < .01, *** p < .001", notation = "symbol")
```

Table 93: Multivariate logistic regression results examining the association between demographic and assessment predictors and Emergent antecedents, comparing the antecedent sample to the total matched control sample (n = 570).

| Predictor | Estimate | Std.Error | z | p\_value | Significance | Odds Ratio [95% CI] |
| --- | --- | --- | --- | --- | --- | --- |
| Combined model 2: Intercept | 0.84 | 0.56 | 1.51 | 0.132 |  | 2.32 [0.78, 6.99] |
| Combined model 2: Female | -1.38 | 0.32 | -4.33 | < .001 | \*\*\* | 0.25 [0.13, 0.46] |
| Combined model 2: Age 22-25 | 0.21 | 0.22 | 0.94 | 0.346 |  | 1.23 [0.8, 1.92] |
| Combined model 2: Age 26-30 | 0.46 | 0.43 | 1.08 | 0.279 |  | 1.59 [0.69, 3.73] |
| Combined model 2: Age 31+ | 2.05 | 0.79 | 2.60 | 0.009 | \*\* | 7.77 [2, 51.74] |
| Combined model 2: smc rating | -0.36 | 0.19 | -1.94 | 0.053 |  | 0.7 [0.48, 1] |
| Combined model 2: ca score | -0.01 | 0.18 | -0.03 | 0.977 |  | 0.99 [0.69, 1.43] |
|  |  |  |  |  |  |  |
| --- | --- | --- | --- | --- | --- | --- |
| ^\*^ \* p < .05, \*\* p < .01, \*\*\* p < .001 |  |  |  |  |  |  |

While the 31+ age bracket was significantly associated with Pre-existing antecedents in the earlier bivariate logistic regression analyses, it was not significant in the combined dataset. Similarly, although suitability for military command was significantly associated with both pre-existing and Emergent antecedents in the bivariate logistic regression analyses, it was not a significant predictor in the combined dataset when predicting Emergent antecedents or when predicting Pre-existing antecedents using the non-matched control comparison.

To explore whether this change was due to interactions with other predictors or the reduced sample size in the combined dataset, separate bivariate logistic regression analyses were conducted using only age at assessment and suitability for military command as predictors, respectively.

###### Age at assessment

Code

```
combined2_emergent_matched_age_glm <- glm(risk ~ age, data = combined2_emergent_matched, family = binomial)

table.function(combined2_emergent_matched_age_glm, "Combined model 2") |>
  kable(align = "lcc") |>
  kable_styling(full_width = FALSE, position = "left") |>
  add_footnote("* p < .05, ** p < .01, *** p < .001", notation = "symbol")
```

Table 94: Bivariate logistic regression results examining the association between the age at assessment and Emergent antecedents, comparing the antecedent sample to the matched control sample (n = 570).

| Predictor | Estimate | Std.Error | z | p\_value | Significance | Odds Ratio [95% CI] |
| --- | --- | --- | --- | --- | --- | --- |
| Combined model 2: Intercept | -0.07 | 0.10 | -0.73 | 0.467 |  | 0.93 [0.77, 1.13] |
| Combined model 2: Age 22-25 | 0.20 | 0.22 | 0.93 | 0.350 |  | 1.23 [0.8, 1.89] |
| Combined model 2: Age 26-30 | 0.22 | 0.41 | 0.55 | 0.579 |  | 1.25 [0.56, 2.82] |
| Combined model 2: Age 31+ | 1.86 | 0.77 | 2.42 | 0.016 | \* | 6.44 [1.73, 41.7] |
|  |  |  |  |  |  |  |
| --- | --- | --- | --- | --- | --- | --- |
| ^\*^ \* p < .05, \*\* p < .01, \*\*\* p < .001 |  |  |  |  |  |  |

###### Suitability for military command

Code

```
combined2_emergent_matched_smc_glm <- glm(risk ~ smc, data = combined2_emergent_matched, family = binomial)

table.function(combined2_emergent_matched_smc_glm, "Combined model 2") |>
  kable(align = "lcc") |>
  kable_styling(full_width = FALSE, position = "left") |>
  add_footnote("* p < .05, ** p < .01, *** p < .001", notation = "symbol")
```

Table 95: Bivariate logistic regression results examining the association between the suitability for military command rating and Emergent antecedents, comparing the antecedent sample to the matched control sample (n = 570).

| Predictor | Estimate | Std.Error | z | p\_value | Significance | Odds Ratio [95% CI] |
| --- | --- | --- | --- | --- | --- | --- |
| Combined model 2: Intercept | 0.75 | 0.39 | 1.90 | 0.057 |  | 2.12 [0.98, 4.63] |
| Combined model 2: smc rating | -0.34 | 0.18 | -1.91 | 0.056 |  | 0.71 [0.5, 1.01] |
|  |  |  |  |  |  |  |
| --- | --- | --- | --- | --- | --- | --- |
| ^\*^ \* p < .05, \*\* p < .01, \*\*\* p < .001 |  |  |  |  |  |  |

To evaluate whether the multivariate model provided a better fit than the null model, model comparisons were conducted using Nagelkerke’s pseudo-R². Chi-square tests were then used to assess whether the increase in explained variance was statistically significant.

Code

```
combined2_emergent_matched_null <- glm(risk ~ 1, data = combined2_emergent_matched, family = binomial)

model_comparison_nagelkerke(combined2_emergent_matched_null, combined2_emergent_matched_glm) %>%
  kable(align = "lcc") |>
  kable_styling(full_width = FALSE, position = "left")
```

Table 96: Model comparison between the second combined multivariate model and the null model in predicting Emergent antecedents, comparing the antecedent sample to the matched control sample.

| Comparison | Nagelkerke\_R2 | Chi2 | df | p\_value | Significance |
| --- | --- | --- | --- | --- | --- |
| Model vs Null | 0.08 | 35.23056 | 6 | < .001 | \*\*\* |

- Multivariate regression analysis
- Bivariate analysis
- Model comparison

Fits a multivariate logistic regression and presents the results in a formatted table.

Code

```
combined2_emergent_nonmatched_glm <- glm(risk ~ sex + age + smc + ca, data = combined2_emergent_nonmatched, family = binomial)

table.function(combined2_emergent_nonmatched_glm, "Combined model 2") |>
  kable(align = "lcc") |>
  kable_styling(full_width = FALSE, position = "left") |>
  add_footnote("* p < .05, ** p < .01, *** p < .001", notation = "symbol")
```

Table 97: Multivariate logistic regression results examining the association between demographic and assessment predictors and Emergent antecedents, comparing the antecedent sample to the total non-matched control sample (n = 808).

| Predictor | Estimate | Std.Error | z | p\_value | Significance | Odds Ratio [95% CI] |
| --- | --- | --- | --- | --- | --- | --- |
| Combined model 2: Intercept | 0.92 | 0.47 | 1.95 | 0.052 |  | 2.51 [1, 6.38] |
| Combined model 2: Female | -1.00 | 0.30 | -3.36 | < .001 | \*\*\* | 0.37 [0.2, 0.64] |
| Combined model 2: Age 22-25 | 0.17 | 0.19 | 0.87 | 0.383 |  | 1.18 [0.81, 1.72] |
| Combined model 2: Age 26-30 | 0.35 | 0.36 | 0.98 | 0.327 |  | 1.43 [0.69, 2.88] |
| Combined model 2: Age 31+ | 0.65 | 0.42 | 1.57 | 0.116 |  | 1.92 [0.84, 4.37] |
| Combined model 2: smc rating | -0.28 | 0.15 | -1.78 | 0.075 |  | 0.76 [0.56, 1.03] |
| Combined model 2: ca score | -0.38 | 0.16 | -2.45 | 0.014 | \* | 0.68 [0.5, 0.92] |
|  |  |  |  |  |  |  |
| --- | --- | --- | --- | --- | --- | --- |
| ^\*^ \* p < .05, \*\* p < .01, \*\*\* p < .001 |  |  |  |  |  |  |

While the 31+ age bracket was significantly associated with Pre-existing antecedents in the earlier bivariate logistic regression analyses, it was not significant in the combined dataset. Similarly, although suitability for military command was significantly associated with both pre-existing and Emergent antecedents in the bivariate logistic regression analyses, it was not a significant predictor in the combined dataset when predicting Emergent antecedents or when predicting Pre-existing antecedents using the non-matched control comparison.

To explore whether this change was due to interactions with other predictors or the reduced sample size in the combined dataset, separate bivariate logistic regression analyses were conducted using only age at assessment and suitability for military command as predictors, respectively.

###### Age at assessment

Code

```
combined2_emergent_nonmatched_age_glm <- glm(risk ~ age, data = combined2_emergent_nonmatched, family = binomial)

table.function(combined2_emergent_nonmatched_age_glm, "Combined model 2") |>
  kable(align = "lcc") |>
  kable_styling(full_width = FALSE, position = "left") |>
  add_footnote("* p < .05, ** p < .01, *** p < .001", notation = "symbol")
```

Table 98: Bivariate logistic regression results examining the association between the age at assessment and Emergent antecedents, comparing the antecedent sample to the non-matched control sample (n = 808).

| Predictor | Estimate | Std.Error | z | p\_value | Significance | Odds Ratio [95% CI] |
| --- | --- | --- | --- | --- | --- | --- |
| Combined model 2: Intercept | -0.65 | 0.09 | -7.59 | < .001 | \*\*\* | 0.52 [0.44, 0.62] |
| Combined model 2: Age 22-25 | 0.15 | 0.19 | 0.77 | 0.441 |  | 1.16 [0.79, 1.67] |
| Combined model 2: Age 26-30 | 0.25 | 0.36 | 0.70 | 0.486 |  | 1.28 [0.63, 2.55] |
| Combined model 2: Age 31+ | 0.57 | 0.41 | 1.40 | 0.161 |  | 1.77 [0.78, 3.98] |
|  |  |  |  |  |  |  |
| --- | --- | --- | --- | --- | --- | --- |
| ^\*^ \* p < .05, \*\* p < .01, \*\*\* p < .001 |  |  |  |  |  |  |

###### Suitability for military command

Code

```
combined2_emergent_nonmatched_smc_glm <- glm(risk ~ smc, data = combined2_emergent_nonmatched, family = binomial)

table.function(combined2_emergent_nonmatched_smc_glm, "Combined model 2") |>
  kable(align = "lcc") |>
  kable_styling(full_width = FALSE, position = "left") |>
  add_footnote("* p < .05, ** p < .01, *** p < .001", notation = "symbol")
```

Table 99: Bivariate logistic regression results examining the association between the suitability for military command rating and Emergent antecedents, comparing the antecedent sample to the non-matched control sample (n = 808).

| Predictor | Estimate | Std.Error | z | p\_value | Significance | Odds Ratio [95% CI] |
| --- | --- | --- | --- | --- | --- | --- |
| Combined model 2: Intercept | 0.02 | 0.33 | 0.05 | 0.960 |  | 1.02 [0.54, 1.94] |
| Combined model 2: smc rating | -0.29 | 0.15 | -1.91 | 0.056 |  | 0.75 [0.56, 1.01] |
|  |  |  |  |  |  |  |
| --- | --- | --- | --- | --- | --- | --- |
| ^\*^ \* p < .05, \*\* p < .01, \*\*\* p < .001 |  |  |  |  |  |  |

To evaluate whether the multivariate model provided a better fit than the null model, model comparisons were conducted using Nagelkerke’s pseudo-R². Chi-square tests were then used to assess whether the increase in explained variance was statistically significant.

Code

```
combined2_emergent_nonmatched_null <- glm(risk ~ 1, data = combined2_emergent_nonmatched, family = binomial)

model_comparison_nagelkerke(combined2_emergent_nonmatched_null, combined2_emergent_nonmatched_glm) %>%
  kable(align = "lcc") |>
  kable_styling(full_width = FALSE, position = "left")
```

Table 100: Model comparison between the second combined multivariate model and the null model in predicting Emergent antecedents, comparing the antecedent sample to the non-matched control sample.

| Comparison | Nagelkerke\_R2 | Chi2 | df | p\_value | Significance |
| --- | --- | --- | --- | --- | --- |
| Model vs Null | 0.042 | 25.23115 | 6 | < .001 | \*\*\* |
